# Understanding metric-related pitfalls in image analysis validation

**DOI:** 10.1038/s41592-023-02150-0

**Published:** 2024-02-12

**Authors:** Annika Reinke, Minu D. Tizabi, Michael Baumgartner, Matthias Eisenmann, Doreen Heckmann-Nötzel, A. Emre Kavur, Tim Rädsch, Carole H. Sudre, Laura Acion, Michela Antonelli, Tal Arbel, Spyridon Bakas, Arriel Benis, Matthew B. Blaschko, Florian Buettner, M. Jorge Cardoso, Veronika Cheplygina, Jianxu Chen, Evangelia Christodoulou, Beth A. Cimini, Gary S. Collins, Keyvan Farahani, Luciana Ferrer, Adrian Galdran, Bram Van Ginneken, Ben Glocker, Patrick Godau, Robert Haase, Daniel A. Hashimoto, Michael M. Hoffman, Merel Huisman, Fabian Isensee, Pierre Jannin, Charles E. Kahn, Dagmar Kainmueller, Bernhard Kainz, Alexandros Karargyris, Alan Karthikesalingam, Hannes Kenngott, Jens Kleesiek, Florian Kofler, Thijs Kooi, Annette Kopp-Schneider, Michal Kozubek, Anna Kreshuk, Tahsin Kurc, Bennett A. Landman, Geert Litjens, Amin Madani, Klaus Maier-Hein, Anne L. Martel, Peter Mattson, Erik Meijering, Bjoern Menze, Karel G.M. Moons, Henning Müller, Brennan Nichyporuk, Felix Nickel, Jens Petersen, Susanne M. Rafelski, Nasir Rajpoot, Mauricio Reyes, Michael A. Riegler, Nicola Rieke, Julio Saez-Rodriguez, Clara I. Sánchez, Shravya Shetty, Ronald M. Summers, Abdel A. Taha, Aleksei Tiulpin, Sotirios A. Tsaftaris, Ben Van Calster, Gaël Varoquaux, Ziv R. Yaniv, Paul F. Jäger, Lena Maier-Hein

**Affiliations:** German Cancer Research Center (DKFZ) Heidelberg, Division of Intelligent Medical Systems and HI Helmholtz Imaging, Germany; Faculty of Mathematics and Computer Science, Heidelberg University, Heidelberg, Germany; German Cancer Research Center (DKFZ) Heidelberg, Division of Intelligent Medical Systems, Germany; National Center for Tumor Diseases (NCT), NCT Heidelberg, University Medical Center Heidelberg, Germany; German Cancer Research Center (DKFZ) Heidelberg, Division of Medical Image Computing, Germany; German Cancer Research Center (DKFZ) Heidelberg, Division of Intelligent Medical Systems, Germany; German Cancer Research Center (DKFZ) Heidelberg, Division of Intelligent Medical Systems, Germany; National Center for Tumor Diseases (NCT), NCT Heidelberg, University Medical Center Heidelberg, Germany; HI Applied Computer Vision Lab, Division of Medical Image Computing; German Cancer Research Center (DKFZ) Heidelberg, Division of Intelligent Medical Systems, Germany; German Cancer Research Center (DKFZ) Heidelberg, Division of Intelligent Medical Systems and HI Helmholtz Imaging, Germany; MRC Unit for Lifelong Health and Ageing at UCL and Centre for Medical Image Computing, Department of Computer Science, University College London, London, UK; School of Biomedical Engineering and Imaging Science, King’s College London, London, UK; Instituto de Cálculo, CONICET – Universidad de Buenos Aires, Buenos Aires, Argentina; School of Biomedical Engineering and Imaging Science, King’s College London, London, UK; Centre for Medical Image Computing, University College London, London, UK; Centre for Intelligent Machines and MILA (Quebec Artificial Intelligence Institute), McGill University, Montreal, Canada; Division of Computational Pathology, Dept of Pathology & Laboratory Medicine, Indiana University School of Medicine, IU Health Information and Translational Sciences Building, Indianapolis, USA; Center for Biomedical Image Computing and Analytics (CBICA), University of Pennsylvania, Richards Medical Research Laboratories FL7, Philadelphia, PA, USA; Department of Digital Medical Technologies, Holon Institute of Technology, Holon, Israel; European Federation for Medical Informatics, Le Mont-sur-Lausanne, Switzerland; Center for Processing Speech and Images, Department of Electrical Engineering, KU Leuven, Leuven, Belgium; German Cancer Consortium (DKTK), partner site Frankfurt/Mainz, a partnership between DKFZ and UCT Frankfurt-Marburg, Germany; German Cancer Research Center (DKFZ) Heidelberg, Germany; Goethe University Frankfurt, Department of Medicine, Germany; Goethe University Frankfurt, Department of Informatics, Germany; Frankfurt Cancer Insititute, Germany; School of Biomedical Engineering and Imaging Science, King’s College London, London, UK; Department of Computer Science, IT University of Copenhagen, Copenhagen, Denmark; Leibniz-Institut für Analytische Wissenschaften – ISAS – e.V., Dortmund, Germany; German Cancer Research Center (DKFZ) Heidelberg, Division of Intelligent Medical Systems, Germany; Imaging Platform, Broad Institute of MIT and Harvard, Cambridge, Massachusetts, USA; Centre for Statistics in Medicine, University of Oxford, Oxford, UK; Center for Biomedical Informatics and Information Technology, National Cancer Institute, Bethesda, MD, USA; Instituto de Investigación en Ciencias de la Computación (ICC), CONICET-UBA, Ciudad Universitaria, Ciudad Autónoma de Buenos Aires, Argentina; Universitat Pompeu Fabra, Barcelona, Spain; University of Adelaide, Adelaide, Australia; Fraunhofer MEVIS, Bremen, Germany; Radboud Institute for Health Sciences, Radboud University Medical Center, Nijmegen, The Netherlands; Department of Computing, Imperial College London, London, UK; German Cancer Research Center (DKFZ) Heidelberg, Division of Intelligent Medical Systems, Germany; Faculty of Mathematics and Computer Science, Heidelberg University , Heidelberg, Germany; and National Center for Tumor Diseases (NCT), NCT Heidelberg, a partnership between DKFZ and University Medical Center Heidelberg, Germany; Center for Scalable Data Analytics and Artificial Intelligence (ScaDS.AI), Leipzig University, Leipzig, Germany; DFG Cluster of Excellence “Physics of Life”, Technische Universität (TU) Dresden, Dresden, Germany; Center for Systems Biology, Dresden, Germany; Department of Surgery, Perelman School of Medicine, Philadelphia, PA, USA; General Robotics Automation Sensing and Perception Laboratory, School of Engineering and Applied Science, University of Pennsylvania, Philadelphia, PA, USA; Princess Margaret Cancer Centre, University Health Network, Toronto, Canada; Department of Medical Biophysics, University of Toronto, Toronto, Canada; Department of Computer Science, University of Toronto, Toronto, Canada; Vector Institute for Artificial Intelligence, Toronto, Canada; Department of Radiology and Nuclear Medicine, Radboud University Medical Center, Nijmegen, The Netherlands; German Cancer Research Center (DKFZ) Heidelberg, Division of Medical Image Computing and HI Applied Computer Vision Lab, Germany; Laboratoire Traitement du Signal et de l’Image – UMR_S 1099, Université de Rennes 1, Rennes, France; INSERM, Paris Cedex, France; Department of Radiology and Institute for Biomedical Informatics, University of Pennsylvania, Philadelphia, PA, USA; Max-Delbrück Center for Molecular Medicine in the Helmholtz Association (MDC), Biomedical Image Analysis and HI Helmholtz Imaging, Berlin, Germany; University of Potsdam, Digital Engineering Faculty, Potsdam, Germany; Department of Computing, Faculty of Engineering, Imperial College London, London, UK; Department AIBE, Friedrich-Alexander-Universität (FAU), Erlangen-Nürnberg, Germany; IHU Strasbourg, Strasbourg, France; Google Health DeepMind, London, UK; Department of General, Visceral and Transplantation Surgery, Heidelberg University Hospital, Heidelberg, Germany; Translational Image-guided Oncology (TIO), Institute for AI in Medicine (IKIM), University Medicine Essen, Essen, Germany; Helmholtz AI, München, Germany; Lunit, Seoul, South Korea; German Cancer Research Center (DKFZ) Heidelberg, Division of Biostatistics, Germany; Centre for Biomedical Image Analysis and Faculty of Informatics, Masaryk University, Brno, Czech Republic; Cell Biology and Biophysics Unit, European Molecular Biology Laboratory (EMBL), Heidelberg, Germany; Department of Biomedical Informatics, Stony Brook University, Stony Brook, NY, USA; Electrical Engineering, Vanderbilt University, Nashville, TN, USA; Department of Pathology, Radboud University Medical Center, Nijmegen, The Netherlands; Department of Surgery, University Health Network, Philadelphia, PA, Canada; German Cancer Research Center (DKFZ) Heidelberg, Division of Medical Image Computing and HI Helmholtz Imaging, Germany; Pattern Analysis and Learning Group, Department of Radiation Oncology, Heidelberg University Hospital, Heidelberg, Germany; Physical Sciences, Sunnybrook Research Institute, Toronto, Canada; Department of Medical Biophysics, University of Toronto, Toronto, ON, Canada; Google, Mountain View, USA; School of Computer Science and Engineering, University of New South Wales, Sydney, Australia; Department of Quantitative Biomedicine, University of Zurich, Zurich, Switzerland; Julius Center for Health Sciences and Primary Care, UMC Utrecht, Utrecht University, Utrecht, The Netherlands; Information Systems Institute, University of Applied Sciences Western Switzerland (HES-SO), Sierre, Switzerland; Medical Faculty, University of Geneva, Geneva, Switzerland; MILA (Quebec Artificial Intelligence Institute), Montréal, Canada; Department of General, Visceral and Thoracic Surgery, University Medical Center Hamburg-Eppendorf, Hamburg, Germany; German Cancer Research Center (DKFZ) Heidelberg, Division of Medical Image Computing, Germany; Allen Institute for Cell Science, Seattle, WA, USA; Tissue Image Analytics Laboratory, Department of Computer Science, University of Warwick, Coventry, UK; ARTORG Center for Biomedical Engineering Research, University of Bern, Bern, Switzerland; Department of Radiation Oncology, University Hospital Bern, University of Bern, Bern, Switzerland; Simula Metropolitan Center for Digital Engineering, Oslo, Norway; UiT The Arctic University of Norway, Tromsø, Norway; NVIDIA GmbH, München, Germany; Institute for Computational Biomedicine, Heidelberg University, Heidelberg. Germany; Faculty of Medicine, Heidelberg University Hospital, Heidelberg, Germany; Informatics Institute, Faculty of Science, University of Amsterdam, Amsterdam, The Netherlands; Google Health, Google, CA, USA; National Institutes of Health Clinical Center, Bethesda, MD, USA; Institute of Information Systems Engineering, TU Wien, Vienna, Austria; Research Unit of Health Sciences and Technology, Faculty of Medicine, University of Oulu, Oulu, Finland; Neurocenter Oulu, Oulu University Hospital, Oulu, Finland; School of Engineering, The University of Edinburgh, Edinburgh, Scotland; Department of Development and Regeneration and EPI-centre, KU Leuven, Leuven, Belgium; Department of Biomedical Data Sciences, Leiden University Medical Center, Leiden, The Netherlands; Parietal project team, INRIA Saclay-Île de France, Palaiseau, France; National Institute of Allergy and Infectious Diseases, National Institutes of Health, Bethesda, MD, USA; German Cancer Research Center (DKFZ) Heidelberg, Interactive Machine Learning Group and HI Helmholtz Imaging, Germany; German Cancer Research Center (DKFZ) Heidelberg, Division of Intelligent Medical Systems and HI Helmholtz Imaging, Germany; Faculty of Mathematics and Computer Science and Medical Faculty, Heidelberg University, Heidelberg, Germany; National Center for Tumor Diseases (NCT), NCT Heidelberg, a partnership between DKFZ and University Medical Center Heidelberg, Germany

**Keywords:** Validation, Evaluation, Pitfalls, Metrics, Good Scientific Practice, Biomedical Image Processing, Challenges, Computer Vision, Classification, Segmentation, Instance Segmentation, Semantic Segmentation, Detection, Localization, Medical Imaging, Biological Imaging

## Abstract

Validation metrics are key for tracking scientific progress and bridging the current chasm between artificial intelligence (AI) research and its translation into practice. However, increasing evidence shows that particularly in image analysis, metrics are often chosen inadequately. While taking into account the individual strengths, weaknesses, and limitations of validation metrics is a critical prerequisite to making educated choices, the relevant knowledge is currently scattered and poorly accessible to individual researchers. Based on a multi-stage Delphi process conducted by a multidisciplinary expert consortium as well as extensive community feedback, the present work provides the first reliable and comprehensive common point of access to information on pitfalls related to validation metrics in image analysis. While focused on biomedical image analysis, the addressed pitfalls generalize across application domains and are categorized according to a newly created, domain-agnostic taxonomy. The work serves to enhance global comprehension of a key topic in image analysis validation.

Measuring performance and progress in any given field critically depends on the availability of meaningful outcome metrics. In a field such as athletics, this process is straightforward because the performance measurements (e.g., the time it takes an athlete to run a given distance) exactly reflect the underlying interest (e.g., which athlete runs a given distance the fastest?). In image analysis, the situation is much more complex. Depending on the underlying research question, vastly different aspects of an algorithm’s performance might be of interest (Fig. 1) and meaningful in determining its future practical, for example clinical, applicability. If the performance of an image analysis algorithm is not measured according to relevant validation metrics, no reliable statement can be made about the suitability of this algorithm in solving the proposed task, and the algorithm is unlikely to ever reach the stage of real-life application. Moreover, unsuitable algorithms could be wrongly regarded as the best-performing ones, sparking entirely futile resource investment and follow-up research while obscuring true scientific advancements. In determining new state-of-the-art methods and informing future directions, the use of validation metrics actively shapes the evolution of research. In summary, *validation metrics are the key for both measuring and informing scientific progress, as well as bridging the current chasm between image analysis research and its translation into practice*.

In image analysis, while for some applications it might, for instance, be sufficient to draw a box around the structure of interest (e.g., detecting individual mitotic cells or regions with apoptotic cell debrisa) and optionally associate that region with a classification (e.g., a mitotic vs an interphase cell), other applications (e.g., cell tracing for fluorescent signal quantification) could require determining the exact structure boundaries. The suitability of any individual validation metric thus depends crucially on the properties of the driving image analysis problem. As a result, numerous metrics have so far been proposed in the field of image processing. In our previous work, we analyzed all biomedical image analysis competitions conducted within a period of about 15 years [58]. We found a total of 97 different metrics reported in the field of biomedicine alone, each with its own individual strengths, weaknesses, and limitations, and hence varying degrees of suitability for meaningfully measuring algorithm performance on any given research problem. Such a vast range of options makes tracking all related information impossible for any individual researcher and consequently renders the process of metric selection error-prone. Thus, the frequent reliance on flawed, historically grown validation practices in current literature comes as no surprise. To make matters worse, there is currently no comprehensive resource that can provide an overview of the relevant definitions, (mathematical) properties, limitations, and pitfalls pertaining to a metric of interest. *While taking into account the individual properties and limitations of metrics is imperative for choosing adequate validation metrics, the required knowledge is thus largely inaccessible*.

As a result, numerous flaws and pitfalls are prevalent in image analysis validation, with researchers often being unaware of them due to a lack of knowledge of intricate metric properties and limitations. Accordingly, increasing evidence shows that metrics are often selected inadequately in image analysis (e.g., [34, 49, 84]). In the absence of a central information resource, it is common for researchers to resort to popular validation metrics, which, however, can be entirely unsuitable, for instance due to a mismatch of the metric’s inherent mathematical properties with the underlying research question and specifications of the data set at hand (see Fig. 1).

The present work addresses this important roadblock in image analysis research with a crowd-sourcing-based approach that involved both a Delphi process undergone by a multidisciplinary expert consortium as well as a social media campaign. It represents the *first comprehensive collection, visualization, and detailed discussion of pitfalls, drawbacks, and limitations regarding validation metrics commonly used in image analysis* Our work provides researchers with a *reliable, single point of access* to this critical information. Owing to the enormous complexity of the matter, the metric properties and pitfalls are discussed in the specific context of classification problems, i.e., image analysis problems that can be considered classification tasks at either the image, object, or pixel level. Specifically, these encompass the four problem categories of image-level classification, semantic segmentation, object detection, and instance segmentation. Our contribution includes a dedicated profile for each metric (SUPPL. NOTE 3) as well as the creation of a new common taxonomy that categorizes pitfalls in a domain-agnostic manner (Fig. 2). The taxonomy is depicted for individual metrics in provided tables (see Extended Data Tabs. 1-5) and enables researchers to quickly grasp whether using a certain metric comes with pitfalls in a given use case. While our work grew out of image analysis research and practice in the field of biomedicine, a field of high complexity and particularly high stakes due to its direct impact on human health, we believe the identified pitfalls to be transferable to other application areas of imaging research. It should be noted that this work focuses on identifying, categorizing, and illustrating metric pitfalls, while the sister publication of this work gives specific recommendations on which metrics to apply under which circumstances [59].

## Results

### Information on metric pitfalls is largely inaccessible

Researchers and algorithm developers seeking to validate image analysis algorithms frequently face the problem of choosing adequate validation metrics while at the same time navigating a range of potential pitfalls. Following common practice is often not the best option, as evidenced by a number of recent publications [34, 49, 58, 84]. Making an educated choice is notably complicated by the absence of any comprehensive databases or reviews covering the topic and thus the lack of a central resource for reliable information on validation metrics.

This lack of accessibility is considered by experts to be a major bottleneck in image analysis validation [58]. To illustrate this point, we searched the literature for available information on commonly used validation metrics. The search was conducted on the platform Google Scholar using search strings that combined different notations of the metric name, including synonyms and acronyms, with search terms indicating problems, such as “pitfall” or “limitation”. The mean and median number of hits for the metrics addressed in the present work were 159,329 and 22,100, respectively, and ranged between 49 for centerline Dice Similarity Coefficient (clDice) and 962,000 for Sensitivity. Moreover, despite valuable literature on individual relevant aspects (e.g., [14, 15, 36, 49, 80, 81, 84]), we did not find a common point of entry to metric-related pitfalls in image analysis in the form of a review paper or other credible source. We conclude that *the key knowledge required for making educated decisions and avoiding pitfalls related to the use of validation metrics is highly scattered and not accessible by individuals*.

### Historically grown practices are not always justified

To obtain an initial insight into current common practice regarding validation metrics, we prospectively captured the designs of challenges organized by the IEEE Society of the International Symposium of Biomedical Imaging (ISBI), the Medical Image Computing and Computer Assisted Interventions (MICCAI) Society and the Medical Imaging with Deep Learning (MIDL) foundation. The organizers of the respective competitions were asked to provide a rationale for the choice of metrics in their competition. An analysis of a total of 138 competitions conducted between 2018 and 2022 revealed that metrics are frequently (in 24% of the competitions) based on common practice in the community. We found, however, that common practices are often not well-justified, and poor practices may even be propagated from one generation to the next.

One remarkable example for this issue is the widespread adoption of an incorrect naming and inconsistent mathematical formulation of a metric proposed for cell instance segmentation. The term “mean Average Precision (mAP)” usually refers to one of the most common metrics in object detection (object-level classification) [57, 73]. Here, Precision denotes the Positive Predictive Value (PPV), which is “averaged” over varying thresholds on the predicted class scores of an object detection algorithm. The “mean” Average Precision (AP) is then obtained by taking the mean over classes [29, 73]. Despite the popularity of mAP, a widely known challenge on cell instance segmentation^1^ introduced a new “Mean Average Precision” in 2018. Although the task matches the task of the original “mean” AP, object detection, all terms in the newly proposed metric (mean, average, and precision) refer to entirely different concepts. For instance, the common definition of Precision from literature TP/(TP + FP) was altered to TP/(TP + FP + FN), where TP, FP, and FN refer to the cardinalities of the confusion matrix (i.e., the true/false positives/negatives). The latter formula actually defines the Intersection over Union (IoU) metric. Despite these problems, the terminology was adopted by subsequent influential works [42, 47, 77, 79], indicating widespread propagation and usage within the community.

### A multidisciplinary Delphi process reveals numerous pitfalls in biomedical image analysis validation

With the aim of creating a comprehensive, reliable collection and future point of access to biomedical image analysis metric definitions and limitations, we formed an international multidisciplinary consortium of 62 experts from various biomedical image analysis-related fields that engaged in a multi-stage Delphi process [9] for consensus building. The Delphi process comprised multiple surveys, developed by a coordinating team and filled out by the remaining members of the consortium. Based on the survey results, the list of pitfalls was iteratively refined by collecting pitfall sources, specific feedback and suggestions on pitfalls, and final agreement on which pitfalls to include and how to illustrate them. Further pitfalls were crowdsourced through the publication of a dynamic preprint of this work [73] as well as a social media campaign, both of which asked the scientific community for contributions. This approach allowed us to integrate distributed, cross-domain knowledge on metric-related pitfalls within a single resource. In total, the process revealed 37 distinct sources of pitfalls (see Fig. 2). Notably, these pitfall sources (e.g., class imbalances, uncertainties in the reference, or poor image resolution) can occur irrespective of a specific imaging modality or application. As a result, many pitfalls generalize across different problem categories in image processing (image-level classification, semantic segmentation, object detection, and instance segmentation), as well as imaging modalities and domains. A detailed discussion of all pitfalls can be found in SUPPL. NOTE 2.

### A common taxonomy enables domain-agnostic categorization of pitfalls

One of our key objectives was to facilitate information retrieval and provide structure within this vast topic. Specifically, we wanted to enable researchers to identify at a glance which metrics are affected by which types of pitfalls. To this end, we created a comprehensive taxonomy that categorizes the different pitfalls in a semantic fashion. The taxonomy was created in a domainagnostic manner to reflect the generalization of pitfalls across different imaging domains and modalities. An overview of the taxonomy is presented in Fig. 2, and the relations between the pitfall categories and individual metrics can be found in Extended Data Tabs. 1-5. We distinguish the following three main categories:

#### [P1] Pitfalls related to the inadequate choice of the problem category

**Fig. 1:**
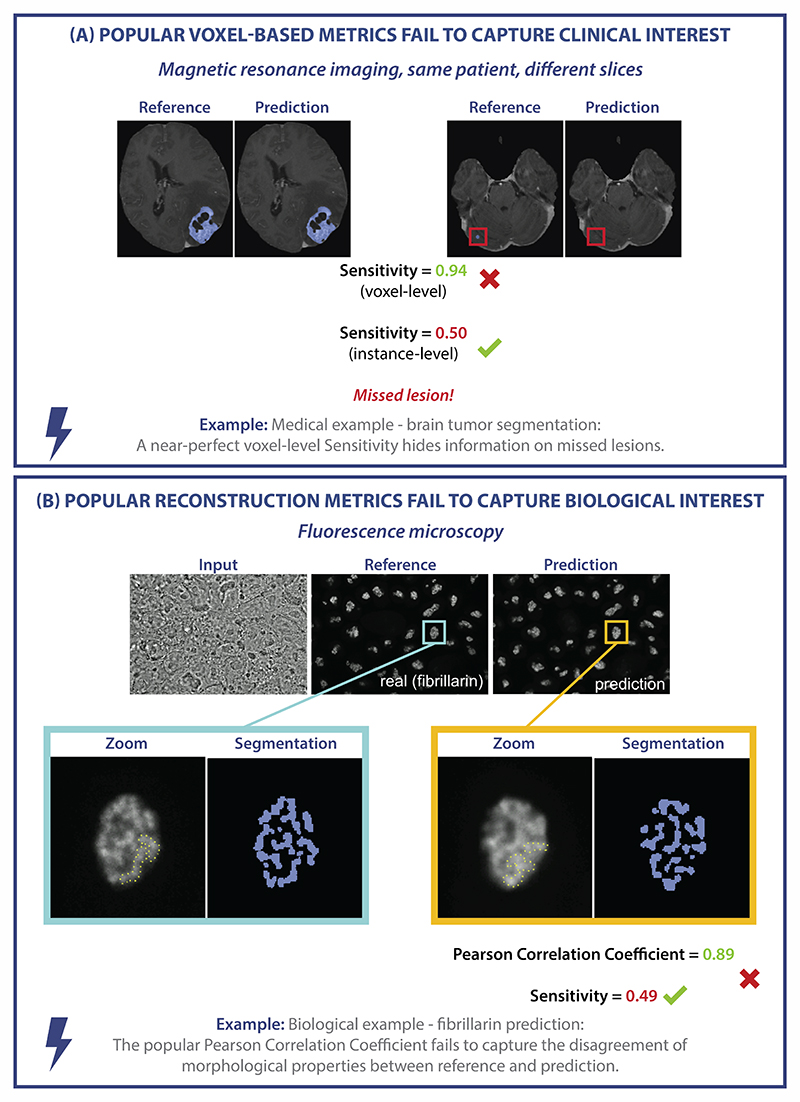
Examples of metric-related pitfalls in image analysis validation. (A) Medical image analysis example: Voxel-based metrics are not appropriate for detection problems. Measuring the voxel-level performance of a prediction yields a near-perfect Sensitivity. However, the Sensitivity at the instance level reveals that lesions are actually missed by the algorithm. (B) Biological image analysis example: The task of predicting fibrillarin in the dense fibrillary component of the nucleolus should be phrased as a segmentation task, for which segmentation metrics reveal the low quality of the prediction. Phrasing the task as image reconstruction instead and validating it using metrics such as the Person Correlation Coefficient yields misleadingly high metric scores [12, 68, 74, 88, 88].

**Fig. 2:**
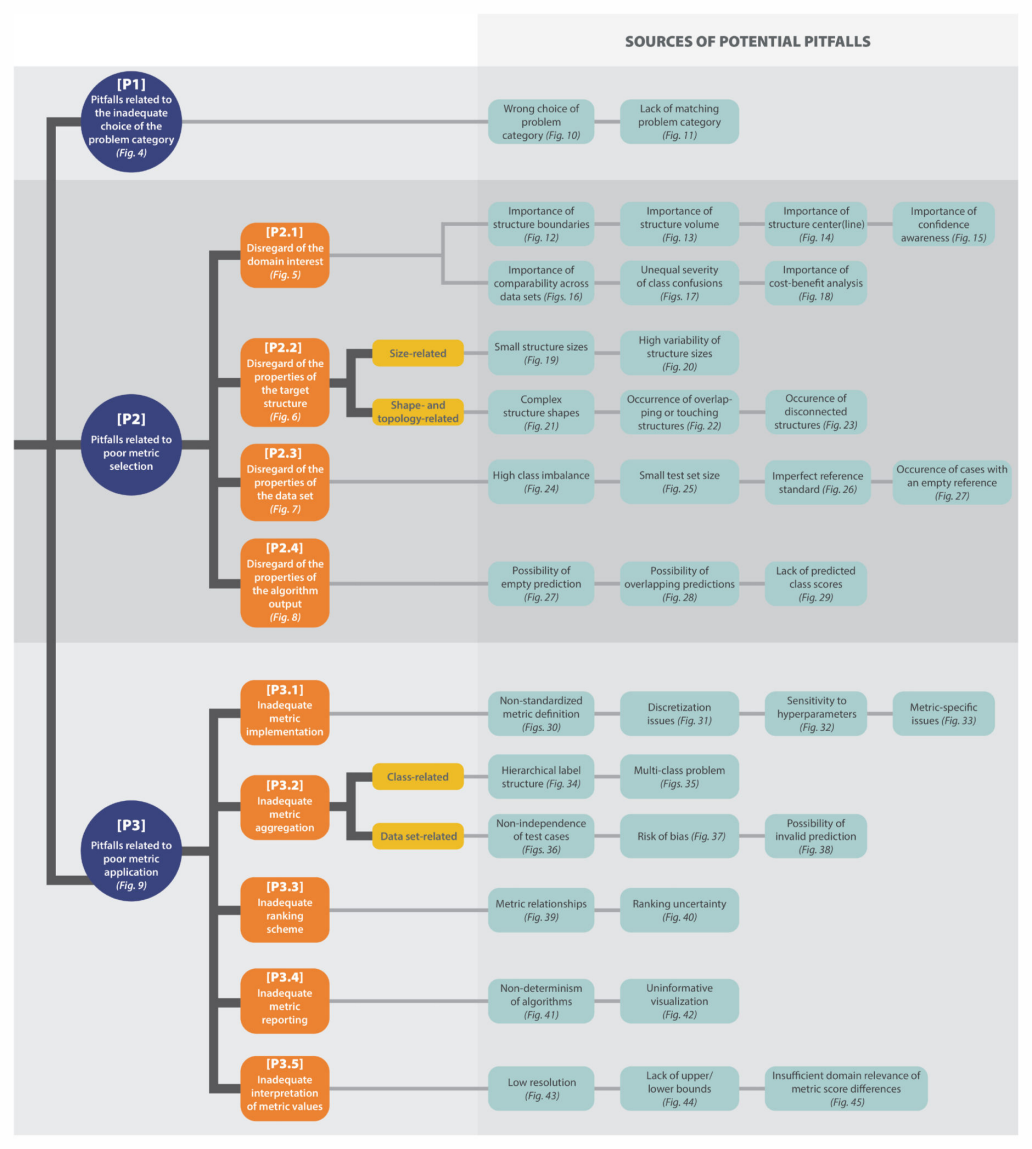
Overview of the taxonomy for metric-related pitfalls. Pitfalls can be grouped into three main categories: [P1] Pitfalls related to the inadequate choice of the problem category, [P2] pitfalls related to poor metric selection, and [P3] pitfalls related to poor metric application. [P2] and [P3] are further split into subcategories. For all categories, pitfall sources are presented (green), with references to corresponding illustrations of representative examples. Note that the order in which the pitfall sources are presented does not correlate with importance.

A common pitfall lies in the use of metrics for a problem category they are not suited for because they fail to fulfill crucial requirements of that problem category, and hence do not reflect the domain interest (Fig. 1). For instance, popular voxel-based metrics, such as the Dice Similarity Coefficient (DSC) or Sensitivity, are widely used in image analysis problems, although they do not fulfill the critical requirement of detecting all objects in a data set. In a cancer monitoring application they fail to measure instance progress, i.e., the potential increase in number of lesions (Fig. 1), which can have serious consequences for the patient. For some problems, there may even be a lack of matching problem category (Fig. SN 2.4), rendering common metrics inadequate. We present further examples of pitfalls in this category in Suppl. Note 2.1.

#### [P2] Pitfalls related to poor metric selection

Pitfalls of this category occur when a validation metric is selected while disregarding specific properties of the given research problem or method used that make this metric unsuitable in the particular context. [P2] can be further divided into the following four subcategories:

##### [P2.1] Disregard of the domain interest

**Fig. 4:**
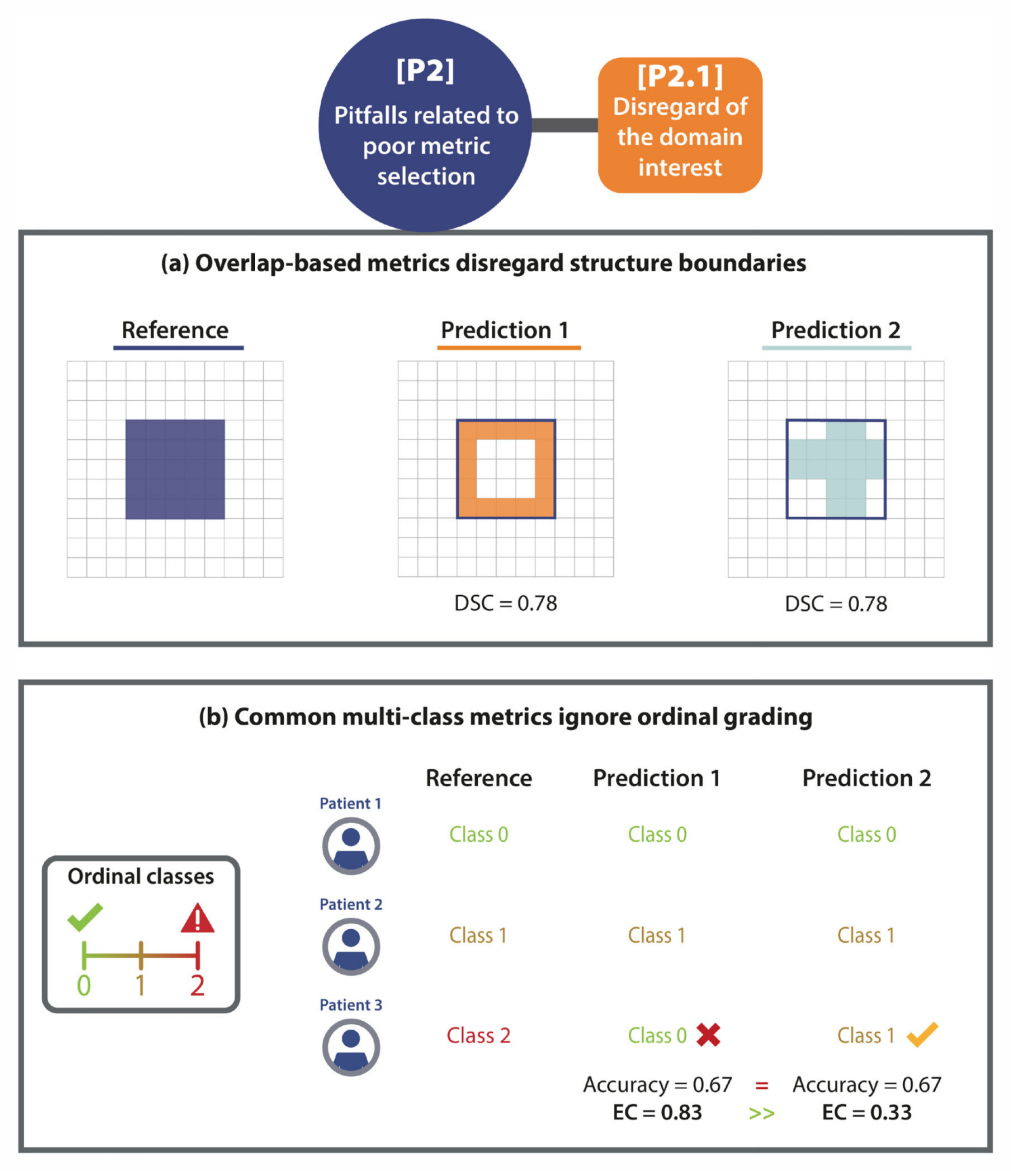
[P2.1] Disregard of the domain interest. **(a) Importance of structure boundaries**. The predictions of two algorithms (*Prediction 1/2*) capture the boundary of the given structure substantially differently, but lead to the exact same Dice Similarity Coefficient (DSC), due to its boundary unawareness. This pitfall is also relevant for other overlap-based metrics such as centerline Dice Similarity Coefficient (clDice), pixel-level F_*β*_ Score, and Intersection over Union (IoU), as well as localization criteria such as Box/Approx/Mask IoU, Center Distance, Mask IoU > 0, Point inside Mask/Box/Approx, and Intersection over Reference (IoR). **(b) Unequal severity of class confusions**. When predicting the severity of a disease for three patients in an ordinal classification problem, *Prediction 1* assumes a much lower severity for *Patient 3* than actually observed. This critical issue is overlooked by common metrics (here: Accuracy), which measure no difference to *Prediction 2*, which assesses the severity much better. Metrics with pre-defined weights (here: Expected Cost (EC)) correctly penalize *Prediction 1* much more than *Prediction 2*. This pitfall is also relevant for other counting metrics, such as Balanced Accuracy (BA), F_*β*_ Score, Positive Likelihood Ratio (LR+), Matthews Correlation Coefficient (MCC), Net Benefit (NB), Negative Predictive Value (NPV), Positive Predictive Value (PPV), Sensitivity, and Specificity.

Commonly, several requirements arise from the domain interest of the underlying research problem that may clash with particular metric limitations. For example, if there is particular interest in the structure boundaries, it is important to know that overlap-based metrics such as the DSC do not take the correctness of an object’s boundaries into account, as shown in Fig. 4(a). Similar issues may arise if the structure volume (Fig. SN 2.6) or center(line) (Fig. SN 2.7) are of particular interest. Other domain interest-related properties may include an unequal severity of class confusions. This may be important in an ordinal grading use case, in which the severity of a disease is categorized by different scores. Predicting a low severity for a patient that actually suffers from a severe disease should be substantially penalized. Common classification metrics do not fulfill this requirement. An example is provided in Fig. 4(b). On pixel level, this property relates to an unequal severity of over- vs. undersegmentation. In applications such as radiotherapy, it may be highly relevant whether an algorithm tends to over- or undersegment the target structure. Common overlap-based metrics, however, do not represent over- and undersegmentation equally [96]. Further pitfalls may occur if confidence awareness (Fig. SN 2.8), comparability across data sets (Fig. SN 2.9), or a cost-benefit analysis (Fig. SN 2.11) are of particular importance, as illustrated in Suppl. Note 2.2.1.

##### [P2.2] Disregard of the properties of the target structures

**Extended Data Fig. 1:**
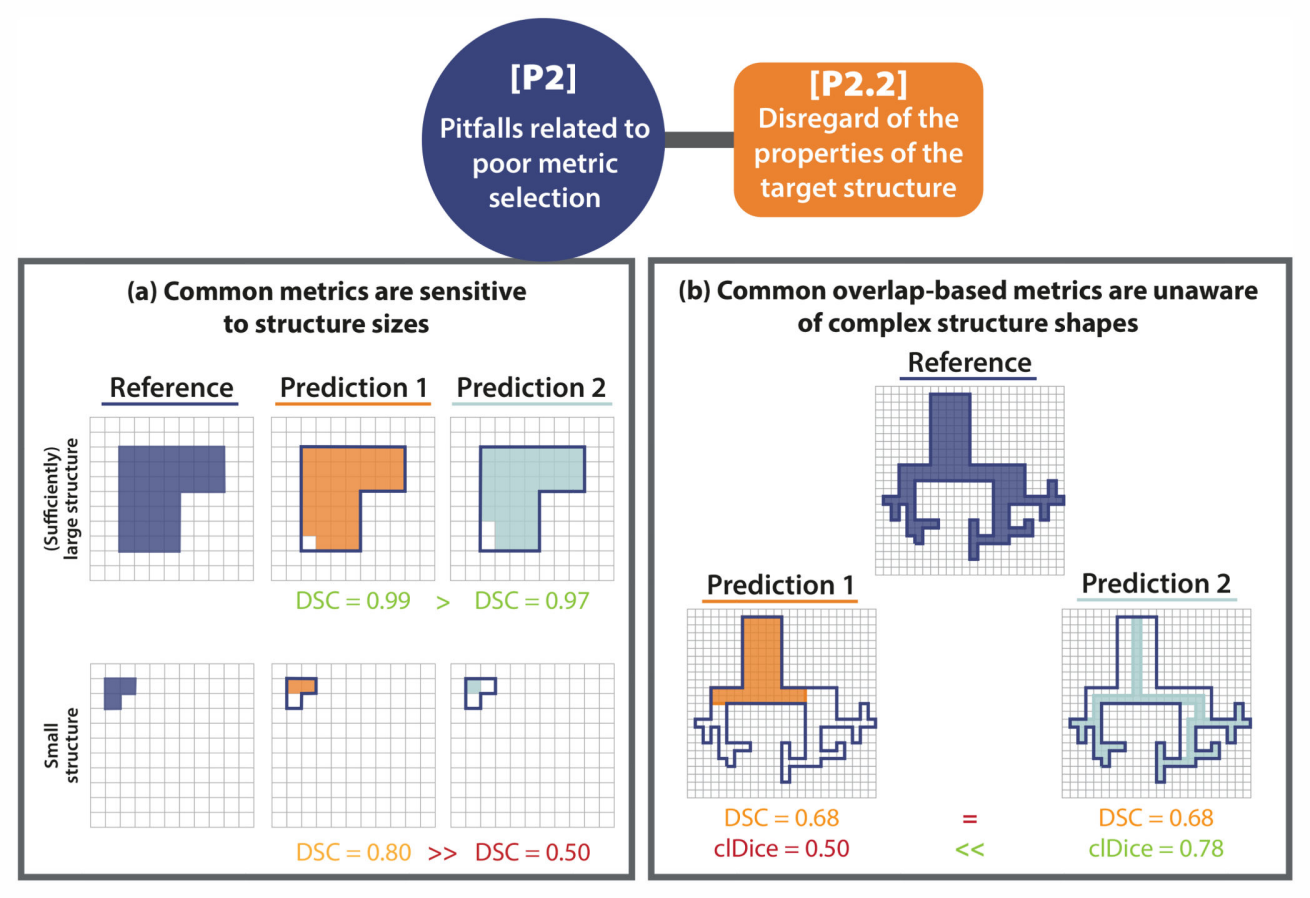
[P2.2] Disregard of the properties of the target structures. **(a) Small structure sizes**. The predictions of two algorithms (*Prediction 1/2*) differ in only a single pixel. In the case of the small structure (bottom row), this has a substantial effect on the corresponding Dice Similarity Coefficient (DSC) metric value (similar for the Intersection over Union (IoU)). This pitfall is also relevant for other overlap-based metrics such as the centerline Dice Similarity Coefficient (clDice), and localization criteria such as Box/Approx/Mask IoU and Intersection over Reference (IoR). **(b) Complex structure shapes**. Common overlap-based metrics (here: DSC) are unaware of complex structure shapes and treat *Predictions 1* and *2* equally. The clDice uncovers the fact that *Prediction 1* misses the fine-granular branches of the reference and favors *Prediction 2*, which focuses on the center line of the object. This pitfall is also relevant for other overlap-based such as metrics IoU and pixel-level F_*β*_ Score as well as localization criteria such as Box/Approx/Mask IoU, Center Distance, Mask IoU > 0, Point inside Mask/Box/Approx, and IoR.

For problems that require capturing local properties (object detection, semantic or instance segmentation), the properties of the target structures to be localized and/or segmented may have important implications for the choice of metrics. Here, we distinguish between *size-related* and *shape- and topology-related* pitfalls. Common metrics, for example, are sensitive to structure sizes, such that single-pixel differences may hugely impact the metric scores, as shown in Extended Data Fig. 1(a). Shape- and topology-related pitfalls may relate to the fact that common metrics disregard complex shapes (Extended Data Fig. 1(b)) or that bounding boxes do not capture the disconnectedness of structures (Fig. SN 2.16). A high variability of structure sizes (Fig. SN 2.13) and overlapping or touching structures (Fig. SN 2.15) may also influence metric values. We present further examples of [P2.2] pitfalls in Suppl. Note 2.2.2.

##### [P2.3] Disregard of the properties of the data set

**Extended Data Fig. 2:**
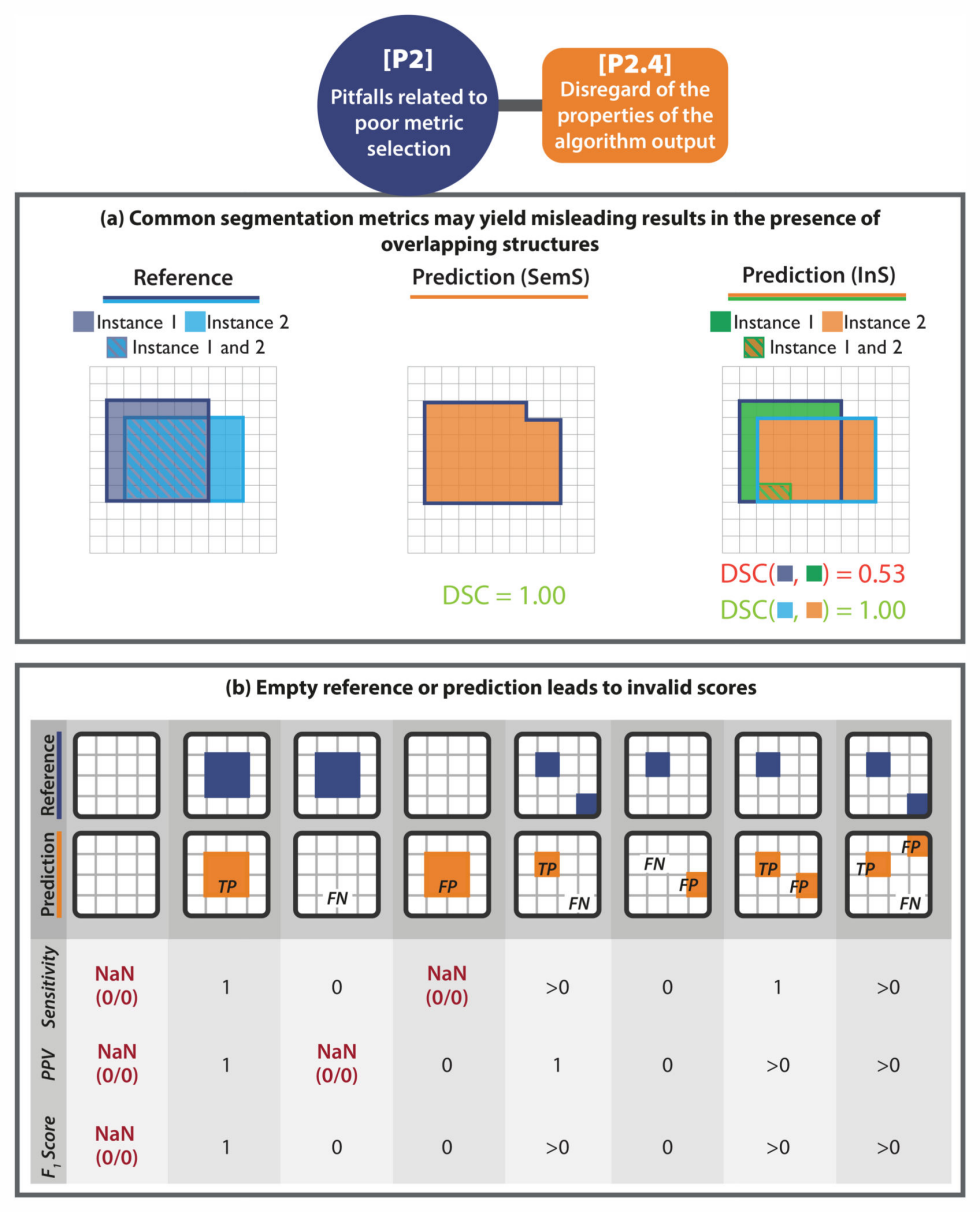
[P2.4] Disregard of the properties of the algorithm output. **(a) Possibility of overlapping predictions**. If multiple structures of the same type can be seen within the same image (here: reference objects *R1* and *R2*), it is generally advisable to phrase the problem as instance segmentation (InS; right) rather than semantic segmentation (SemS; left). This way, issues with boundary-based metrics resulting from comparing a given structure boundary to the boundary of the wrong instance in the reference can be avoided. In the provided example, the distance of the red boundary pixel to the reference, as measured by a boundary-based metric in SemS problems, would be zero, because different instances of the same structure cannot be distinguished. This problem is overcome by phrasing the problem as InS. In this case, (only) the boundary of the matched instance (here: R2) is considered for distance computation. **(b) Possibility of empty prediction or reference**. Each column represents a potential scenario for per-image validation of objects, categorized by whether True Positives (TPs), False Negatives (FNs), and False Positives (FPs) are present (n > 0) or not (n = 0) after matching/assignment. The sketches on the top showcase each scenario when setting “n > 0” to “n = 1”. For each scenario, Sensitivity, Positive Predictive Value (PPV), and the F_1_ Score are calculated. Some scenarios yield undefined values (Not a Number (NaN)).

**Fig. 5:**
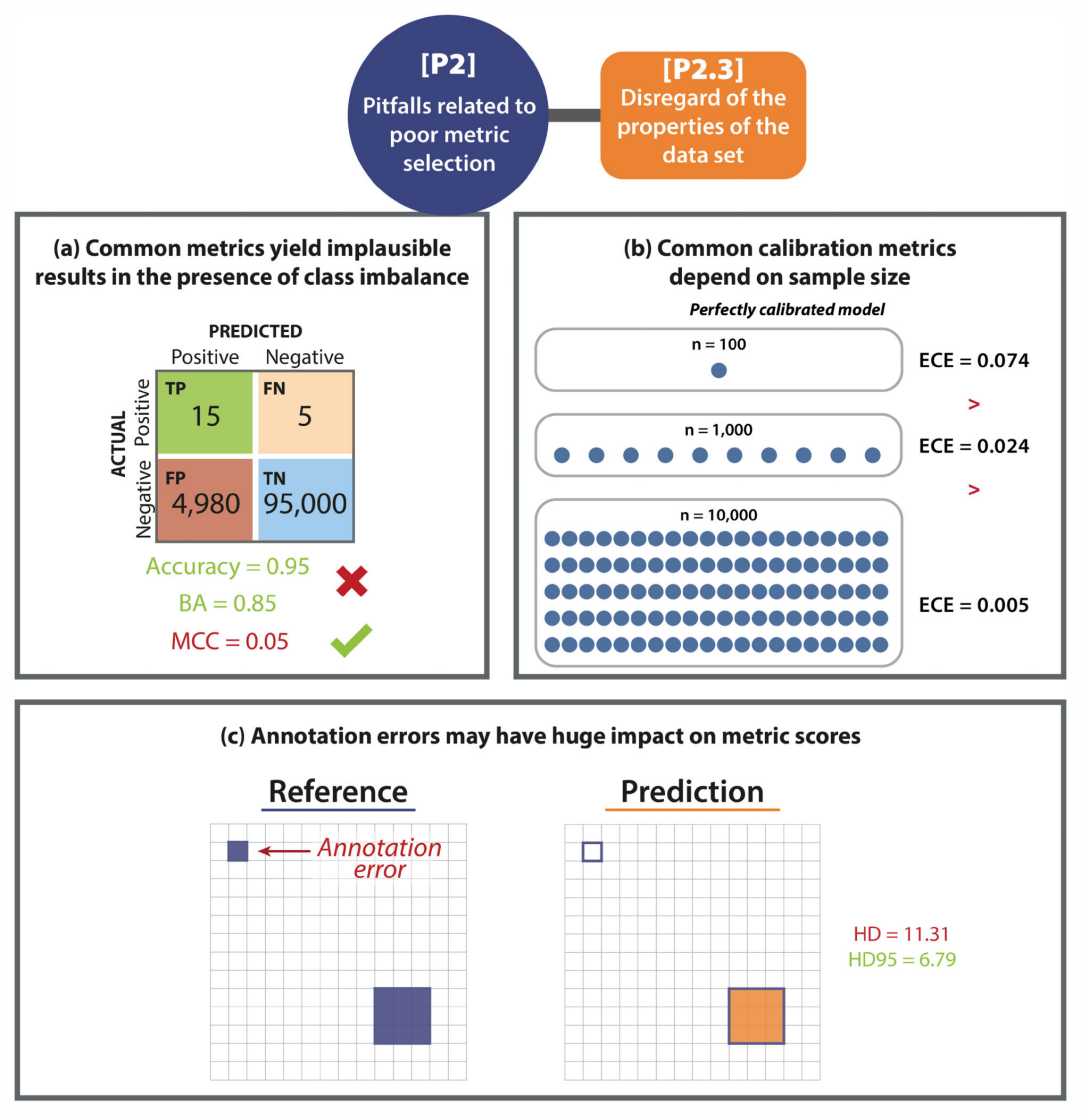
[P2.3] Disregard of the properties of the data set. **(a) High class imbalance**. In the case of underrepresented classes, common metrics may yield misleading values. In the given example, Accuracy and Balanced Accuracy (BA) have a high score despite the high amount of False Positive (FP) samples. The class imbalance is only uncovered by metrics considering predictive values (here: Matthews Correlation Coefficient (MCC)). This pitfall is also relevant for other counting and multi-threshold metrics such as Area under the Receiver Operating Characteristic Curve (AUROC), Expected Cost (EC) (depending on the chosen costs), Positive Likelihood Ratio (LR+), Net Benefit (NB), Sensitivity, Specificity, and Weighted Cohen’s Kappa (WCK). **(b) Small test set size**. The values of the Expected Calibration Error (ECE) depend on the sample size. Even for a simulated perfectly calibrated model, the ECE will be substantially greater than zero for small sample sizes [37]. **(c) Imperfect reference standard**. A single erroneously annotated pixel may lead to a large decrease in performance, especially in the case of the Hausdorff Distance (HD) when applied to small structures. The Hausdorff Distance 95th Percentile (HD95), on the other hand, was designed to deal with spatial outliers. This pitfall is also relevant for localization criteria such as Box/Approx Intersection over Union (IoU) and Point inside Box/Approx. Further abbreviations: True Positive (TP), False Negative (FN), True Negative (TN).

Various properties of the data set such as class imbalances (Fig. 5(a)), small sample sizes (Fig. 5(b)), or the quality of the reference annotations, may directly affect metric values. Common metrics such as the Balanced Accuracy (BA), for instance, may yield a very high score for a model that predicts many False Positive (FP) samples in an imbalanced setting (see Fig. 5(a)). When only small test data sets are used, common calibration metrics (which are typically biased estimators) either underestimate or overestimate the true calibration error of a model (Fig. 5(b)) [37]. On the other hand, metric values may be impacted by reference annotations (Fig. SN 2.19). Spatial outliers in the reference may have a huge impact on distance-based metrics such as the Hausdorff Distance (HD) (Fig. 5(c)). Additional pitfalls may arise from the occurrence of cases with an empty reference (Extended Data Fig. 2(b)), causing division by zero errors. We present further examples of [P2.3] pitfalls in Suppl. Note 2.2.3.

##### [P2.4] Disregard of the properties of the algorithm output

Reference-based metrics compare the algorithm output to a reference annotation to compute a metric score. Thus, the content and format of the prediction are of high importance when considering metric choice. Overlapping predictions in segmentation problems, for instance, may return misleading results. In Extended Data Fig. 2(a), the predictions only overlap to a certain extent, not representing that the reference instances actually overlap substantially. This is not detected by common metrics. Another example are empty predictions that may cause division by zero errors in metric calculations, as illustrated in Extended Data Fig. 2(b), or the lack of predicted class scores (Fig. SN 2.22). We present further examples of [P2.4] pitfalls in Suppl. Note 2.2.3.

#### [P3] Pitfalls related to poor metric application

**Fig. 6:**
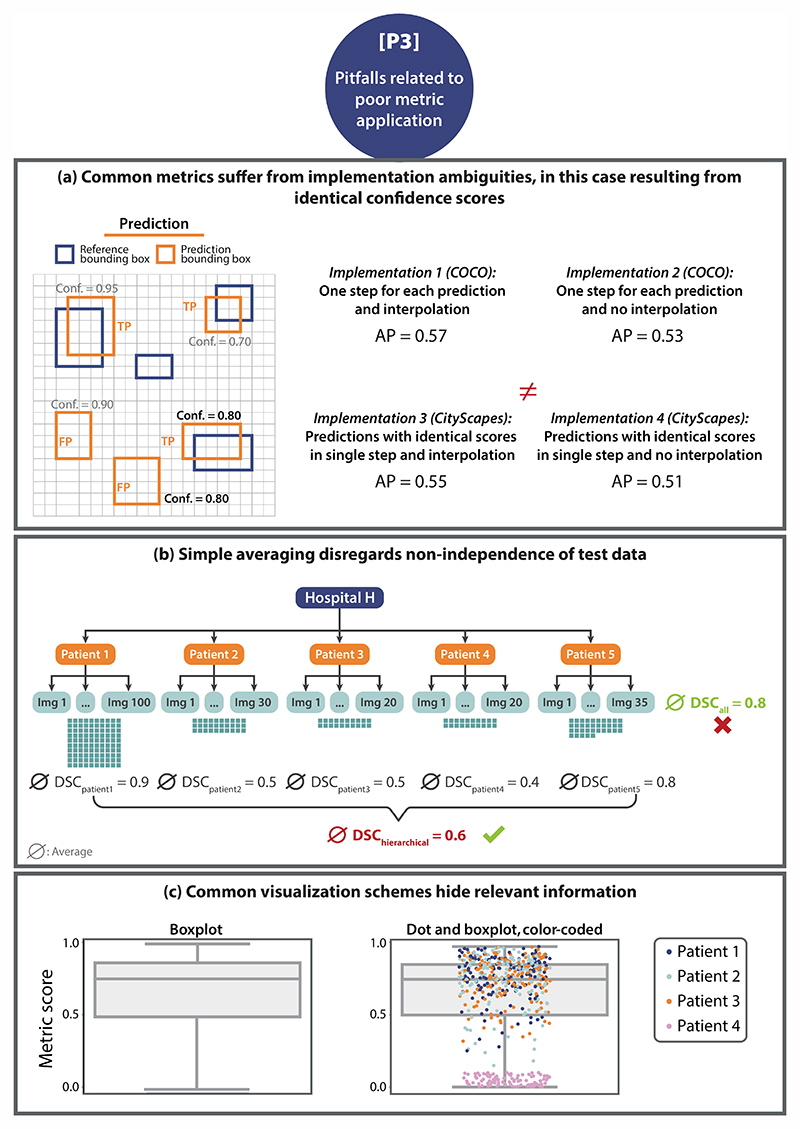
[P3] Pitfalls related to poor metric application. **(a) Non-standardized metric implementation**. In the case of the Average Precision (AP) metric and the construction of the Precision-Recall (PR)-curve, the strategy of how identical scores (here: confidence score of 0.80 is present twice) are treated has a substantial impact on the metric scores. Microsoft Common Objects in Context (COCO) [57] and CityScapes [18] are used as examples. **(b) Non-independence of test cases**. The number of images taken from *Patient 1* is much higher compared to that acquired from *Patients 2-5*. Averaging over all Dice Similarity Coefficient (DSC) values, denoted by ∅, results in a high averaged score. Aggregating metric values per patient reveals much higher scores for *Patient 1* compared to the others, which would have been hidden by simple aggregation. **(c) Uninformative visualization**. A single box plot (left) does not give sufficient information about the raw metric value distribution. Adding the raw metric values as jittered dots on top (right) adds important information (here: on clusters). In the case of non-independent validation data, color/shape-coding helps reveal data clusters.

Once selected, the metrics need to be applied to an image or an entire data set. This step is not straightforward and comes with several pitfalls. For instance, when aggregating metric values over multiple images or patients, a common mistake is to ignore the hierarchical data structure, such as data from several hospitals or a varied number of images per patient. We present three examples of [P3] pitfalls in Fig. 6; for more pitfalls in this category, please refer to Suppl. Note 2.3. [P3] can further be divided into five subcategories that are presented in the following paragraphs.

##### [P3.1] Inadequate metric implementation

Metric implementation is, unfortunately, not standardized. As shown by [35], different researchers typically employ various different implementations for the same metric, which may yield a substantial variation in the metric scores. While some metrics are straightforward to implement, others require more advanced techniques and offer different possibilities. In the following, we provide some examples for inadequate metric implementation:

- The method of how identical confidence scores are handled in the computation of the AP metric may lead to substantial differences in the metric scores. Microsoft Common Objects in Context (COCO) [57], for instance, processes each prediction individually, while CityScapes [18] processes all predictions with the same score in one joint step. Fig. 6(a) provides an example with two predictions having the same confidence score, in which the final metric scores differ depending on the chosen handling strategy for identical confidence scores. Similar issues may arise with other curve-based metrics, such as AUROC, AP, or Free-Response Receiver Operating Characteristic (FROC) scores (see e.g., [63]).
- Metric implementation may be subject to discretization issues such as the chosen discretization of continuous variables, which may cause differences in the metric scores, as exemplary illustrated in Fig. SN 2.24.
- For metrics assessing structure boundaries, such as the Average Symmetric Surface Distance (ASSD), the exact boundary extraction method is not standardized. Thus, for example, the boundary extraction method implemented by the Liver Tumor Segmentation (LiTS) challenge [7] and that implemented by Google DeepMind^2^ may produce different metric scores for the ASSD. This is especially critical for metrics that are sensitive to small contour changes, such as the HD.
- Suboptimal choices of hyperparameters may also lead to metric scores that do not reflect the domain interest. For example, the choice of a threshold on a localization criterion (see Fig. SN 2.25) or the chosen hyperparameter for the F_*β*_ Score will heavily influence the subsequent metric scores [83].

More [P3.1] pitfalls can be found in Suppl. Note 2.3.1.

##### [P3.2] Inadequate metric aggregation

A common pitfall with respect to metric application is to simply aggregate metric values over the entire data set and/or all classes. As detailed in Fig. 6(b) and Suppl. Note 2.3.2, important information may get lost in this process, and metric results can be misleading. For example, the popular TorchMetrics framework calculates the DSC metric by default as a global average over all pixels in the data set without considering their image or class of origin^3^. Such a calculation eliminates the possibility of interpreting the final metric score with respect to individual images and classes. For example, errors in small structures may be suppressed by correctly segmented larger structures in other images (see e.g., Fig. SN 2.28). An adequate aggregation scheme is also crucial for handling hierarchical class structure (Fig. SN 2.29), missing values (Fig. SN 2.31), and potential biases (Fig. SN 2.30) of the algorithm. Further [P3.2] pitfalls are shown in Suppl. Note 2.3.2.

##### [P3.3] Inadequate ranking scheme

Rankings are often created to compare algorithm performances. In this context, several pitfalls pertain to either metric relationships or ranking uncertainty. For example, to assess different properties of an algorithm, it is advisable to select multiple metrics and determine their values. However, the chosen metrics should assess complementary properties and should not be mathematically related. For example, the DSC and IoU are closely related, so using both in combination would not provide any additional information over using either of them individually (Fig. SN 2.32). Note in this context that unawareness of metric synonyms can equally mislead. Metrics can be known under different names; for instance, Sensitivity and Recall refer to the same mathematical formula. Despite this fact potentially appearing trivial, an analysis of 138 biomedical image analysis challenges [59] found three challenges that unknowingly used two versions of the same metric to calculate their rankings. Moreover, rankings themselves may be unstable (Fig. SN 2.33). [58] and [94] demonstrated that rankings are highly sensitive to altering the metric aggregation operators, the underlying data set, or the general ranking method. Thus, if the robustness of rankings is disregarded, the winning algorithm may be identified by chance rather than true superiority.

##### [P3.4] Inadequate metric reporting

A thorough reporting of metric values and aggregates is important both in terms of transparency and interpretability. However, several pitfalls are to be avoided in this regard. Notably, different types of visualization may vary substantially in terms of interpretability, as shown in Figs 6(c). For example, while a box plot provides basic information, it does not depict the distribution of metric values. This may conceal important information, such as specific images on which an algorithm performed poorly. Other pitfalls in this category relate to the non-determinism of algorithms, which introduces a natural variability to the results of a neural network, even with fixed seeds (Fig. SN 2.34). This issue is aggravated by inadequate reporting, for instance, reporting solely the results from the best run instead of proper cross-validation and reporting of the variability across different runs. Generally, shortcomings in reporting, such as providing no standard deviation or confidence intervals in the presented results, are common. Concrete examples of [P3.4] pitfalls can be found in Suppl. Note 2.3.4.

##### [P3.5] Inadequate interpretation of metric values

Interpreting metric scores and aggregates is an important step for the analysis of algorithm performances. However, several pitfalls can arise from the interpretation. In rankings, for example, minor differences in metric scores may not be relevant from an application perspective but may still yield better ranks (Fig. SN 2.38). Furthermore, some metrics do not have upper or lower bounds, or the theoretical bounds may not be achievable in practice, rendering interpretation difficult (Fig. SN 2.37). More information on interpretation-based pitfalls can be found in Suppl. Note 2.3.5.

### The first illustrated common access point to metric definitions and pitfalls

To underline the importance of a common access point to metric pitfalls, we conducted a search for individual metric-related pitfalls on the platforms Google Scholar and Google, with the purpose of determining how many of the pitfalls identified through our work could be located in existing resources. We were only able to locate a portion of the pitfalls identified by our approach in existing research literature (68%) or online resources such as blog posts (11%; 8% were found in both). Only 27% of the located pitfalls were presented visually.

Our work now provides this key resource in a highly structured and easily understandable form. SUPPL. NOTE 2, contains a dedicated illustration for each of the pitfalls discussed, thus facilitating reader comprehension and making the information accessible to everyone regardless of their level of expertise. A further core contribution of our work are the metric profiles presented in SUPPL. NOTE 2, which, for each metric, summarize the most important information deemed of particular relevance by the *Metrics Reloaded* consortium of the sister work to this publication [59]. The profiles provide the reader with a compact, at-a-glance overview of each metric and an enumeration of the limitations and pitfalls identified in the Delphi process conducted for this work.

## Discussion

Flaws in the validation of biomedical image analysis algorithms significantly impede the translation of methods into (clinical) practice and undermine the assessment of scientific progress in the field [56]. They are frequently caused by poor choices due to disregarding the specific properties and limitations of individual validation metrics. The present work represents the first comprehensive collection of pitfalls and limitations to be considered when using validation metrics in image-level classification, semantic segmentation, instance segmentation, and object detection tasks. Our work enables researchers to gain a deep understanding of and familiarity with both the overall topic and individual metrics by providing a common access point to previously largely scattered and inaccessible information — key knowledge they can resort to when conducting validation of image analysis algorithms. This way, our work aims to disrupt the current common practice of choosing metrics based on their popularity rather than their suitability to the underlying research problem. This practice, which, for instance, often manifests itself in the unreflected and inadequate use of the DSC, is concerningly prevalent even among prestigious, high-quality biomedical image analysis competitions (challenges) [19, 34, 44, 49, 50, 58, 60, 84]. The educational aspect of our work is complemented by dedicated ‘metric profiles’ which detail the definitions and properties of all metrics discussed. Notably, our work pioneers the examination of artificial intelligence (AI) validation pitfalls in the biomedical domain, a domain in which they are arguably more critical than in many others as flaws in biomedical algorithm validation can directly affect patient wellbeing and safety.

We posited that shortcomings in current common practice are marked by the low accessibility of information on the pitfalls and limitations of commonly used validation metrics. A literature search conducted from the point of view of a researcher seeking information on individual metrics confirmed that the number of search results far exceeds any amount that could be overseen within reasonable time and effort, as well as the lack of a common point of entry to reliable metric information. Even when knowing the specific pitfalls and related keywords uncovered by our consortium, only a fraction of those pitfalls could be found in existing literature, indicating the novelty and added value of our work.

For transparency, several constraints regarding our literature search must be noted. First, it must be acknowledged that the remarkably high search result numbers inevitably include duplicates of papers (e.g., the same work in a conference paper and on arXiv) as well as results that are out of scope (e.g., [11], [26]), in the cited examples for instance due to a metric acronym (AUC) simultaneously being an acronym for another entity (a trinucleotide) in a different domain, or the word “sensitivity” being used in its common, non-metric meaning. Moreover, common words used to describe pitfalls such as “problem” or “issue” are by nature present in many publications discussing any kind of research, rendering them unusable for a dedicated search, which could, in turn, account for missing publications that do discuss pitfalls in these terms. Similarly, when searching for specific pitfalls, many of the returned results containing the appropriate keywords did not actually refer to metrics or algorithm validation but to other parts of a model or biomedical problem (e.g., the need for stratification is commonly discussed with regard to the design of clinical studies but not with regard to their validation). Character limits in the Google Scholar search bar further complicate or prevent the use of comprehensive search strings. Finally, it is both possible and probable that our literature search did not retrieve all publications or non-peer-reviewed online resources that mention a particular pitfall, since even extensive search strings might not cover the particular words used for a pitfall description.

None of these observations, however, detracts from our hypothesis. In fact, all of the above observations reinforce our finding that, for any individual researcher, retrieving information on metrics of interest is difficult to impossible. In many cases, finding information on pitfalls only appears feasible if the specific pitfall and its related keywords are exactly known, which, of course, is not the situation most researchers realistically find themselves in. Overall accessibility of such vital information, therefore, currently leaves much to be desired.

Compiling this information through a multi-stage Delphi process allowed us to leverage distributed knowledge from experts across different biomedical imaging domains and thus ensure that the resulting illustrated collection of metric pitfalls and limitations is both comprehensive and of maximum practical relevance. Continued proximity of our work to issues occurring in practical application was achieved through sharing the first results of this process as a dynamic preprint [72] with dedicated calls for feedback, as well as crowdsourcing further suggestions on social media.

Although their severity and practical consequences might differ between applications, we found that the pitfalls generalize across different imaging modalities and application domains. By categorizing them solely according to their underlying sources, we were able to create an overarching taxonomy that goes beyond domain-specific concerns and thus enjoys broad applicability. Given the large number of identified pitfalls, our taxonomy crucially establishes structure in the topic. Moreover, by relating types of pitfalls to the respective metrics they apply to and illustrating them, it enables researchers to gain a deeper, systemic understanding of the causes of metric failure.

Our complementary *Metrics Reloaded* recommendation framework, which guides researchers towards the selection of appropriate validation metrics for their specific tasks and is introduced in a sister publication to this work [59], shares the same principle of domain independence. Its recommendations are based on the creation of a ‘problem fingerprint’ that abstracts from specific domain knowledge and, informed by the pitfalls discussed here, captures all properties relevant to metric selection for a specific biomedical problem. In this sister publication, we present recommendations to avoid the pitfalls presented in this work. Importantly, the finding that pitfalls generalize and can be categorized in a domain-independent manner opens up avenues for future expansion of our work to other fields of ML-based imaging, such as general computer vision (see below), thus freeing it from its major constraint of exclusively focusing on biomedical problems.

It is worth mentioning that we only examined pitfalls related to the tasks of image-level classification, semantic segmentation, instance segmentation, and object detection, as these can all be considered classification tasks at different levels (image/object/pixel) and hence share similarities in their validation. While including a wider range of biomedical problems not considered classification tasks, such as regression or registration, would have gone beyond the scope of the present work, we envision this expansion in future work. Moreover, our work focused on pitfalls related to reference-based metrics. Including pitfalls pertaining to non-reference-based metrics, such as metrics that assess speed, memory consumption, or carbon footprint, could be a future direction to take. Finally, while we aspired to be as comprehensive as possible in our compilation, we cannot exclude that there are further pitfalls to be taken into account that the consortium and the participating community have so far failed to recognize. Should this be the case, our dynamic *Metrics Reloaded* online platform, which is currently under development and will continuously be updated after release, will allow us to easily and transparently append missed pitfalls. This way, our work can remain a reliable point of access, reflecting the state of the art at any given moment in the future. In this context, we note that we explicitly welcome feedback and further suggestions from the readership of *Nature Methods*.

The expert consortium was primarily compiled in a way to cover the required expertise from various fields but also consisted of researchers of different countries, (academic) ages, roles, and backgrounds (details can be found in the Suppl. Methods). It mainly focused on biomedical applications. The pitfalls presented here are therefore of the highest relevance for biological and clinical use cases. Their clear generalization across different biomedical imaging domains, however, indicates broader generalizability to fields such as general computer vision. Future work could thus see a major expansion of our scope to AI validation well beyond biomedical research. Regardless of this possibility, we strongly believe that by raising awareness of metric-related pitfalls, our work will kick off a necessary scientific debate. Specifically, we see its potential in inducing the scientific communities in other areas of AI research to follow suit and investigate pitfalls and common practices impairing progress in their specific domains.

In conclusion, our work presents the first comprehensive and illustrated access point to information on validation metric properties and their pitfalls. We envision it to not only impact the quality of algorithm validation in biomedical imaging and ultimately catalyze faster translation into practice, but to raise awareness on common issues and call into question flawed AI validation practice far beyond the boundaries of the field.

## Extended Data

**Extended Data Tab. 1:** Overview of pitfall sources for *image-level classification metrics* ((a): counting metrics, (b): multi-threshold metrics) related to poor metric selection [P2]. A warning sign indicates a potential pitfall for the metric in the corresponding column, in case the property represented by the respective row holds true. Comprehensive illustrations of pitfalls are available in SUPPL. NOTE 2. A comprehensive list of pitfalls is provided separately for each metrics in the metrics cheat sheets (SUPPL. NOTE 3). Note that we only list sources of pitfalls relevant to the considered metrics. Other sources of pitfalls are neglected for this table.

**(a) Counting metrics.** Considered metrics: Accuracy (Fig. SN 3.40), Balanced Accuracy (BA) (Fig. SN 3.41), Expected Cost (EC) (Fig. SN 3.44), F_*β*_ Score (Fig.SN 3.45), Matthews Correlation Coefficient (MCC) (Fig. SN 3.48), Net Benefit (NB) (Fig. SN 3.49), Negative Predictive Value (NPV) (Fig. SN 3.50), Positive Likelihood Ratio (LR+) (Fig. SN 3.52), Positive Predictive Value (PPV) (Fig. SN 3.53), Sensitivity (Sens) (Fig. SN 3.54), Specificity (Spec) (Fig. SN 3.55), Weighted Cohen’s Kappa (WCK) (Fig. SN 3.56).										
**Source of potential pitfall**	**Accuracy**	**BA**	**EC**	**F*_β_* Score**	**LR+**	**MCC**	**NB**	**PPV/NPV**	**Sens/Spec**	**WCK**
Importance of confidence awareness	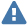 *	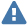 *	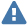 *	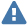 *	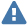 *	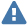 *	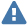 *	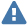 *	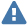 *	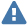 *
Importance of Comparability across data sets	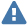 (Fig.SN 2.9)		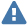 ** (Fig. SN 2.9)	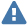 (Fig. SN 2.9)		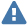 (Fig. SN 2.9)	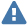 (Fig. SN 2.9)	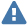 (Fig. SN 2.9)		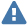 (Fig. SN 2.9)
Unequal severity of class confusions	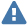 (Fig. 4b)	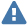 (Fig. 4b)		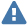 *** (Fig. 4b)	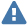 (Fig. 4b)	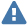 (Fig. 4b)		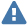 (Fig. 4b)	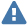 (Fig. 4b)	
Importance of cost-benefit analysis	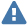 (Fig.SN 2.11)	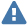 (Fig. SN 2.11)		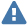 *** (Fig. SN 2.11)	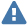 (Fig. SN 2.11)	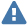 (Fig. SN 2.11)		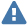 (Fig. SN 2.11)	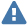 (Fig. SN 2.11)	
High class imbalance	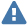 (Figs. 5a, SN 2.17)	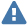 (Fig. 5a)	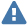 ** (Fig. 5a)		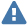 (Fig. 5a)		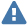 (Figs. 5a, SN 2.17)	**NPV:** 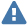 (Figs. 5a, SN 2.17)	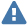 (**Sens:** Fig. 5a, **Spec:** Figs. 5a, SN 2.17)	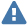 (Figs. 5a, SN 2.17)
Small test set size	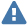 (Fig.SN 2.18)	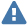 (Fig.SN 2.18)	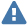 (Fig.SN 2.18)	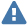 (Fig.SN 2.18)	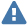 (Fig.SN 2.18)	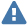 (Fig.SN 2.18)	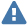 (Fig.SN 2.18)	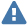 (Fig.SN 2.18)	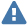 (Fig.SN 2.18)	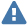 (Fig.SN 2.18)

(b) **Multi-threshold metrics.** Considered metrics: Area under the Receiver Operating Characteristic Curve (AUROC) (Fig. SN 3.57) and Average Precision (AP) (Fig. SN 3.58).		
**Source of potential pitfall**	**AP**	**AUROC**
Importance of confidence awareness	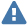 *	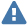 *
Importance of comparability across data sets	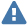 (Fig. SN 2.9)	
High class imbalance		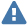 (Fig. 5a)
Small test set size	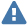 (Fig. SN 2.18)	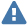 (Fig. SN 2.18)
Lack of predicted class scores	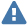 (Fig. SN 2.22)	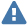 (Fig. SN 2.22)

* Discrimination metrics do not assess whether the predicted class scores reflect the confidence of the classifier. This is typically achieved with additional calibration metrics, which come with their own pitfalls (see Figs. SN 2.8 and SN 2.24, Extended Data Fig. 1b and the metric profiles in Suppl. Note 3.2).

** The weights in EC can be adjusted to avoid this pitfall.

*** The hyperparameter *β* can be used as a penalty for class confusions in the binary case. This property is not applicable to multi-class problems.

* Discrimination metrics do not assess whether the predicted class scores reflect the confidence of the classifier. This is typically achieved with additional calibration metrics, which come with their own pitfalls (see Figs. SN 2.8 and SN 2.24, Extended Data Fig. 1b and the metric profiles in Suppl. Note 3.2).

**Extended Data Tab. 2:** Overview of pitfall sources for semantic segmentation metrics ((a): overlap-based metrics, (b): boundary-based metrics) related to poor metric selection [P2]. A warning sign indicates a potential pitfall for the metric in the corresponding column, in case the property represented by the respective row holds true. Comprehensive illustrations of pitfalls are available in SUPPL. NOTE 2. A comprehensive list of pitfalls is provided separately for each metrics in the metrics cheat sheets (SUPPL. NOTE 3). Note that we only list sources of pitfalls relevant to the considered metrics. Other sources of pitfalls are neglected for this table.

(a) **Overlap-based metrics.** Considered metrics: Considered metrics: centerline Dice Similarity Coefficient (clDice) (Fig. SN 3.42), Dice Similarity Coefficient (DSC) (Fig. SN 3.43), F_*β*_ Score (Fig. SN 3.45), Intersection over Union (IoU) (Fig. SN 3.47).			
**Source of potential pitfall**	**clDice**	**DSC/IoU**	**F*p* Score**
Importance of structure boundaries	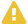 (Fig. 4a)	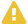 (Fig. 4a)	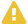 (Fig. 4a)
Importance of structure center(line)		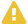 (Fig. SN 2.7, Extended Data Fig. 1b)	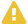 (Fig. SN 2.7, Extended Data Fig. 1b)
Unequal severity of class confusions	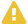 (Fig. SN 2.10)	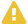 (Fig. SN 2.10)	
Small structure sizes	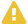 (Fig. SN 2.12, Extended Data Fig. 1a)	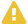 (Fig. SN 2.12, Extended Data Fig. 1a)	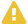 (Fig. SN 2.12, Extended Data Fig. 1a)
High variability of structure sizes	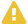 (Fig. SN 2.13)	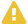 (Fig. SN 2.13)	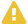 (Fig. SN 2.13)
Complex structure shapes		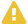 (Fig. SN 2.14)	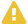 (Fig. SN 2.14)
Occurrence of overlapping or touching structures	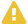 (Fig. SN 2.15)	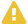 (Fig. SN 2.15)	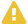 (Fig. SN 2.15)
Imperfect reference standard		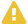 (Fig. SN 2.19)	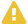 (Fig. SN 2.19)
Occurrence of cases with an empty reference	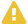 (Fig. SN 2.20))	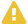 (Fig. SN 2.20))	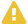 (Fig. SN 2.20))
Possibility of empty prediction	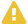 (Fig. SN 2.20))	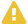 (Fig. SN 2.20))	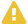 (Fig. SN 2.20))
Possibility of overlapping predictions	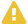 (Fig. SN 2.21, Extended Data Fig. 2a)	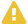 (Fig. SN 2.21, Extended Data Fig. 2a)	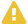 (Fig. SN 2.21, Extended Data Fig. 2a)

(b) **Boundary-based metrics.** Considered metrics: Average Symmetric Surface Distance (ASSD) (Fig. SN 3.60), Boundary Intersection over Union (Boundary IoU) (Fig. SN 3.61), Hausdorff Distance (HD) (Fig. SN 3.62), Hausdorff Distance 95th Percentile (HD95) (Fig. SN 3.65), Mean Average Surface Distance (MASD) (Fig. SN 3.63), Normalized Surface Distance (NSD) (Fig. SN 3.64).						
**Source of potential pitfall**	**ASSD**	**Boundary loU**	**HD**	**HD95**	**MASD**	**NSD**
Importance of structure volume	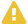 (Fig.SN 2.6)	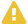 (Fig. SN 2.6)	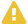 (Fig. SN 2.6)	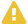 (Fig. SN 2.6)	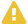 (Fig. SN 2.6)	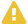 (Fig. SN 2.6)
Importance of structure center(line)	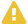 (Fig. SN 2.7, Extended Data Fig. 1b)	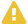 (Fig. SN 2.7, Extended Data Fig. 1b)	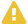 (Fig. SN 2.7, Extended Data Fig. 1b)	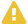 (Fig. SN 2.7, Extended Data Fig. 1b)	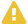 (Fig. SN 2.7, Extended Data Fig. 1b)	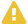 (Fig. SN 2.7, Extended Data Fig. 1b)
Occurrence of overlapping or touching structures	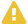 (Fig. SN 2.15)	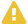 (Fig. SN 2.15)	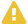 (Fig. SN 2.15)	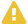 (Fig. SN 2.15)	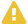 (Fig. SN 2.15)	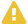 (Fig. SN 2.15)
Imperfect reference standard	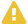 (Figs. 5c, SN 2.17)	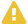 (Figs. 5c, SN 2.17)	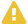 (Figs. 5c, SN 2.17)	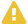 (Figs. 5c*, SN2.17)	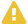 (Figs. 5c, SN 2.17)	
Occurrence of cases with an empty refer-ence	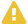 (Fig. SN 2.20))	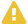 (Fig. SN 2.20))	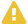 (Fig. SN 2.20))	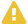 (Fig. SN 2.20))	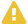 (Fig. SN 2.20))	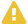 (Fig. SN 2.20))
Possibility of empty prediction	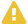 (Fig. SN 2.20))	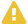 (Fig. SN 2.20))	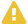 (Fig.SN 2.20))	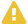 (Fig. SN 2.20))	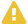 (Fig. SN 2.20))	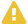 (Fig. SN 2.20))
Possibility of overlapping predictions	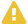 (Fig. SN 2.21, Extended Data Fig. 2a)	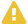 (Fig. SN 2.21, Extended Data Fig. 2a)	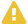 (Fig. SN 2.21, Extended Data Fig. 2a)	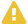 (Fig. SN 2.21, Extended Data Fig. 2a)	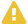 (Fig. SN 2.21, Extended Data Fig. 2a)	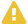 (Fig. SN 2.21, Extended Data Fig. 2a)

* Can be mitigated by the choice of the percentile.

**Extended Data Tab. 3:** Overview of sources of pitfalls for *object detection metrics* ((a): detection metrics, (b): localization criteria) related to poor metric selection [P2]. A warning sign indicates a potential pitfall for the metric in the corresponding column, in case the property represented by the respective row holds true. Comprehensive illustrations of pitfalls are available in SUPPL. NOTE 2. A comprehensive list of pitfalls is provided separately for each metrics in the metrics cheat sheets (SUPPL. NOTE 3). Note that we only list sources of pitfalls relevant to the considered metrics. Other sources of pitfalls are neglected for this table.

(a) **Detection metrics.** Considered counting metrics: F_*β*_ Score (Fig. SN 3.45), Positive Predictive Value (PPV) (Fig. SN 3.53), Sensitivity (Sens) (Fig. SN 3.54). Considered multi-threshold metrics: Average Precision (AP) (Fig. SN 3.58) and Free-Response Receiver Operating Characteristic (FROC) (Fig. SN 3.59).					
**Source of potential pitfall**	**F*_β_* Score**	**PPV**	**Sens**	**AP**	**FROC Score**
Unequal severity of class confusions	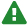 * (Fig. 4b)	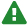 (Fig. 4b)	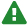 (Fig. 4b)	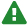 (Fig. 4b)	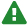 (Fig. 4b)
High class imbalance			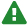 (Fig. 5a)		
Small test set size	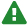 (Fig. SN 2.18)	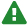 (Fig.SN 2.18)	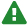 (Fig. SN 2.18)	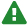 (Fig. SN 2.18)	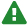 (Fig. SN 2.18)
Occurrence of cases with an empty reference	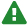 (Fig. SN 2.20, Extended Data Fig. 2b)	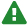 (Fig.SN 2.20, Extended Data Fig. 2b)	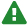 (Fig. SN 2.20, Extended Data Fig. 2b)	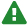 (Fig. SN 2.20, Extended Data Fig. 2b)	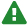 (Fig. SN 2.20, Extended Data Fig. 2b)
Possibility of empty prediction	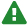 (Fig. SN 2.20, Extended Data Fig. 2b)	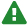 (Fig.SN 2.20, Extended Data Fig. 2b)	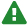 (Fig. SN 2.20, Extended Data Fig. 2b)	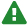 (Fig. SN 2.20, Extended Data Fig. 2b)	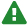 (Fig. SN 2.20, Extended Data Fig. 2b)
Lack of predicted class scores				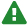 (Fig.SN 2.22)	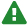 (Fig. SN 2.22)

(b) **Localization criteria.** Considered localization criteria: Box/Approx Intersection over Union (IoU) (Fig. SN 3.76), Center Distance (Fig. SN 3.74), Mask IoU > 0 (Fig. SN 3.77), and Point inside Mask/ Box/ Approx (Fig. SN 3.78).				
**Source of potential pitfall**	**Box/ Approx IoU**	**Center Distance**	**Mask IoU > 0**	**Point inside Mask/Box/Approx**
Importance of structure boundaries	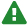 (Fig. 5a)	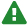 (Fig. 5a)	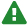 (Fig. 5a)	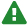 (Fig. 5a)
Importance of structure volume		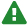 (Fig. SN 2.6)	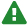 (Fig. SN 2.6)	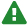 (Fig. SN 2.6)
Importance of structure center(line)	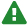 (Fig.SN 2.7, Extended Data Fig. 1b)		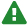 (Fig.SN 2.7, Extended Data Fig. 1b)	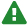 (Fig.SN 2.7, Extended Data Fig. 1b)
Unequal severity of class confusions	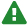 (Fig. SN 2.10)	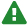 (Fig. SN 2.10)*	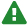 (Fig. SN 2.10)	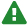 (Fig. SN 2.10)*
Small structure sizes	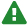 (Fig. SN 2.12, Extended Data Fig. 1a)			
Complex structure shapes	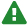 (Figs. SN 2.13, SN 2.16)	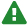 (Fig. SN 2.13)	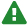 (Fig. SN 2.13)	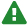 (Fig. SN 2.13)
Occurrence of disconnected structures	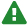 (Fig. SN 2.16)			Point inside Box: 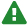 (Fig. SN 2.16)
Imperfect reference standard	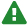 (Fig. 5c)			

* The hyperparameter *β* can be used as a penalty for class confusions in the binary case.

This property is not applicable to multi-class problems.

* Criterion implies point prediction, thus overlap assessment is not applicable.

**Extended Data Tab. 4:** Overview of sources of pitfalls for *instance segmentation metrics (Part 1)* ((a): detection metrics, (b): localization criteria) related to poor metric selection [P2]. A warning sign indicates a potential pitfall for the metric in the corresponding column, in case the property represented by the respective row holds true. Comprehensive illustrations of pitfalls are available in SUPPL. NOTE 2. A comprehensive list of pitfalls is provided separately for each metrics in the metrics cheat sheets (SUPPL. NOTE 3). Note that we only list sources of pitfalls relevant to the considered metrics. Other sources of pitfalls are neglected for this table.

(a) **Detection metrics.** Considered counting metrics: F_*β*_ Score (Fig. SN 3.45), Positive Predictive Value (PPV) (Fig. SN 3.53), Panoptic Quality (PQ) (Fig. SN 3.51), Sensitivity (Sens) (Fig. SN 3.54). Considered multi-threshold metrics: Average Precision (AP) (Fig. SN 3.58) and Free-Response Receiver Operating Characteristic (FROC) (Fig. SN 3.59).						
**Source of potential pitfall**	**F*_β_* Score**	**PPV**	**PQ**	**Sens**	**AP**	**FROC Score**
Unequal severity of class confusions	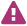 * (Fig. 4b)	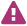 (Fig. 4b)	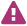 (Fig. 4b)	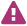 (Fig. 4b)		
High class imbalance				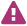 (Fig. 5a)		
Small test set size	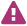 (Fig. SN 2.18)	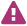 (Fig. SN 2.18)	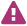 (Fig. SN 2.18)	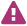 (Fig. SN 2.18)	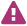 (Fig. SN 2.18)	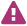 (Fig. SN 2.18)
Lack of predicted class scores					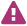 (Fig. SN 2.22)	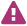 (Fig. SN 2.22)

(b) **Localization criteria.** Considered localization criteria: Boundary Intersection over Union (Boundary IoU) (Fig. SN 3.73), Intersection over Reference (IoR) (Fig. SN 3.75), Mask IoU (Fig. SN 3.47).			
**Source of potential pitfall**	**Boundary IoU**	**IoR**	**Mask IoU**
Importance of structure boundaries		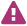 (Fig. 4a)	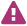 (Fig. 4a)
Importance of structure volume	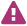 (Fig. SN 2.4)		
Importance of structure center(line)	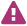 (Fig. SN 2.7, Extended Data Fig. 1b)	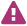 (Fig. SN 2.7, Extended Data Fig. 1b)	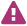 (Fig. SN 2.7, Extended Data Fig. 1b)
Unequal severity of class confusions	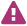 (Fig. SN 2.10)	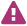 (Fig. SN 2.10)	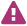 (Fig. SN 2.10)
Small structure sizes		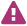 (Fig. SN 2.12, Extended Data Fig. 1a)	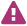 (Fig. SN 2.12, Extended Data Fig. 1a)
Complex structure shapes		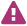 (Fig. SN 2.14)	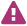 (Fig. SN 2.14)
Imperfect reference standard	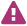 (Fig. SN 2.19)	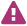 (Fig. SN 2.19)	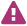 (Fig. SN 2.19)

* The hyperparameter *β* can be used as a penalty for class confusions in the binary case.

This property is not applicable to multi-class problems.

**Extended Data Tab. 5:** Overview of sources of pitfalls for *instance segmentation metrics (Part 2)* ((a) per instance segmentation overlap-based metrics, (b) per instance segmentation boundary-based metrics) related to poor metric selection [P2]. A warning sign indicates a potential pitfall for the metric in the corresponding column, in case the property represented by the respective row holds true. Comprehensive illustrations of pitfalls are available in SUPPL. NOTE 2. Note that we only list sources of pitfalls relevant to the considered metrics. Other sources of pitfalls are neglected for this table.

(a) **Per instance segmentation overlap-based metrics.** Considered metrics:centerline Dice Similarity Coefficient (clDice) (Fig. SN 3.42), Dice Similarity Coefficient (DSC) (Fig. SN 3.43), F_*β*_ Score (Fig. SN 3.45), Intersection over Union (IoU) (Fig. SN 3.47).			
**Source of potential pitfall**	**clDice**	**DSC/IoU**	**F*_β_* Score**
Importance of structure boundaries	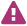 (Fig. 4a)	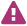 (Fig. 4a)	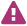 (Fig. 4a)
Importance of structure center(line)		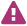 (Fig. SN 2.7, Extended Data Fig. 1b)	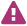 (Fig. SN 2.7, Extended Data Fig. 1b)
Unequal severity of class confusions	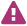 (Fig. SN 2.10)	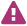 (Fig. SN 2.10)	
Small structure sizes	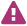 (Fig. SN 2.12, Extended Data Fig. 1a)	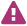 (Fig. SN 2.12, Extended Data Fig. 1a)	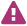 (Fig. SN 2.12, Extended Data Fig. 1a)
Complex structure shapes		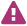 (Fig. SN 2.14)	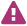 (Fig. SN 2.14)
Imperfect reference standard		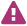 (Fig. SN 2.19)	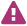 (Fig. SN 2.19)

(b) **Per instance segmentation boundary-based metrics.** Considered metrics: Average Symmetric Surface Distance (ASSD) (Fig. SN 3.60), Boundary Intersection over Union (Boundary IoU) (Fig. SN 3.61), Hausdorff Distance (HD) (Fig. SN 3.62), Hausdorff Distance 95th Percentile (HD95) (Fig. SN 3.65), Mean Average Surface Distance (MASD) (Fig. SN 3.63), Normalized Surface Distance (NSD) (Fig. SN 3.64).						
**Source of potential pitfall**	**ASSD**	**Boundary IoU**	**HD**	**HD95**	**MASD**	**NSD**
Importance of structure volume	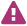 (Fig. SN 2.6)	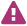 (Fig. SN 2.6)	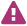 (Fig. SN 2.6)	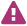 (Fig. SN 2.6)	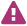 (Fig. SN 2.6)	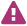 (Fig. SN 2.6)
Importance of structure center(line)	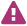 (Fig. SN 2.7, Extended Data Fig. 1b)	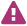 (Fig. SN 2.7, Extended Data Fig. 1b)	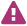 (Fig. SN 2.7, Extended Data Fig. 1b)	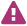 (Fig. SN 2.7, Extended Data Fig. 1b)	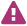 (Fig. SN 2.7, Extended Data Fig. 1b)	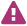 (Fig. SN 2.7, Extended Data Fig. 1b)
Imperfect reference standard	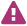 (Figs. 5c, SN 2.19)	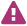 (Figs. 5c, SN 2.19)	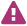 (Figs. 5c, SN 2.19)	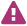 (Figs. 5c*, SN 2.19)	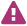 (Figs. 5c, SN 2.19)	

* Can be mitigated by the choice of the percentile.

## Supplementary Material

Supplementary Material

## Figures and Tables

**Fig. 3:**
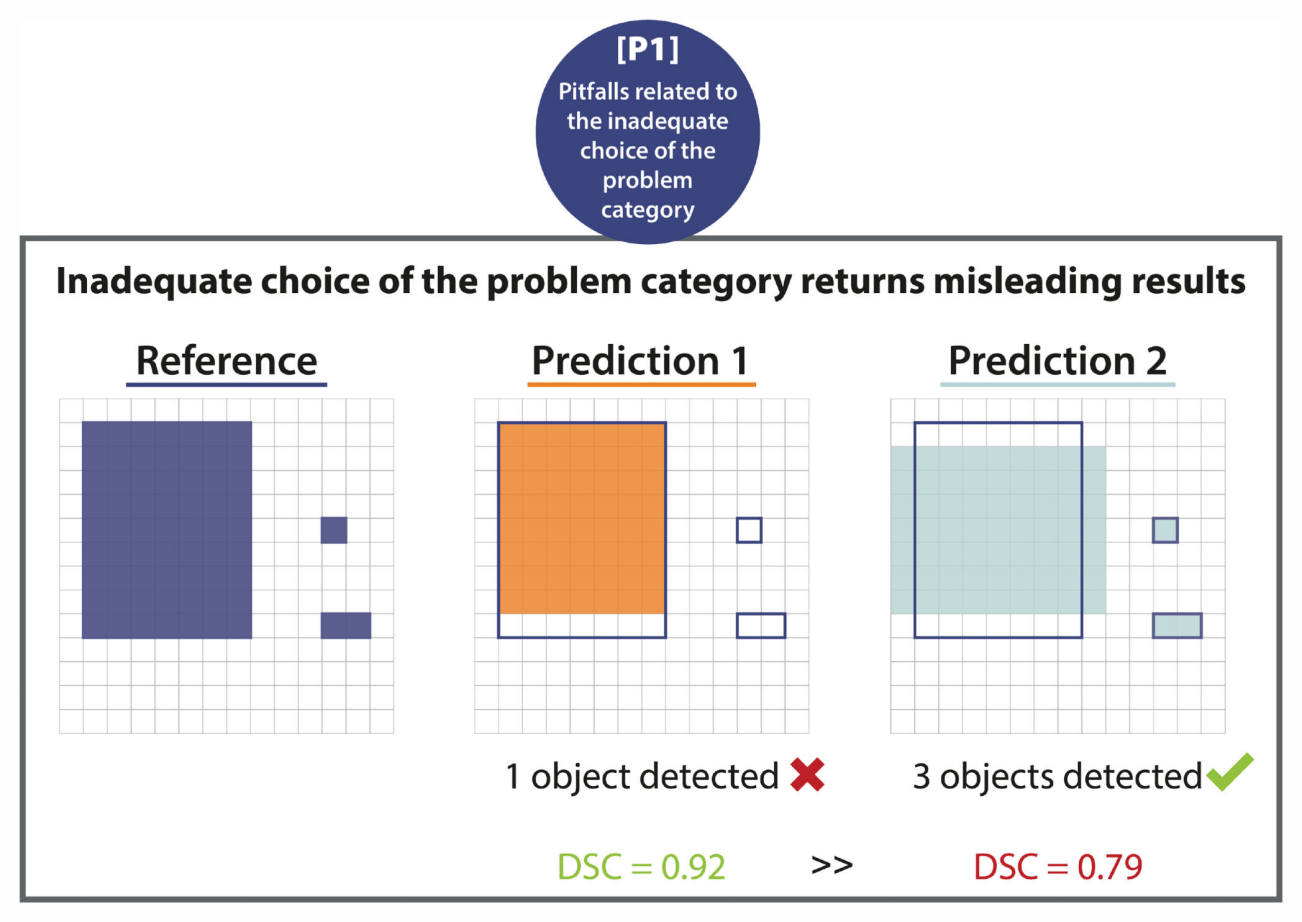
[P1] Pitfalls related to the inadequate choice of the problem category. **Wrong choice of problem category**. Effect of using segmentation metrics for object detection problems. The pixel-level Dice Similarity Coefficient (DSC) of a prediction recognizing every structure (*Prediction 2*) is lower than that of a prediction that only recognizes one of the three structures (*Prediction 1*).

## Data Availability

We provide reference implementations for all *Metrics Reloaded* metrics within the Medical Open Network for Artificial Intelligence (MONAI) open-source framework. They are accessible at https://github.com/Project-MONAI/MetricsReloaded.

## Footnotes

1
https://www.kaggle.com/competitions/data-science-bowl-2018/overview/evaluation

2
https://github.com/deepmind/surface-distance

3
https://torchmetrics.readthedocs.io/en/stable/classification/dice.html?highlight=dice
