# Supplementary material for: Understanding metric-related pitfalls in image analysis validation
Source: Nat Methods. Author manuscript; Available in PMC 2024 Aug 14. (PMC11181963; doi:10.1038/s41592-023-02150-0)
Supplement: Supplementary Material [file EMS197961-supplement-Supplementary_Material.pdf]

nature methods

Perspective

https://doi.org/10.1038/s41592-023-02150-0

Understanding metric-related pitfalls in image analysis validation

In the format provided by the authors and unedited

Supplementary Notes – Understanding metric-related pitfalls in image analysis validation

SUPPLEMENTARY METHODS

Literature search

The literature search of metric pitfalls and limitations was conducted on the platform Google Scholar. The checkbox "include patents" was activated and the checkbox "include citations" was deactivated; other default settings were left unchanged. For each metric, a specific search string using the Boolean operators `OR` and `AND` was generated as follows:

- (Different notations of the metric name, including synonyms and acronyms, enclosed in quotation marks, respectively, and combined with `OR`)
- `AND` "metric"
- `AND` (different expressions pertaining to the concept of pitfalls, limitations and flaws, enclosed in quotation marks, respectively, and combined with `OR`)

For example, the following search string was used for the literature search of Dice Similarity Coefficient (DSC) pitfalls: `("DSC" OR "Dice Similarity Coefficient" OR "Sørensen-Dice coefficient" OR "F1 score" OR "DCE") AND "metric" AND ("pitfall" OR "limitation" OR "caveat" OR "drawback" OR "shortcoming" OR "weakness" OR "flaw" OR "disadvantage” OR "suffer")`.

A second literature search dedicated to the pitfalls collected during the Delphi process was conducted on the platforms Google Scholar and Google. This search served the purpose of determining how many of the proposed pitfalls could be found in either existing research literature or online resources such as blogs, assuming that the issue is already roughly known to the person conducting the search. We further determined whether or not a found pitfall was presented in a visual manner. We analyzed the first three results pages (corresponding to thirty results) from each search platform and excluded our own previous work on metric pitfalls from the analysis.

Delphi process

The collection of pitfalls was achieved via a multi-stage Delphi process conducted among an international expert consortium comprised of more than 60 biomedical image analysis experts, as well as community feedback. A Delphi process is a structured group communication process that serves to pool opinions from an expert panel via a series of individual interrogations, usually in the form of questionnaires, interspersed with feedback from the respondents [8]. The technique is widely used for building consensus among experts in medicine, particularly in the development of best practices in areas where evidence may be limited, conflicting, or absent [49]. Expert selection was initially based on membership in major relevant societies such as the Biomedical Image Analysis ChallengeS (BIAS) initiative, the Medical Open Network for Artificial Intelligence (MONAI) Working Group for Evaluation, Reproducibility and Benchmarks, and the Medical Image Computing and Computer Assisted Interventions (MICCAI) Special Interest Group for Challenges (previously MICCAI board working group), as well as a track record of expertise in the areas of metrics, challenges and/or best practices. To reflect as broad a range of application areas and metric pitfalls as possible, the number of consortium members was increased throughout the process to a final

number of 62 members. The Delphi process comprised four surveys. Each survey was developed by the coordinating team of the process and sent out to the remaining members of the consortium. Upon completion, the coordinating team then analyzed the results and iteratively refined the list of pitfalls. The main stages of the compilation and consensus building process are detailed in the following:

(1) *Compilation of pitfall sources:* The primary purpose of the first survey was obtaining agreement on sources of pitfalls.
(2) *Collection of pitfalls:* The following survey specifically asked for concrete pitfalls in the presence of those problem characteristics.
(3) *Community feedback:* The proposed list of pitfalls was further complemented by social media-based feedback from the general scientific community.
(4) *Final agreement on pitfalls:* The subsequent survey served to obtain consensus agreement on which pitfalls to include. For each pitfall, it asked whether the pitfall should be included. In addition, the experts were given the opportunity to provide feedback on each pitfall and to suggest further pitfalls. The final collection of pitfalls was illustrated and all metric values were verified by two independent observers.
(5) *Creation of taxonomy:* The collected pitfalls were analyzed and a taxonomy was created. In the final survey, approval of the consortium for the structure and phrasing of the taxonomy and the assignment of specific pitfalls to the taxonomy was obtained.

Expert consortium

The expert consortium consisted of a total of 70 researchers (70% male, 30% female) from a total of 65 institutions. The majority of experts (50%) were professors, followed by postdoctoral researchers (39%). The median h-index of the consortium was 31.5 (mean: 36; minimum: 6; maximum: 113) and the median academic age was 18 years (mean: 19; minimum: 3; max: 42). Experts were from 19 countries and 5 continents. 60% of experts had a technical, 6% a clinical, 3% a biological, and 23% a mixed background. Of the 65 institutions, we could identify the number of employees for 89%. Of those, the majority of institutions had a size between 1,000 and 10,000 employees (57%), followed by even larger institutions between 10,000 and 100,000 employees (22%), and smaller institutions below 1,000 employees (20%). Only a small portion of institutions were above 100,000 employees (2%).

SUPPL. NOTE 1 METRIC FUNDAMENTALS

The present work focuses on biomedical image analysis problems that can be interpreted as classification tasks at the image, object, or pixel level. The vast majority of metrics for these problem categories are directly or indirectly based on epidemiological principles of True Positive (TP), False Negative (FN), False Positive (FP), True Negative (TN), i.e., the *cardinalities* of the so-called confusion matrix. The TP/FN/FP/TN are henceforth referred to as cardinalities. In the case of more than two classes C, we also refer to the entries of the $C \times C$ confusion matrix as cardinalities. For simplicity and clarity in notation, we restrict ourselves to the binary case in most examples. Cardinalities can be computed at the image (segment), object, or pixel level. They are typically computed by comparing the prediction of the algorithm to a reference annotation. Modern neural network-based approaches commonly require a threshold to be set in order to convert the algorithm output comprising predicted class scores (also referred to as continuous class scores) to a confusion matrix. For the purpose of metric recommendation, the available metrics can be broadly classified as follows (see also [9]):

- **Counting metrics** operate directly on the confusion matrix and express the metric value as a function of the cardinalities. In the context of segmentation, they are typically referred to as **overlap-based** metrics [58]. We distinguish **multi-class counting metrics**, which are defined for an arbitrary number of classes and invariant under class order, from **per-class counting metrics**, which are computed by treating one class as foreground/positive class and all other classes as background. Popular examples for the former include Matthews Correlation Coefficient (MCC) or Accuracy, while examples for the latter are Sensitivity, Specificity and DSC.
- **Multi-threshold metrics** operate on a dynamic confusion matrix, reflecting the conflicting properties of interest, such as high Sensitivity and high Specificity. Popular examples include the Area under the Receiver Operating Characteristic Curve (AUROC) and Average Precision (AP).
- **Distance-based metrics** have been designed for semantic and instance segmentation tasks. They operate exclusively on the TPs and rely on the explicit definition of object boundaries. Popular examples are the Hausdorff Distance (HD) and the Normalized Surface Distance (NSD).

Depending on the context (e.g., image-level classification *vs.* semantic segmentation task) and the community (e.g., medical imaging community *vs.* computer vision community), identical metrics are referred to with different terminology. For example, Sensitivity, True Positive Rate (TPR) and Recall refer to the same concept. The same holds true for the DSC and the F_1 Score. The most relevant metrics for the problem categories in the scope of this paper are introduced in the following.

Most metrics are recommended to be applied per class (except for the multi-class counting metrics), meaning that a potential multi-class problem is converted to multiple binary classification problems, such that each relevant class serves as the positive class once. This results in different confusion matrices depending on which class is used as the positive class.

1.1 Image-level Classification

Image-level classification refers to the process of assigning one or multiple labels, or *classes*, to an image. Modern algorithms usually output **predicted class scores** (or continuous class scores) between 0 and 1 for every image and class, indicating the probability of the image belonging

to a specific class. By introducing a threshold (e.g., 0.5), predictions are considered as positive (e.g., cancer = true) if they are above the threshold, or negative if they are below the threshold. Subsequently, predictions are assigned to the cardinalities (e.g., a cancer patient with prediction cancer = true is considered as TP) [15]. The most popular classification metrics are counting metrics, operating on a confusion matrix with fixed threshold on the class probabilities, and multi-threshold metrics, as detailed in the following.

Counting metrics. As stated previously, counting metrics rely on the confusion matrix. We distinguish between per-class and multi-class counting metrics. Popular multi-class counting metrics include:

Accuracy [60]: Fig. SN 3.38
Balanced Accuracy (BA) [60]: Fig. SN 3.39
Expected Cost (EC) (also referred to as Expected Prediction Error or Expected Loss) [7, 24, 32]: Fig. SN 3.42
Matthews Correlation Coefficient (MCC) (also referred to as Phi Coefficient) [46]: Fig. SN 3.46
Weighted Cohen's Kappa (WCK) (also referred to as Weighted Cohen‘s Kappa Coefficient, Weighted Kappa Statistic or Weighted Kappa Score) [13]: Fig. SN 3.54

Popular per-class counting metrics include:

F_β Score [12, 63]: Fig. SN 3.43
Net Benefit (NB) [64]: Fig. SN 3.47
Negative Predictive Value (NPV) [60]: Fig. SN 3.48
Positive Predictive Value (PPV) (also referred to as Precision) [60]: Fig. SN 3.51
Sensitivity (also referred to as Recall, TPR or Hit Rate) [60]: Fig. SN 3.52
Specificity (also referred to as Selectivity or True Negative Rate (TNR)) [60]: Fig. SN 3.53

Multi-threshold metrics. The classical counting metrics presented above rely on fixed thresholds to be set on the predicted class probabilities (if available), resulting in them being based on the cardinalities of the confusion matrix. **Multi-threshold metrics** overcome this limitation by calculating metric scores based on multiple thresholds. Popular examples are:

Area under the Receiver Operating Characteristic Curve (AUROC) (also referred to as Area under the Curve (AUC), AUC - ROC (Area under the Curve - Receiver Operating Characteristics), C-Index, C-Statistics) [31]: Fig. SN 3.55
Average Precision (AP) [23, 42]: Fig. SN 3.56

Calibration metrics. While most research in biomedical image analysis focuses on the discrimination capabilities of classifiers, a complementary property of relevance is the *calibration* of predicted class scores (also known as *confidence scores*). Intuitively speaking, a system is well-calibrated if the predicted class scores (i.e., the output of the model) reflect the true probabilities of the outcome. In practice, this means that calibrated scores match the empirical success rate of associated predictions. For a binary classification task, calibration implies that of all the data samples assigned a predicted score of 0.8 for the positive class, empirically, 80% belong to this class. Popular examples are:

Brier Score (BS) [26]: Fig. SN 3.64
Class-Wise Calibration Error (CWCE) [40, 41]: Fig. SN 3.65
Expected Calibration Error (ECE) [47]: Fig. SN 3.66

Expected Calibration Error Kernel Density Estimate ($\mathbf{ECE^{KDE}}$) [52] : Fig. SN 3.67
Kernel Calibration Error (KCE) [28, 68]: Fig. SN 3.68
Negative Log Likelihood (NLL) [14]: Fig. SN 3.69
Root Brier Score (RBS) [28]: Fig. SN 3.70

1.2 Semantic Segmentation

Semantic segmentation is commonly defined as the process of partitioning an image into multiple segments/regions. To this end, one or multiple labels are assigned to every pixel such that pixels with the same label share certain characteristics. Semantic segmentation can therefore also be regarded as pixel-level classification. As in image-classification problems, predicted class probabilities are typically calculated for each pixel, deciding on the class affiliation based on a threshold over the class scores [1]. In semantic segmentation problems, the pixel-level classification is typically followed by a post-processing step, in which connected components are defined as objects, and object boundaries are created accordingly. Semantic segmentation metrics can roughly be classified into: (1) counting metrics or overlap-based metrics, for measuring the overlap between the reference annotation and the prediction of the algorithm, (2) distance-based or boundary-based metrics, for measuring the distance between object boundaries, and (3) problem-specific metrics, measuring, for example, object volumes.

Counting metrics. The most frequently used segmentation metrics are **counting metrics**. In the context of segmentation they are also referred to as **overlap-based metrics**, as they essentially measure the overlap between a reference mask and the algorithm prediction. Popular examples of overlap-based metrics include:

Dice Similarity Coefficient (DSC) (also referred to as Sørensen–Dice Coefficient, F_1 Score, Balanced F Score) [20]: Fig. SN 3.41
Intersection over Union (IoU) (also referred to as Jaccard Index, Tanimoto Coefficient) [35]: Fig. SN 3.45
centerline Dice Similarity Coefficient (clDice) [57]: Fig. SN 3.40

Distance-based metrics. Overlap-based metrics are often complemented by **distance-based metrics** that operate exclusively on the TPs and compute one or several distances between the reference and the prediction. Besides few exceptions, distance-based metrics are often **boundary-based metrics** which focus on assessing the accuracy of object boundaries. Popular examples include:

Average Symmetric Surface Distance (ASSD) (also referred to as Weighted Bilateral Mean Contour Distance) [70]: Fig. SN 3.58
Boundary Intersection over Union (Boundary IoU) [10]: Fig. SN 3.59
Hausdorff Distance (HD) (also referred to as Maximum Symmetric Surface Distance, Hausdorff Metric, Pompeiu–Hausdorff Distance) [34]: Fig. SN 3.60
Hausdorff Distance 95th Percentile (HD95) [34]: Fig. SN 3.63
Mean Average Surface Distance (MASD) (also referred to as Mean Surface Distance) [6]: Fig. SN 3.61
Normalized Surface Distance (NSD) (also referred to as Normalized Surface Dice, Surface Distance, Surface Dice, Surface DSC) [50]: Fig. SN 3.62

Problem-specific segmentation metrics. While overlap- and distance-based metrics are the standard metrics used by the general computer vision community, biomedical applications often have special domain-specific requirements. In medical imaging, for example, the actual volume of an object (e.g., a tumor) may be of particular interest. In this case, **volume metrics** such as the *Absolute* or *Relative Volume Error* and the *Symmetric Relative Volume Difference* can be computed [48].

1.3 Object Detection

Object detection refers to the detection of one or multiple objects (or: instances) of a particular class (e.g., lesion) in an image [42]. The following description assumes single-class problems, but translation to multi-class problems is straightforward, as validation for multiple classes on object level is performed individually per class. Notably, as multiple predictions and reference instances may be present in one image, the predictions need to include localization information, such that reference and predicted objects can be matched. Important design choices with respect to the validation of object detection methods include:

(1) *How to represent an object?* Representation is typically composed of location information and a class affiliation. The former may for example take the form of a bounding box (i.e., a list of coordinates), a pixel mask, or the object's center point. Additionally, modern algorithms typically assign a confidence value to each object, representing the probability of a prediction corresponding to an actual object of the respective class. Note that a confusion matrix is later computed for a fixed threshold on the predicted class probabilities.[1]
(2) *How to decide whether a reference instance was correctly detected?* This step is achieved by applying the *localization criterion.* A localization criterion may, for example, be based on comparing the object centers of the reference and prediction or computing their overlap.
(3) *How to resolve assignment ambiguities?* The above step might lead to ambiguous matchings, such as two predictions being assigned to the same reference object. Several strategies exist for resolving such cases.

The following sections provide details on (1) applying the localization criterion, (2) applying the assignment strategy, and (3) computing the actual performance metrics.

Localization criterion. As one image may contain multiple objects or no object at all, the **localization criterion** or **hit criterion** measures the (spatial) similarity between a prediction (represented by a bounding box, pixel mask, center point or similar) and a reference object. It defines whether the prediction *hit/detected* (TP) or *missed* (FP) the reference. Any reference object not detected by the algorithm is defined as FN. Please note that TNs are not defined for object detection tasks. Popular localization criteria include:

Box/Approx Intersection over Union (IoU) [35]: Fig. SN 3.74
Mask IoU > 0 [35, 66]: Fig. SN 3.75
Center Distance [30]: Fig. SN 3.72
Point inside Mask/ Box/ Approx [2]: Fig. SN 3.76

[1]Please note that we will use the term confidence scores analogously to predicted class probabilities in the context of object detection and instance segmentation.
[2]https://cada.grand-challenge.org/Assessment/

Assignment strategy. The localization criterion alone is not sufficient to extract the final confusion matrix based on a fixed threshold for the predicted class probabilities (confidence scores), as ambiguities can occur. For example, two predictions may have been assigned to the same reference object in the localization step, or vice versa. These ambiguities need to be resolved in a further **assignment step**. This assignment and thus the resolving of potential assignment ambiguities can be done via different strategies:

Greedy (by Score) Matching [23]: Fig. SN 3.77
Optimal (Hungarian) Matching [38]: Fig. SN 3.79
Matching via Overlap > 0.5 [21]: Fig. SN 3.80
Greedy (by Localization Criterion) Matching [44]: Fig. SN 3.78

Metric computation. Similar to image-level classification and semantic segmentation algorithms, object detection algorithms are commonly assessed with counting metrics, assuming a fixed confusion matrix. Popular examples include:

F$_\beta$ Score [12, 63]: Fig. SN 3.43
False Positives per Image (FPPI) [5, 62]: Fig. SN 3.44
Positive Predictive Value (PPV) (also referred to as Precision) [60]: Fig. SN 3.51
Sensitivity (also referred to as Recall, TPR or Hit Rate) [60]: Fig. SN 3.52

Similarly, multi-threshold metrics rely on a range of thresholds. Popular examples are:

Average Precision (AP) [23, 42]: Fig. SN 3.56
Free-Response Receiver Operating Characteristic (FROC) Score [62]: Fig. SN 3.57

1.4 Instance Segmentation

In contrast to semantic segmentation, **instance segmentation** problems distinguish different instances of the same class (e.g., different lesions). Similarly to object detection problems, the task is to detect individual instances of the same class, but detection performance is measured by pixel-level correspondences (as in semantic segmentation problems). Optionally, instances can be applied to one of multiple classes. Validation metrics in instance segmentation problems often combine common detection metrics with segmentation metrics applied per instance. For instance, segmentation problems, we consider different localization criteria, namely:

Localization criteria:

Boundary Intersection over Union (Boundary IoU) [10]: Fig. SN 3.71
Mask IoU [35]: Fig. SN 3.74
Intersection over Reference (IoR) [45]: Fig SN 3.73

Additional counting metric: If detection and segmentation performance should be assessed simultaneously in a single score, the **Panoptic Quality (PQ)** metric can be utilized [36]: Fig. SN 3.49.

It should be noted that instance segmentation problems are often phrased as semantic segmentation problems with an additional post-processing step, such as connected component analysis [55].

SUPPL. NOTE 2 METRIC PITFALLS

This section presents common limitations of image processing metrics related to [P1] an inadequate choice of problem category (Suppl. Note 2.1), [P2] poor metric selection (Suppl. Note 2.2) and [P3] poor metric application (Suppl. Note 2.3) in an illustrated manner.

To preserve visual clarity, the most important of the presented metric values may be highlighted with color. Green metric values correspond to a "good" metric value (e.g. a high *Sensitivity* score), whereas red values correspond to a "bad" value (e.g. a low *Sensitivity*). Green check marks indicate desirable behavior of metrics, red crosses indicate undesirable behavior. Please note that a low metric value is not automatically a "bad" score. A metric value should always be put into perspective and compared to inter-rater variability. For simplicity, we still use the terms "good" and "bad/poor" throughout the section. Finally, our illustrations do not provide the concrete class probabilities of the presented classifiers.

2.1 Pitfalls related to an inadequate choice of the problem category

Performance metrics are typically expected to reflect a domain-specific (e.g., clinical) validation goal. Previous research, however, suggests that this is often not the case [56]. Before choosing validation metrics, the correct problem category needs to be defined. In the following, we present pitfalls related to metrics not being applied to the appropriate problem category. These can either be associated with a wrong choice of the problem category (here: Figs. 3 and SN 2.1; more examples are provided in [54]) or the lack of a matching problem category (Fig. SN 2.2).

Assessing object detection performance at image level yields misleading results

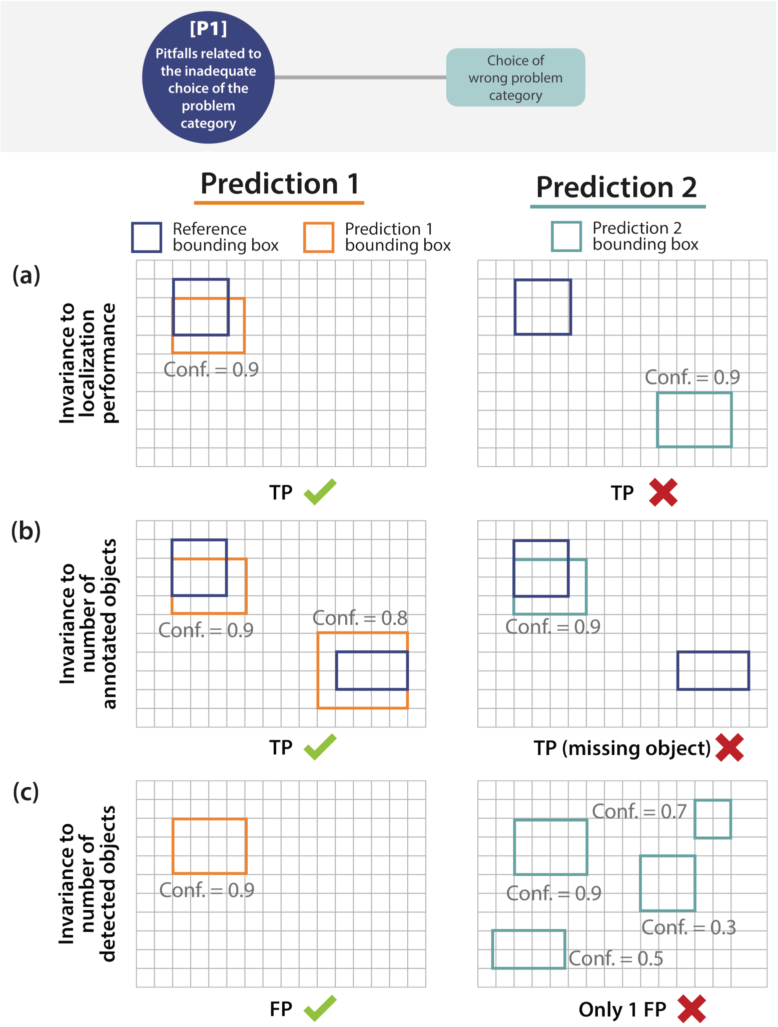

Fig. SN 2.1. Image-level classification metrics such as the Area under the Receiver Operating Characteristic Curve (AUROC) curve can be used to validate object detection models by first aggregating predictions to one image-level score (per class). This validation scheme discards the information on the object matching (localization, number of objects etc.). This leads to several problems: **(a)** The image-level Receiver Operating Characteristic (ROC) curve does not measure the localization performance. Both *Prediction 1* and *2* are considered as True Positive (TP) due to their score being very high, although *Prediction 2* does not hit the annotated object. **(b)** The image-level ROC is invariant to the number of annotated objects in an image. The curve does not discriminate between a model detecting all positives (*Prediction 1*) and a model detecting only one of the positives (*Prediction 2*), as long as the maximum score is the same. **(c)** The image-level ROC is invariant to the number of detections in an image. The curve does not discriminate between a model with many False Positives (FP) (*Prediction 2*), and a model with just one FP (*Prediction 1*), as long as the maximum score is the same. The class probabilities are represented by confidence scores (Conf.).

Common metrics may not reflect the domain interest

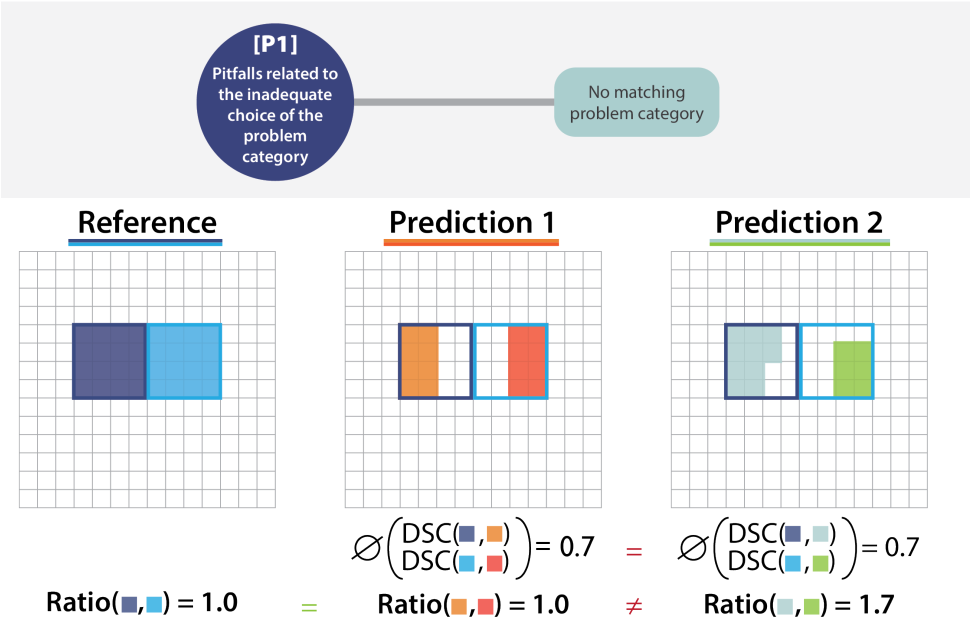

Fig. SN 2.2. In the absence of a matching problem category for the problem at hand, it may not be possible to find a common metric that ideally captures the domain interest. In this example, accuracy of the ratio between two volumes is the property of interest (e.g., the percentage of blood volume ejected in each cardiac cycle [4]). Using overlap-based segmentation metrics (here: Dice Similarity Coefficient (DSC)) to measure the volumetric ratio may be misleading. *Predictions 1* and *2* result in similar averaged DSC metric values although they result in a different ratio between structure volumes, which is the parameter of interest. ∅ refers to the average DSC values.

2.2 Pitfalls related to poor metric selection

Validation metrics typically assess a specific property of interest. Thus, a metric designed for a particular purpose often cannot be used to appropriately validate another property. This is due to both the limitations as well as the mathematical properties of individual metrics, both of which are often neglected. In this section, we present pitfalls related to poor metric selection.

2.2.1 Pitfalls related to disregard of the domain interest. Several requirements for metric selection arise from the domain interest, which may clash with particular metric limitations. In the following, we present pitfalls related to disregard of the domain interest, stemming from the following sources:

- Importance of structure boundaries (Figs. 4a and SN 2.3)
- Importance of structure volume (Fig. SN 2.4)
- Importance of structure center(line) (Fig. SN 2.5)
- Importance of confidence awareness (Fig. SN 2.6)
- Importance of comparability across data sets (Figs. SN 2.7)
- Unequal severity of class confusions (Figs. 4b and SN 2.8)
- Importance of cost-benefit analysis (Fig. SN 2.9)

Volume-based metrics alone are inadequate for assessing performance

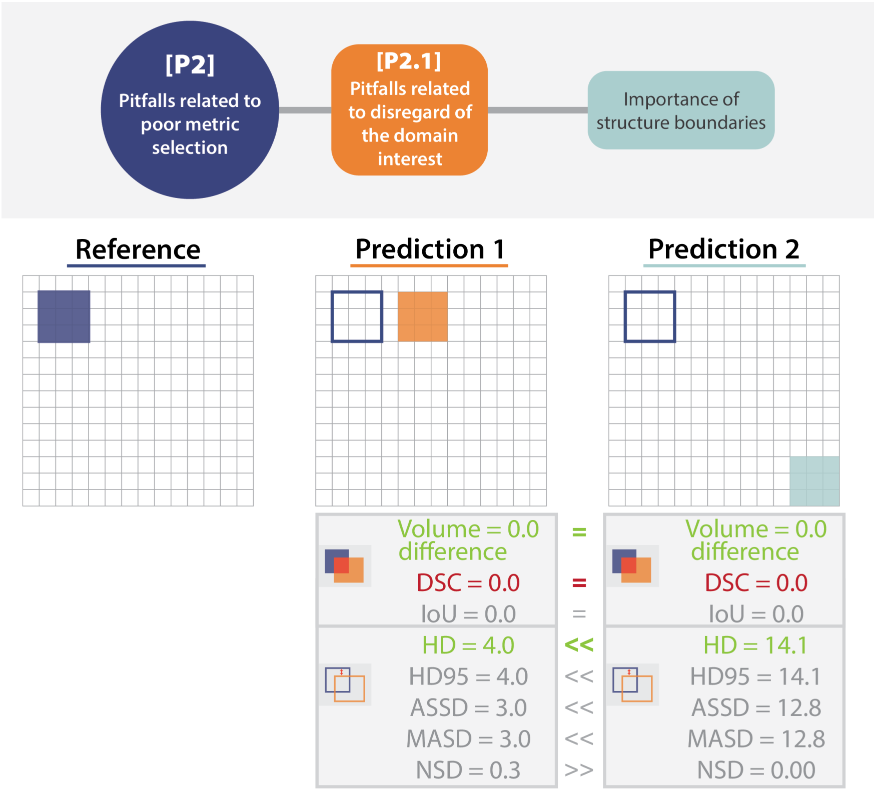

Fig. SN 2.3. Effect of only focusing on object volume. Both *Predictions 1* and *2* result in the correct volume difference of 0, but do not overlap the reference (Dice Similarity Coefficient (DSC) and Intersection over Union (IoU) of 0). Only the boundary-based measures (Hausdorff Distance (HD), Hausdorff Distance 95th Percentile (HD95), Average Symmetric Surface Distance (ASSD), Mean Average Surface Distance (MASD), and Normalized Surface Distance (NSD)) recognize the mislocalization. This pitfall is also relevant for localization criteria such as Box/Approx/Mask IoU, Center Distance, Mask IoU > 0, Point inside Mask/Box/Approx, and Intersection over Reference (IoR) .

Boundary-based metrics disregard holes in the segmentation

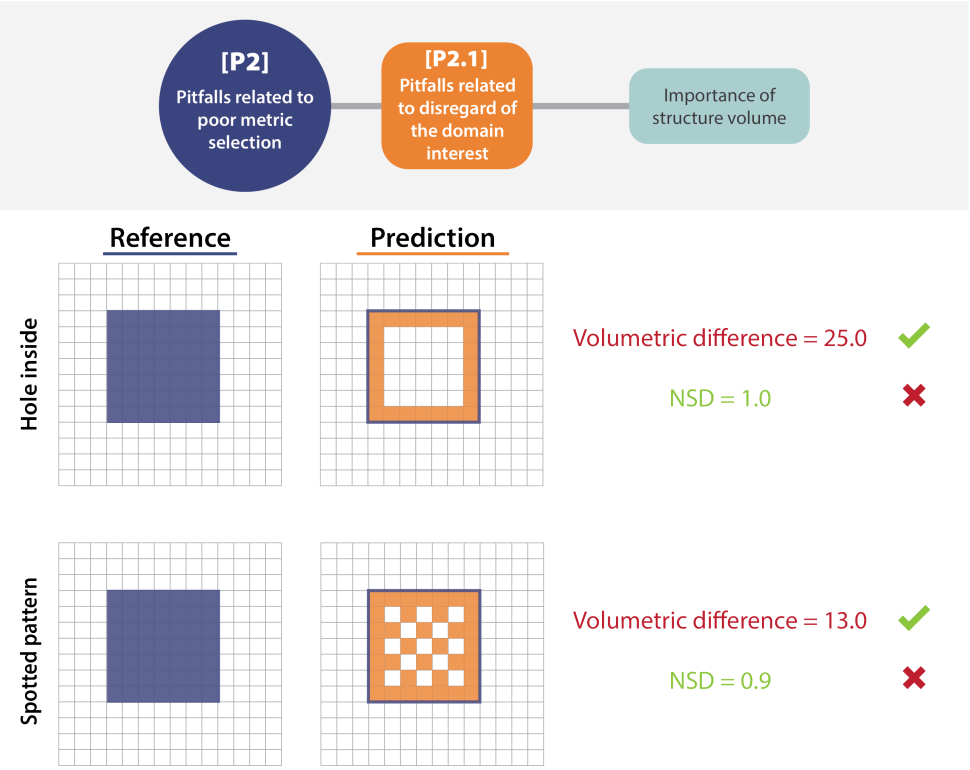

Fig. SN 2.4. Boundary-based metrics commonly ignore the overlap between structures and are thus insensitive to holes in structures. In the examples, the Prediction respectively features a hole or spotted pattern within the object. Boundary-based metrics (here: Normalized Surface Distance (NSD)) do not recognize this problem, yielding (near) perfect metric scores of 1.0 and 0.9, whereas the volumetric difference reflects the fact that the inner area is inadequately predicted. NSD was calculated for $\tau = 2$. This pitfall is also relevant for other boundary-based metrics such as Average Symmetric Surface Distance (ASSD), Boundary Intersection over Union (Boundary IoU), Hausdorff Distance (HD), Hausdorff Distance 95th Percentile (HD95), and Mean Average Surface Distance (MASD), as well as localization criteria such as Center Distance, Mask IoU > 0, Point inside Mask/Box/Appeox, Boundary IoU, Intersection over Reference (IoR), and Mask IoU.

Overlap-based metrics are unaware of object centers

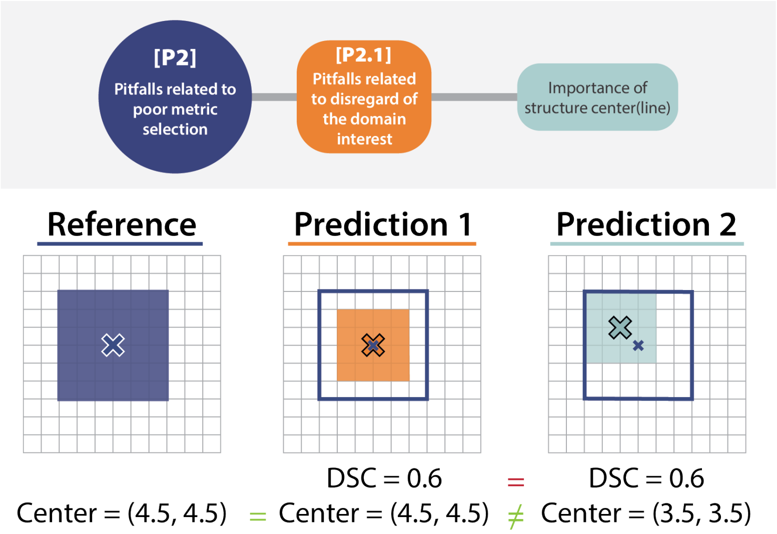

Fig. SN 2.5. The most common counting-based metrics are poor proxies for the center point alignment. Here, *Predictions 1* and *2* yield the same Dice Similarity Coefficient (DSC) value although *Prediction 1* approximates the location of the object much better. This pitfall is also relevant for other boundary- and overlap-based metrics such as Average Symmetric Surface Distance (ASSD), Boundary Intersection over Union (IoU), Hausdorff Distance (HD), Hausdorff Distance 95th Percentile (HD95), IoU, pixel-level F_β Score, and Mean Average Surface Distance (MASD), and localization criteria such as Box/Approx/Mask IoU, Mask IoU > 0, Point inside Mask/Box/Approx, Boundary IoU, and Intersection over Reference (IoR).

Common calibration metrics falsely imply perfect calibration

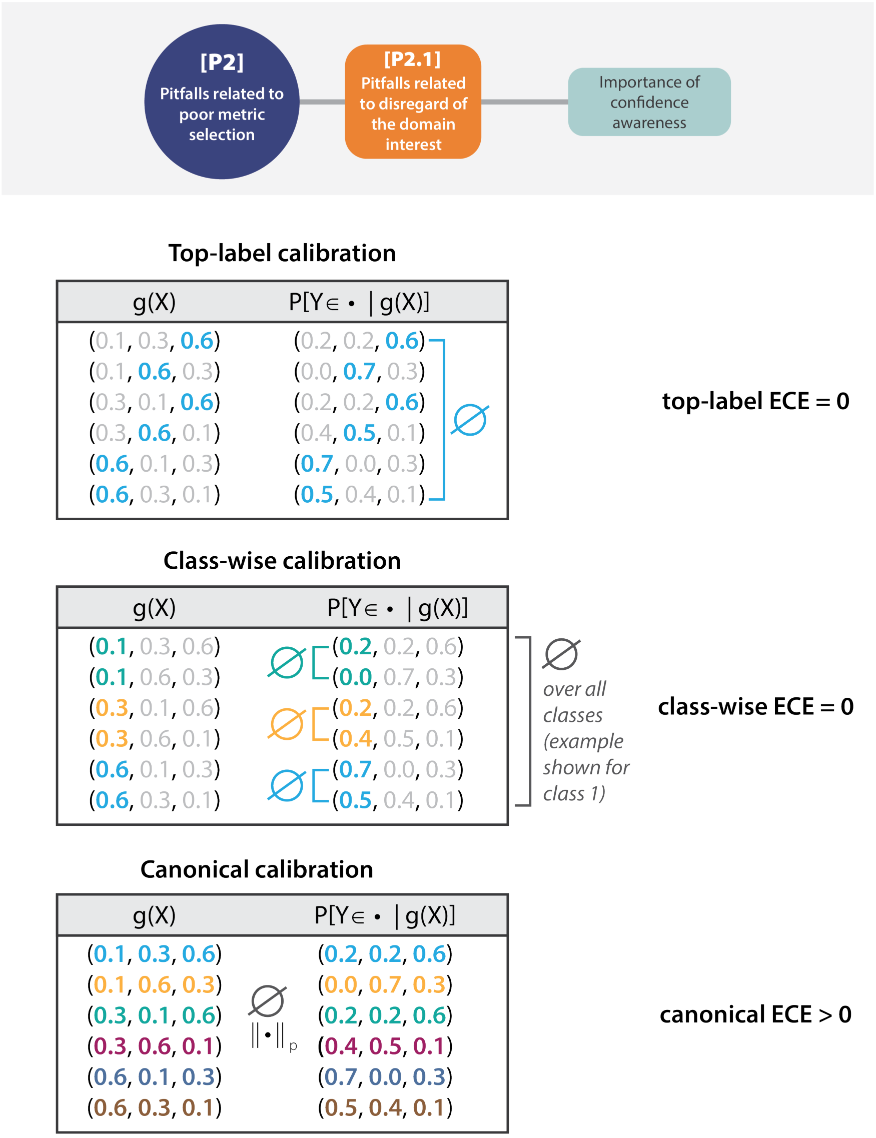

Fig. SN 2.6. Effect of different definitions of calibration on the Expected Calibration Error (ECE) when focusing on confidence or predicted class scores (confidence awareness). For top-label calibration, only the maximum values of the predicted class scores $g(X)$ are considered, while all other values are neglected, resulting in a perfect calibration for this example. Similarly, for class-wise calibration, the predicted class scores are compared class-wise per value, also yielding a perfect score. Only canonical calibration considers all components of the predicted class score vectors, showing that the model is not perfectly calibrated [28, 61]. A more detailed insight in different definitions of calibration is given in [44]. It should be noted that discrimination metrics generally do not assess calibration performance, i.e., perfect discrimination does not imply good calibration performance.

Comparison of metric scores across data sets may be misleading

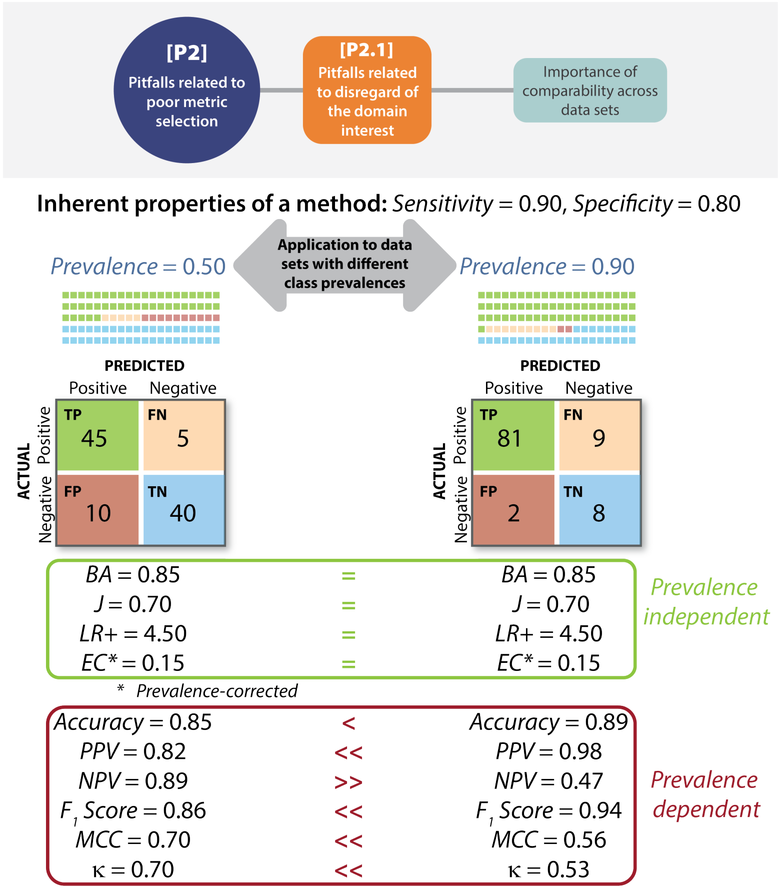

Fig. SN 2.7. Effect of prevalence dependency. An algorithm with specific inherent properties (here: Sensitivity of 0.9 and Specificity of 0.8) may perform completely differently on different data sets if the prevalences differ (here: 50% (left) and 90% (right)) and prevalence-dependent metrics are used for validation (here: Accuracy, Positive Predictive Value (PPV), Negative Predictive Value (NPV), F_1 Score, Matthews Correlation Coefficient (MCC), Cohen's Kappa κ). In contrast, prevalence-independent metrics (here: Balanced Accuracy (BA), Youden's Index J, Positive Likelihood Ratio (LR+), and Expected Cost (EC)) can be used to compare validation results across different data sets. Used abbreviations: True Positive (TP), False Negative (FN), False Positive (FP) and True Negative (TN). This pitfall is also relevant for other counting metrics such as Net Benefit (NB).

Overlap-based metrics prefer oversegmentation over undersegmentation

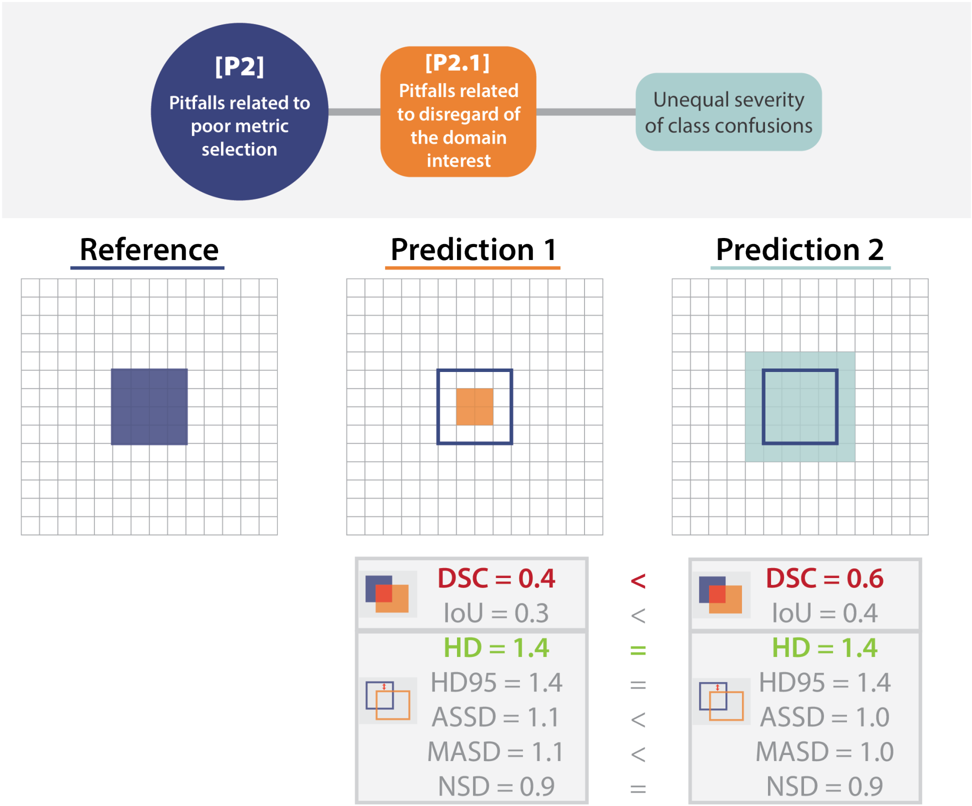

Fig. SN 2.8. Effect of undersegmentation *vs.* oversegmentation. The outlines of the predictions of two algorithms (*Prediction 1/2*) differ in only a single layer of pixels (*Prediction 1*: undersegmentation – smaller structure compared to reference, *Prediction 2*: oversegmentation – larger structure compared to reference). This has no (or only a minor) effect on the Hausdorff Distance (HD)/(95%), the Normalized Surface Distance (NSD), MASD, and the Average Symmetric Surface Distance (ASSD), but yields a substantially different Dice Similarity Coefficient (DSC) or Intersection over Union (IoU) score [58, 71]. If penalizing of either over- or undersegmentation is desired (unequal severity of class confusions), other metrics such as the F_β Score provide specific penalties for either depending on the chosen hyperparameter β. This pitfall is also relevant for other overlap-based metrics such as centerline Dice Similarity Coefficient (clDice) and localization criteria such as Box/Approx/Mask IoU, Boundary IoU, and Intersection over Reference (IoR).

Common metrics disregard cost-benefit analysis

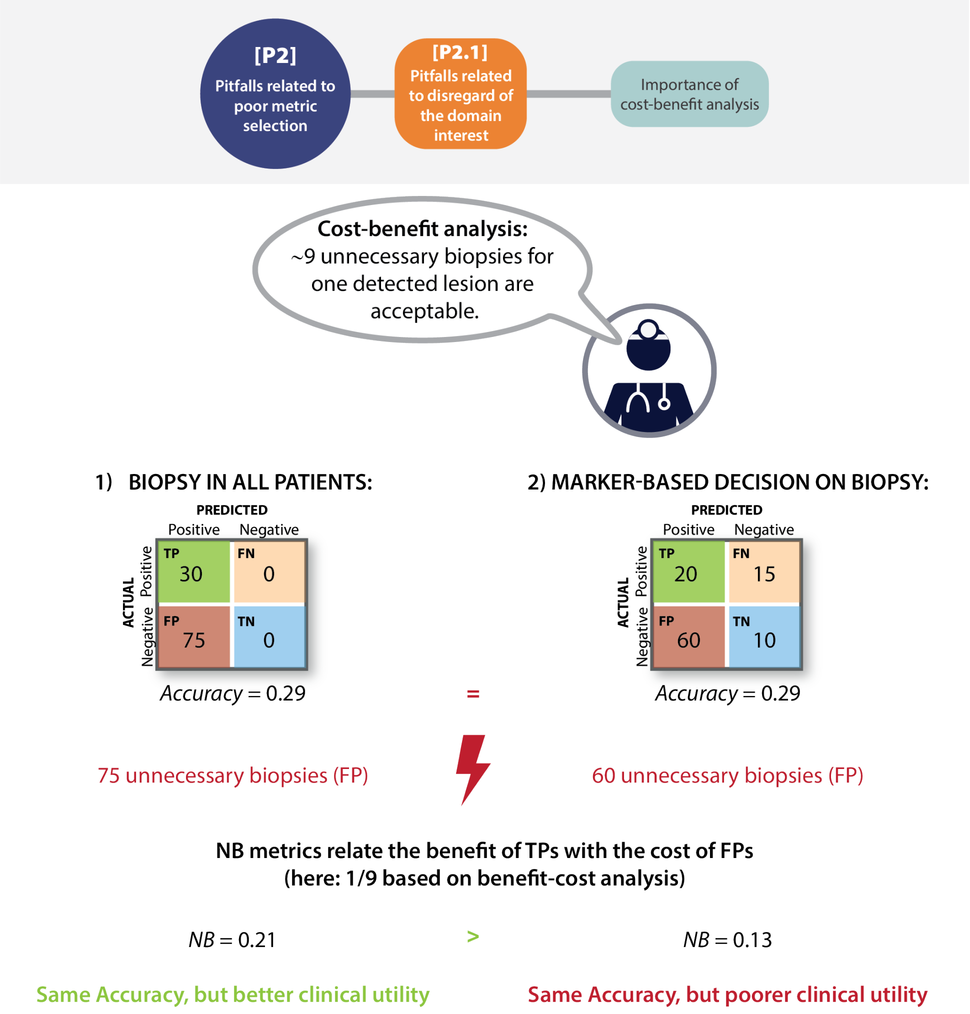

Fig. SN 2.9. Effect of neglecting a cost-benefit analysis. In a cost-benefit analysis, clinicians are able to define a risk-specific exchange rate that is used in the computation of the Net Benefit (NB) metric. Common metrics such as Accuracy do not consider this analysis and would favor the marker-based decision on biopsy, while NB indicates that biopsies of all patients actually yield a better clinical outcome [65]. This pitfall is also relevant for other counting metrics such as Balanced Accuracy (BA), Positive Likelihood Ratio (LR+), Matthews Correlation Coefficient (MCC), Negative Predictive Value (NPV), Positive Predictive Value (PPV), Sensitivity, and Specificity. For binary problems, the hyperparameter β of the F_β Score can be used as a dynamic penalty for class confusions.

2.2.2 Pitfalls related to disregard of the properties of the target structure. For problems that require capturing local properties (object detection, semantic or instance segmentation), the properties of the target structures to be localized and/or segmented may have severe implications for metric choice. Pitfalls can be further subdivided into *size-related* and *shape- and topology-related* pitfalls. In the following, we present pitfalls stemming from the following sources:

Size-related pitfalls:

- Small structure sizes (Extended Data Fig. 1a and Fig. SN 2.10)
- High variability of structure sizes (Fig. SN 2.11)

Shape- and topology-related pitfalls

- Complex structure shapes (Extended Data Fig. 1b and Fig. SN 2.12)
- Occurrence of overlapping or touching structures (Fig. SN 2.13)
- Occurrence of disconnected structures (Fig. SN 2.14)

Common localization criteria and overlap-based metrics are sensitive to structure sizes

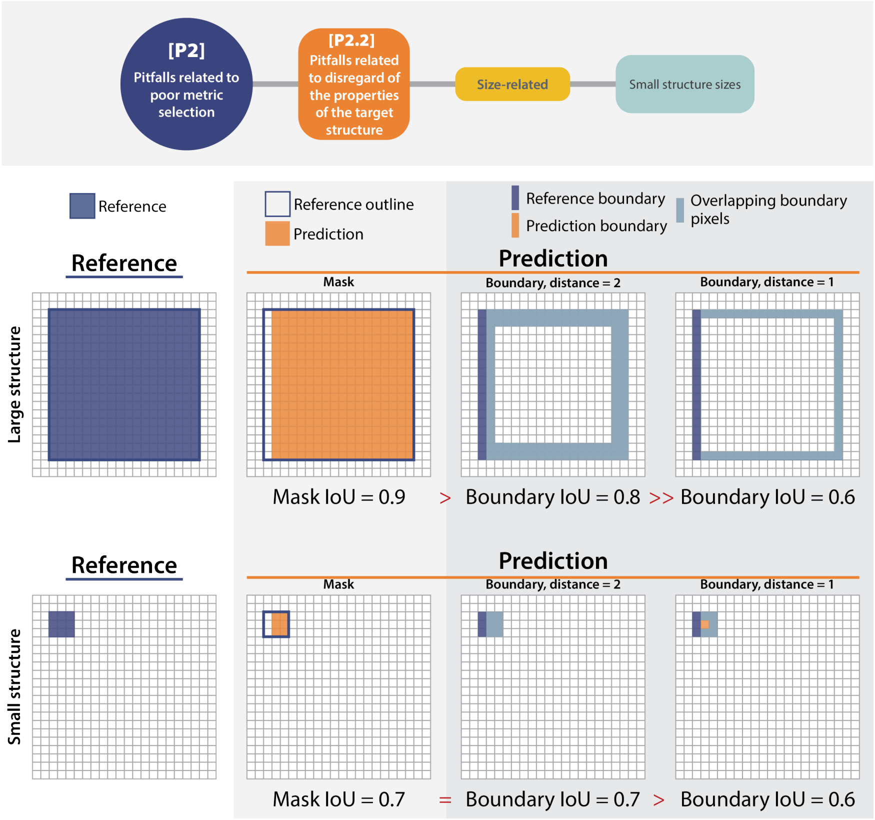

Fig. SN 2.10. Comparison of Mask and Boundary Intersection over Union (IoU) localization criteria in the case of particular importance of structure boundaries. Overlapping pixels from the reference and prediction are shown in light blue. The Mask IoU (second column) is less sensitive to boundary errors for large objects. The Boundary IoU (third and fourth column) especially considers contours, (1) yields smaller metric scores, thus penalizing errors in the boundaries, and (2) is more invariant to structure sizes, leading to very similar values for large and small structures (fourth column) [10]. This pitfall is also relevant for other overlap-based metrics such as centerline Dice Similarity Coefficient (clDice), Dice Similarity Coefficient (DSC), and pixel-level F_β Score, as well as localization criteria such as Box/Approx IoU and Intersection over Reference (IoR).

Effect of high variability of structure sizes

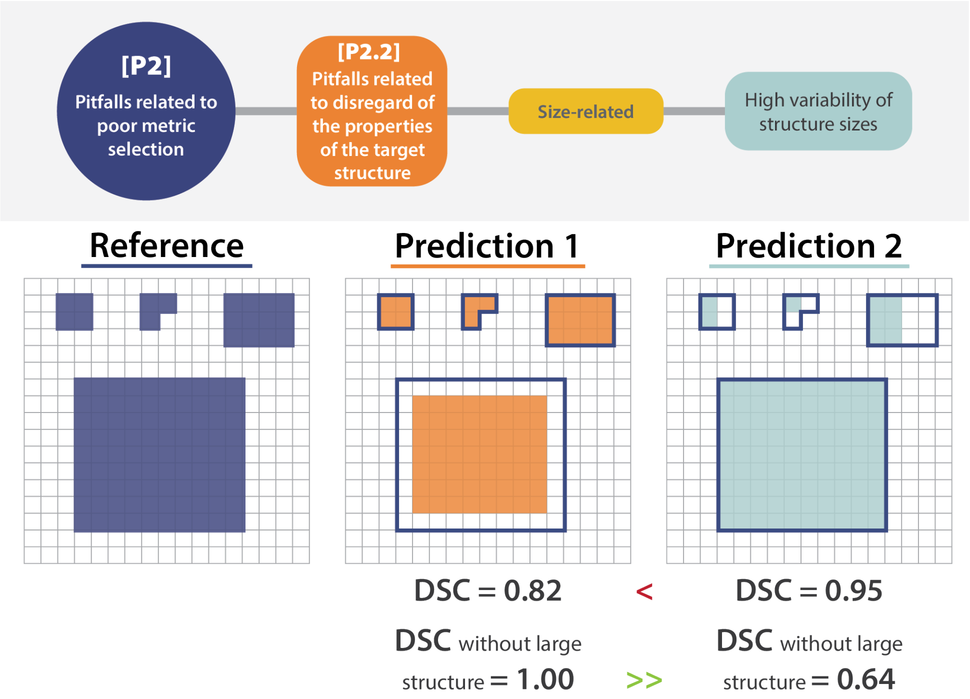

Fig. SN 2.11. Large structures completely dominate overlap-based metrics in semantic segmentation problems. While *Prediction 1* perfectly segments all three small structures, the metric score (here: Dice Similarity Coefficient (DSC)) is much worse compared to the score of *Prediction 2*, with only one perfect prediction for the large structure. This is highlighted by only computing the metric without the large structure. This pitfall is also relevant for other overlap-based metrics such as centerline Dice Similarity Coefficient (clDice), Dice Similarity Coefficient (DSC), and pixel-level F_β Score, as well as localization criteria such as Mask/Box/Approx Intersection over Union (IoU) and Intersection over Reference (IoR).

Common metrics are unaware of object shapes

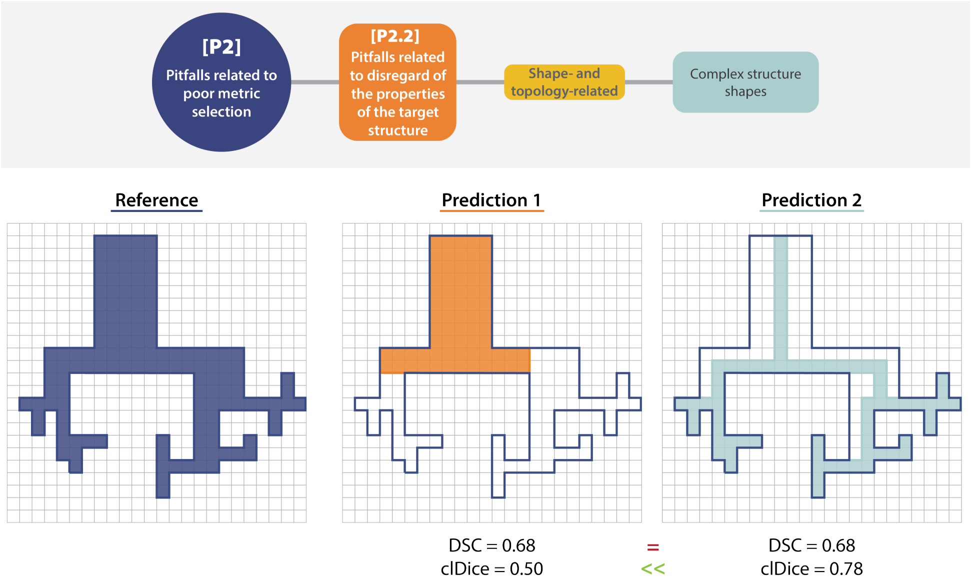

Fig. SN 2.12. Effect of complex shapes. Common overlap-based metrics such as the Dice Similarity Coefficient (DSC) are unaware of complex structure shapes and treat *Predictions 1* and *2* equally. The centerline Dice Similarity Coefficient (clDice) uncovers that *Prediction 1* misses the fine-granular branches of the reference and favors *Prediction 2*, which focuses on the object's center line and better captures its fine branches. This pitfall is also relevant for other overlap-based metrics such as Intersection over Union (IoU) and pixel-level F_β Score, and localization criteria such as Box/Approx/Mask IoU, Center Distance, Mask IoU > 0, Point inside Mask/Box/Approx, and Intersection over Reference (IoR).

Common metrics do not account for hierarchical label structure

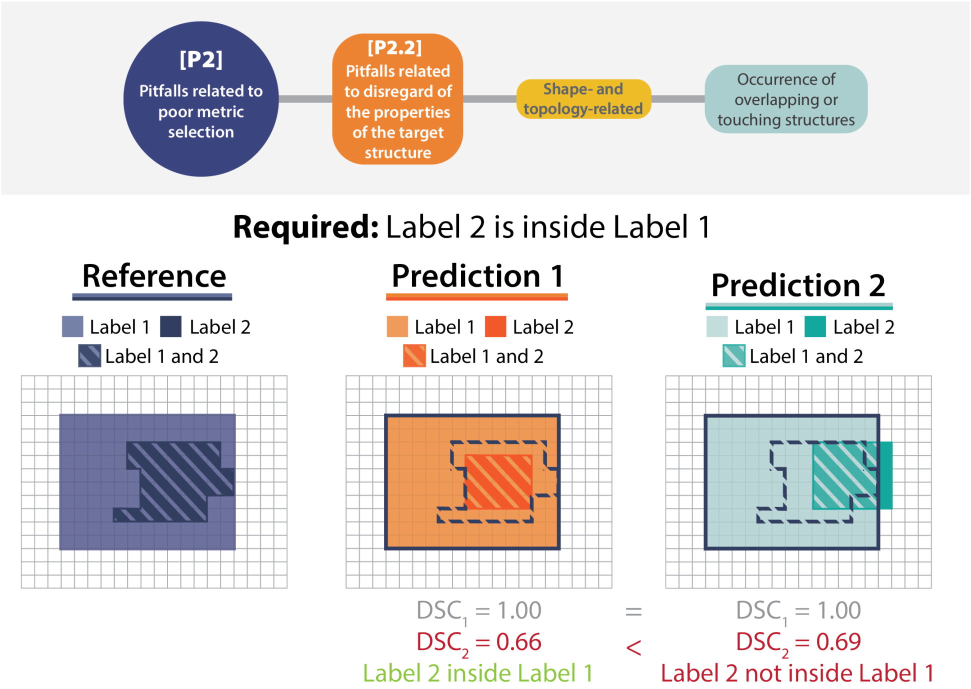

Fig. SN 2.13. Effect of nested multi-label structures. The requirement of *Label 2* being inside *Label 1* is violated by *Prediction 2*. Nevertheless, *Prediction 2* has a higher Dice Similarity Coefficient (DSC) score compared to *Prediction 1*, which adheres to the requirement. This pitfall is also relevant for other boundary- and overlap-based metrics such as Average Symmetric Surface Distance (ASSD), Boundary Intersection over Union (IoU), centerline Dice Similarity Coefficient (clDice), Hausdorff Distance (HD), Hausdorff Distance 95th Percentile (HD95), IoU, pixel-level F_β Score, Mean Average Surface Distance (MASD), and Normalized Surface Distance (NSD).

Bounding boxes are inadequate for representing complex shapes and disconnected structures

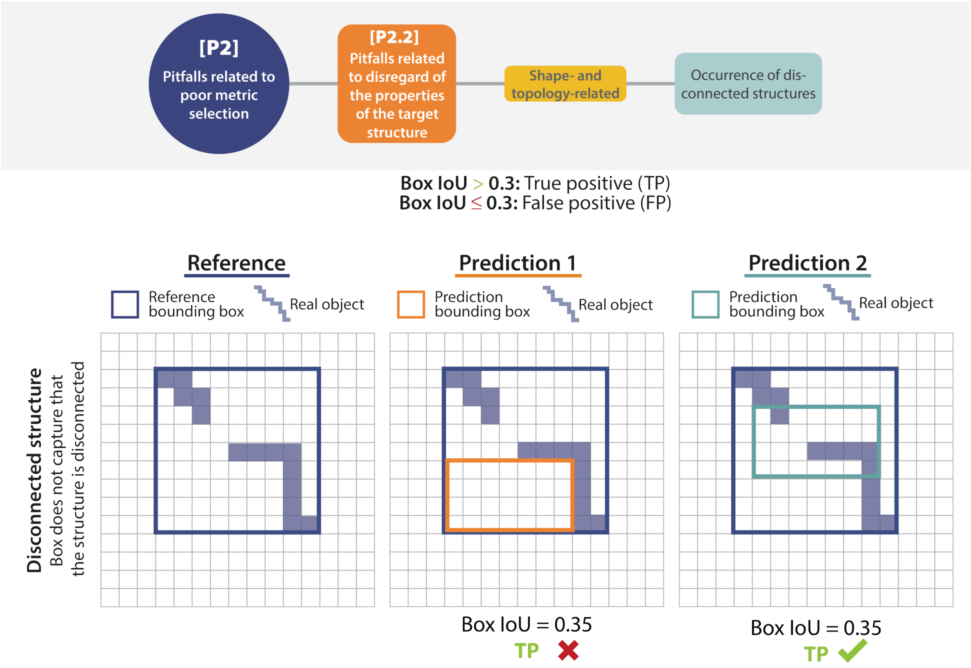

Fig. SN 2.14. Bounding boxes are not well-suited for representing disconnected shapes, in particular multi-component structures. *Predictions 1* and *2* both yield a True Positive (TP) detection, as the Box Intersection over Union (IoU) is larger than the threshold 0.3. However, *Prediction 1* does not hit the real object at all.

2.2.3 Pitfalls related to disregard of the properties of the data set and algorithm output. Properties of the data set such as class imbalances or high inter-rater variability may directly affect metric values. Pitfalls can be further subdivided into *class-related* and *reference-related* pitfalls. For reference-based metrics, the algorithm output will be compared against the reference annotation to compute a metric score. Thus, the content and format of the prediction is of high relevance for metric choice. In the following, we present pitfalls stemming from the following sources:

[P2.3] Disregard of the properties of the data set

- High class imbalance (Figs. 5a and SN 2.15)
- Small test set size (Figs. 5b and SN 2.16)
- Imperfect reference standard (Figs. 5c and SN 2.17)

[P2.4] Disregard of the properties of the algorithm output

- Possibility of empty prediction (Extended Data Fig. 2b and Fig. SN 2.18)
- Possibility of overlapping predictions (Extended Data Fig. 2a and Fig. SN 2.19)
- Lack of predicted class scores (Fig. SN 2.20)

Common metrics yield implausible results in the presence of class imbalance

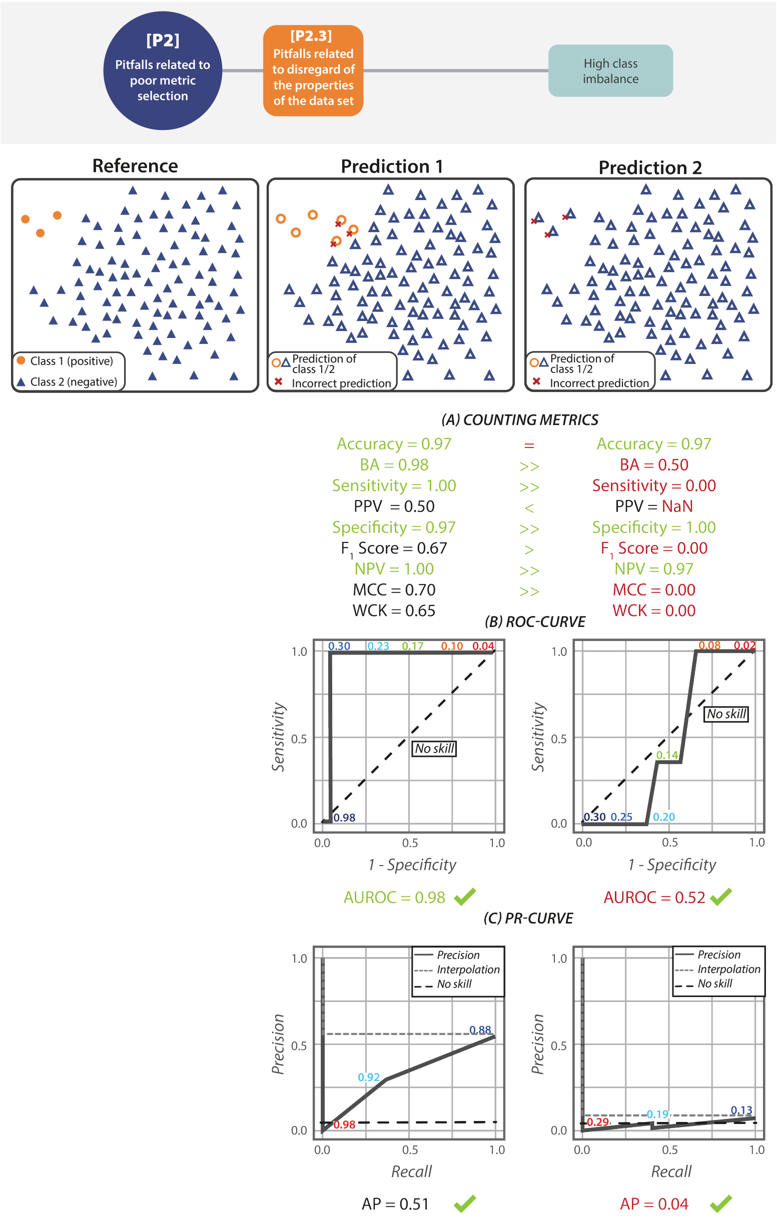

Fig. SN 2.15. Effect of class imbalance. Not every metric is designed to reflect class imbalance [11]. In the case of underrepresented classes, an unsuitable metric, such as Accuracy, yields a high value even if the classifier performs very poorly for one of the classes (here: *Prediction 2*). Multi-threshold metrics, such as the Area under the Receiver Operating Characteristic Curve (AUROC) and the Average Precision (AP), reveal the weakness, indicating that *Prediction 2* is not better than random guessing. For comparison, a no-skill classifier (random guessing) is shown as a black dashed line. For the Precision-Recall (PR) curves, the interpolation applied to compute the AP metric is shown as a dashed grey line. Thresholds used for curve generation are provided as small numbers above the curve. Further abbreviations: Positive Predictive Value (PPV), Negative Predictive Value (NPV), Matthews Correlation Coefficient (MCC), Weighted Cohen's Kappa (WCK). This pitfall is also relevant for other counting metrics such as Net Benefit (NB).

Common multi-threshold metrics are not well-suited for small sample sizes

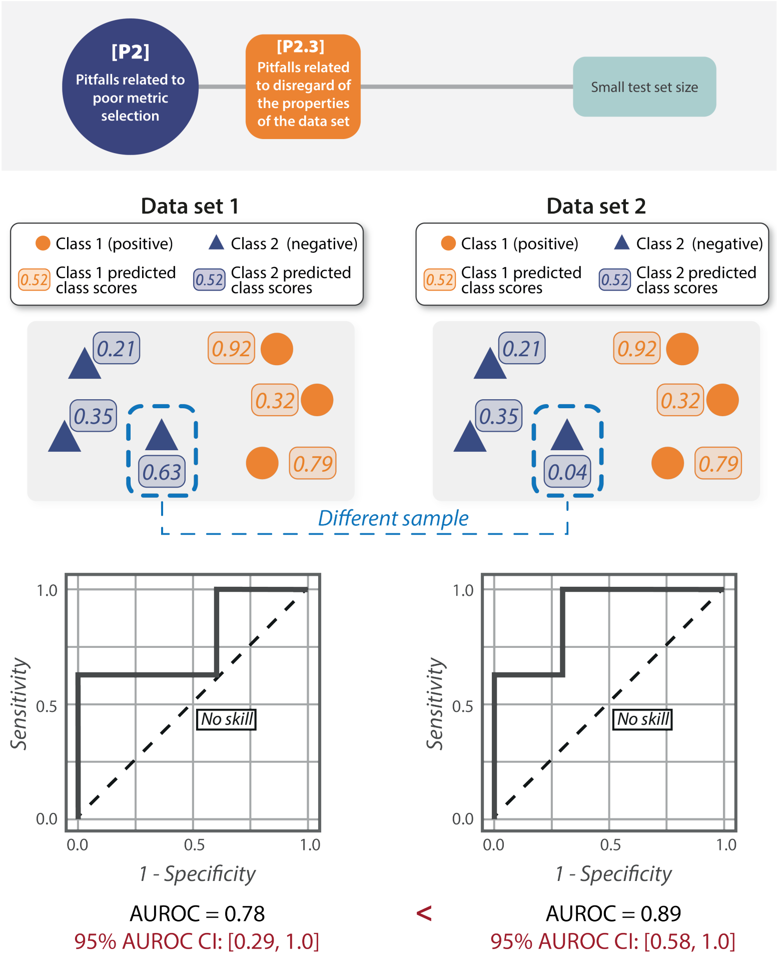

Fig. SN 2.16. Effect of calculating the Area under the Receiver Operating Characteristic Curve (AUROC) for very small sample sizes. The AUROC is very unstable for small sample sizes. *Data sets* 1 and 2 only contain six samples each, for which only one predicted score differs between sets. Drawing the Receiver Operating Characteristic (ROC) curve and calculating the AUROC leads to a large difference in scores between both data sets. The 95% Confidence Interval (CI) reveals that there is a large range of possible AUROC values. CIs were calculated based on [19]. This pitfall is also relevant for other counting metrics such as Accuracy, Average Precision (AP), Balanced Accuracy (BA), Expected Cost (EC), F_β Score, Free-Response Receiver Operating Characteristic (FROC) Score, Positive Likelihood Ratio (LR+), Matthews Correlation Coefficient (MCC), Net Benefit (NB), Negative Predictive Value (NPV), Positive Predictive Value (PPV), Sensitivity, Specificity, and Weighted Cohen's Kappa (WCK).

Common metrics do not account for inter-rater variability

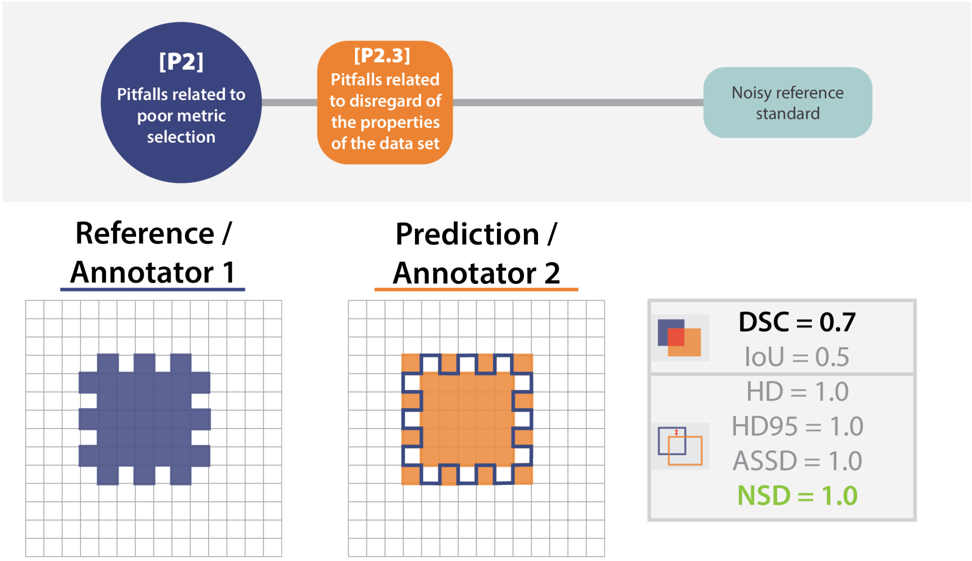

Fig. SN 2.17. Effect of inter-rater variability between two annotators. Assessing the performance of *Annotator 2* while using an reference annotation created by *Annotator 1* leads to a low Dice Similarity Coefficient (DSC) score because inter-rater variability is not taken into account by common overlap-based metrics. In contrast, the Normalized Surface Distance (NSD), applied with a threshold of $\tau = 1$, captures this variability. It should be noted, however, that this effect occurs primarily in small structures as overlap-based metrics tend to be robust to variations in the object boundaries in large structures. Further abbreviations: Intersection over Union (IoU), Hausdorff Distance (HD), Hausdorff Distance 95th Percentile (HD95), Average Symmetric Surface Distance (ASSD), Mean Average Surface Distance (MASD). This pitfall is also relevant for other boundary- and overlap-based metrics Boundary IoU, centerline Dice Similarity Coefficient (clDice), pixel-level F_β Score and Mean Average Surface Distance (MASD) and localization criteria such as Mask IoU > 0, Point inside Mask, Boundary IoU, IoR, and Mask IoU.

Empty reference or prediction leads to invalid scores

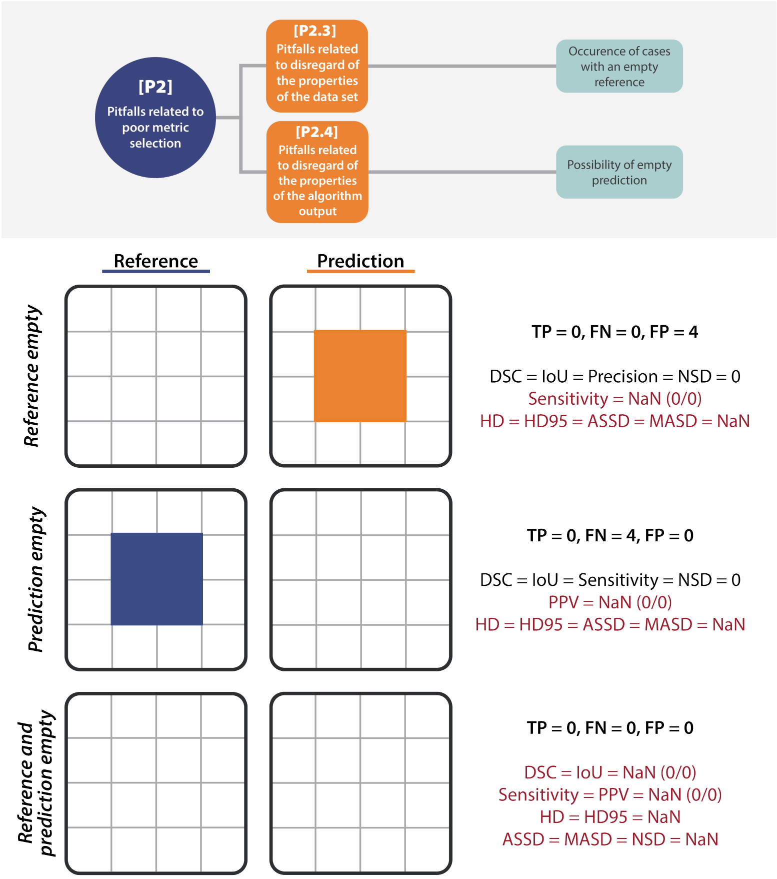

Fig. SN 2.18. Effect of empty references or predictions when applying common metrics per image (here for semantic segmentation). Empty images lead to division by zero for many common metrics as the numbers of the TPs, FPs, FNs turn zero. Used abbreviations: Average Symmetric Surface Distance (ASSD), Dice Similarity Coefficient (DSC), Hausdorff Distance (HD), Hausdorff Distance 95th Percentile (HD95), Intersection over Union (IoU), Mean Average Surface Distance (MASD), Not a Number (NaN), Normalized Surface Distance (NSD). This pitfall is also relevant for other boundary-based, overlap-based and counting metrics such as Boundary IoU, centerline Dice Similarity Coefficient (clDice), F_β Score, Negative Predictive Value (NPV), Positive Predictive Value (PPV), Sensitivity, and Specificity.

Common segmentation metrics are not well-suited for overlapping structures

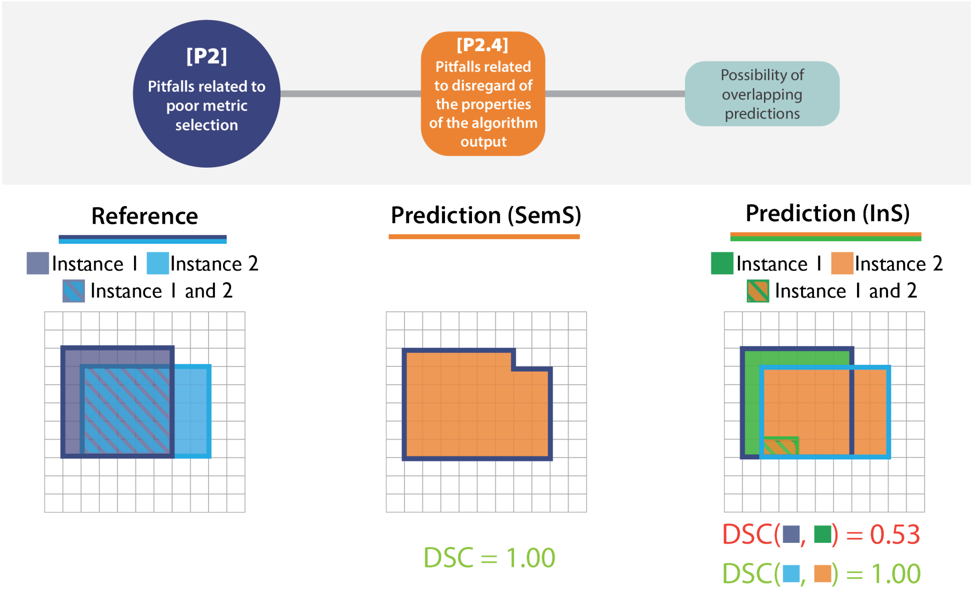

Fig. SN 2.19. Effect of overlapping predictions in segmentation problems. In semantic segmentation problem (SemS; right), overlapping predictions are merged into a single object, yielding a perfect metric score. Phrasing the problem as an instance segmentation problem reveals that the dark blue instance is not well-approximated at all. This issue is not revealed by common metrics if only semantic segmentation is performed (here: Dice Similarity Coefficient (DSC)). This pitfall is also relevant for other boundary- and overlap-based metrics such as Average Symmetric Surface Distance (ASSD), Boundary Intersection over Union (IoU), centerline Dice Similarity Coefficient (clDice), pixel-level F_β Score, Hausdorff Distance (HD), Hausdorff Distance 95th Percentile (HD95), IoU, Mean Average Surface Distance (MASD), and Normalized Surface Distance (NSD).

Selection of multi-threshold metrics in the absence of predicted class scores

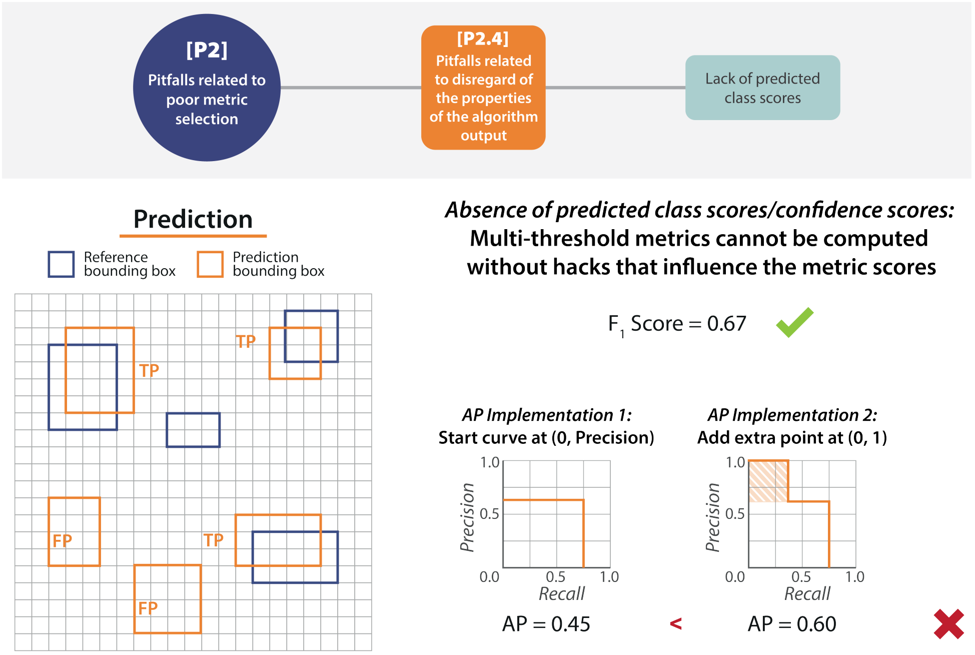

Fig. SN 2.20. Multi-threshold metrics should only be computed if predicted class scores are available, although an increasing body of work computes multi-threshold metrics such as AP in the absence of class scores (e.g., [3, 16, 25, 33, 39]). Otherwise, the strategy chosen for compensating the lack of class scores (here reflected by *Implementations 1* and *2*) leads to metric scores that are less well interpretable than those of established counting metrics working on a fixed confusion matrix (here: F_1 Score). This pitfall is also relevant for other multi-threshold metrics such as Area under the Receiver Operating Characteristic Curve (AUROC) and Free-Response Receiver Operating Characteristic (FROC) Score.

2.3 Pitfalls related to poor metric application

A data set typically contains several hundreds or thousands of images. When analyzing, aggregating and combining metric values, a number of factors need to be taken into account.

2.3.1 Pitfalls related to inadequate metric implementation. The implementation of metrics is, unfortunately, not standardized. While some metrics are straightforward to implement, others require more advanced techniques and offer a variety of implementation possibilities. Sources of metric implementation pitfalls include:

- Non-standardized definitions (Figs. 6a and SN 2.21)
- Discretization issues (Fig. SN 2.22)
- Sensitivity to hyperparameters (Fig. SN 2.23)
- Metric-specific issues (Fig. SN 2.24)

Lack of standardization leads to variation in metric scores

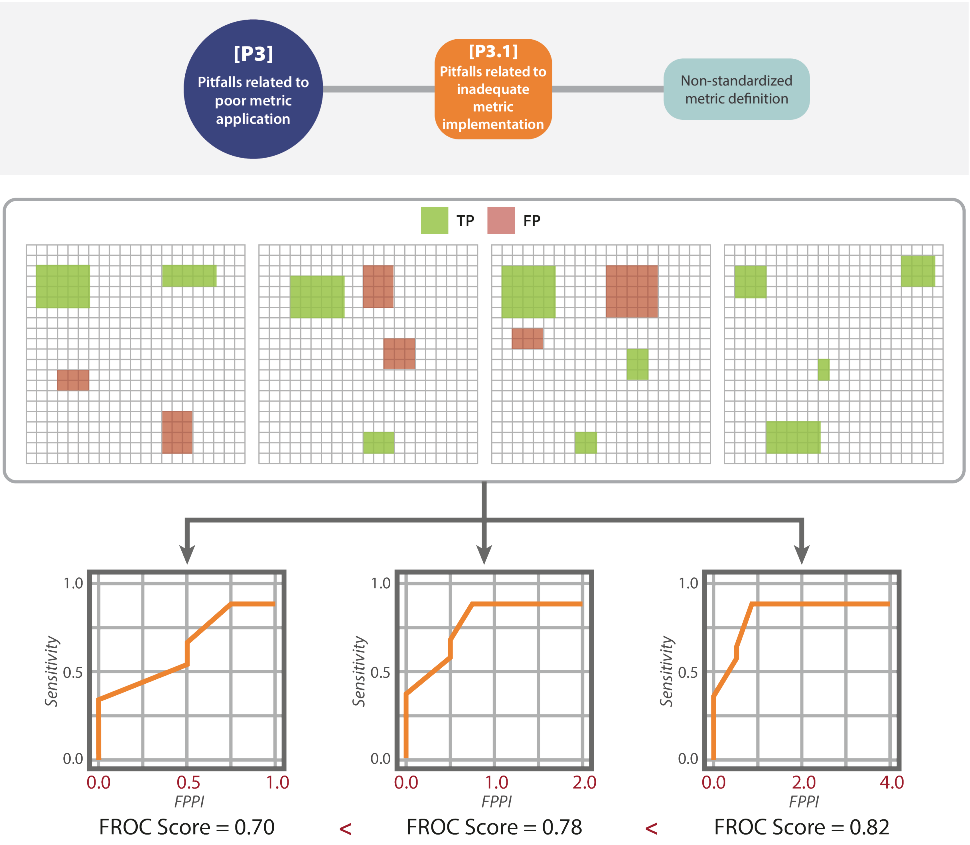

Fig. SN 2.21. Effect of defining different ranges for the False Positives per Image (FPPI) (which are unbounded to the top) used to draw the Free-Response Receiver Operating Characteristic (FROC) curve for the same prediction (top). The resulting FROC Scores differ for different boundaries of the x-axis used for the FPPI ([0, 1], [0, 2] and [0, 4]). Publications make use of different ranges for the x-axis, complicating comparison between works.

Common metrics suffer from discretization issues

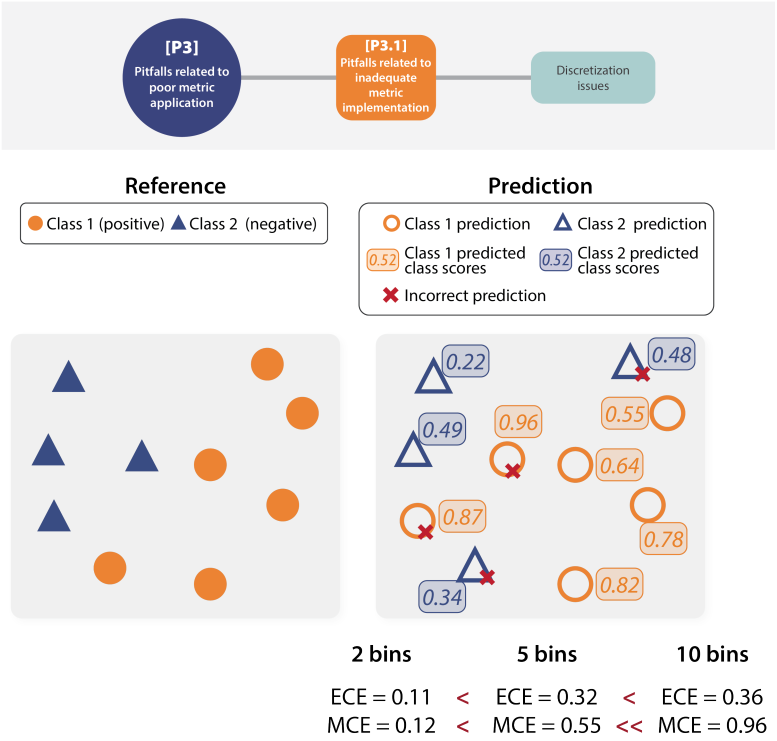

Fig. SN 2.22. Effect of choosing different bins for calculating the Expected Calibration Error (ECE) and Maximum Calibration Error (MCE). Three different strategies are chosen for the binning of the interval [0, 1] of the predicted class scores of the *Prediction*. The resulting metric scores are substantially affected by the number of bins [29].

Choice of hyperparameters may have largely impact metric scores

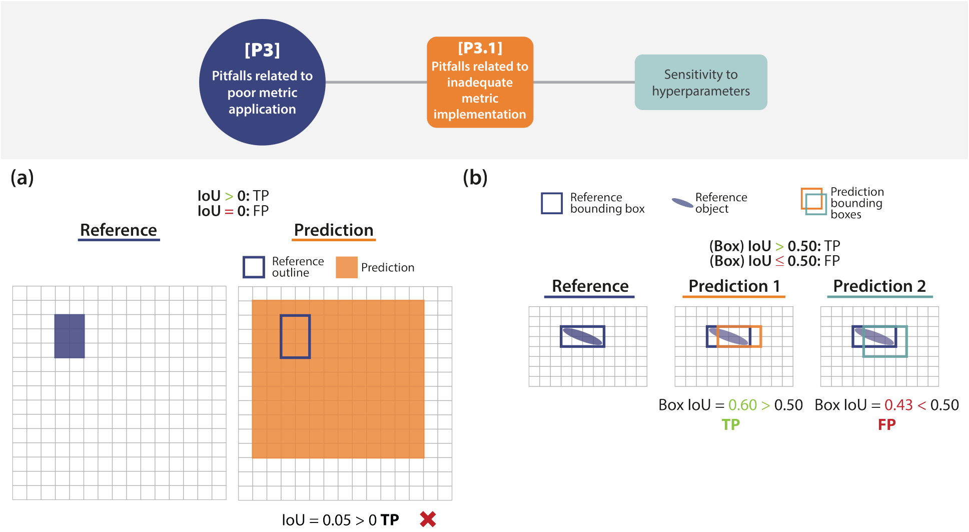

Fig. SN 2.23. Effect of the Intersection over Union (IoU) threshold on the localization (here Box IoU). **(a)** When defining a True Positive (TP) by a very loose IoU > 0, the resulting localizations may be deceived by very large predictions. **(b)** On the other hand, a strict IoU criterion may be problematic when the bounding box does not approximate the target structure shapes well. Although *Predictions 1* and *2* are very similar (differing in one pixel in one dimension), only *Prediction 1* is a TP because the number of bounding box pixels increases quadratically with the size of diagonal narrow structures. Further abbreviation: False Positive (FP).

Per-class tuning of the decision threshold yields misleading results

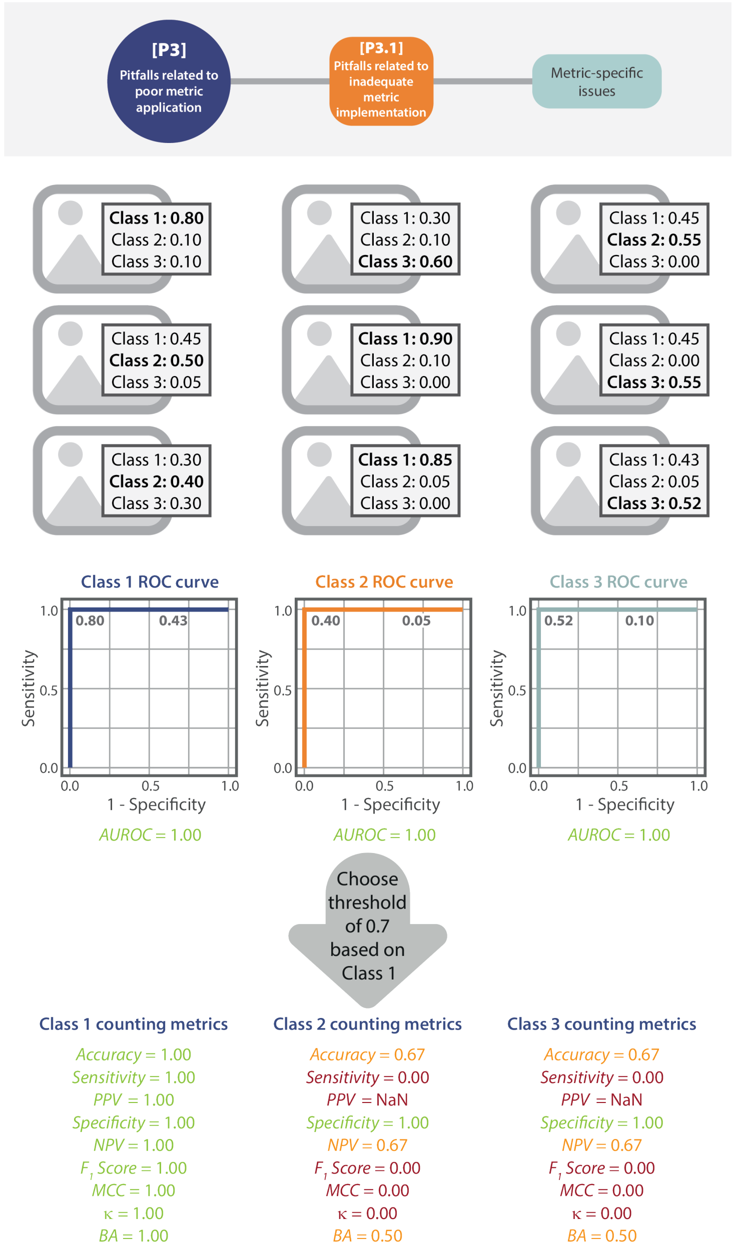

Fig. SN 2.24. Effect of the determination of a global threshold for all classes based on a single class. In a data set of three classes and nine images, the Area under the Receiver Operating Characteristic Curve (AUROC) score is 1.0 for every class. In practice, however, a global decision threshold needs to be set in multi-class problems, which typically renders substantially worse results. Here, the optimal threshold for *Class 1* yields poor results for *Classes* 2 and *3* (see e.g., [17, 37]). Used abbreviations: Positive Predictive Value (PPV), Negative Predictive Value (NPV), Matthews Correlation Coefficient (MCC), Cohen's Kappa κ, and Balanced Accuracy (BA).

2.3.2 Pitfalls related to inadequate metric aggregation. When aggregating metric values over multiple cases (data points), the method of metric aggregation should be clearly defined and reported including details for example on the aggregation operator (e.g., mean or median) and missing value handling. In addition, special care should be taken when aggregating across classes or different hierarchy levels. Pitfalls can be further subdivided into *class-related* and *data set-related* pitfalls. In the following, we present pitfalls stemming from the following sources:

Class-related pitfalls

- Hierarchical label structure (Fig. SN 2.25)
- Multi-class problem (Fig. SN 2.26)

Data set-related pitfalls

- Non-independence of test cases (Figs. 6b and SN 2.27)
- Risk of bias (Fig. SN 2.28)
- Possibility of invalid prediction (Fig. SN 2.29)

Standard aggregation schemes disregard hierarchical class structures

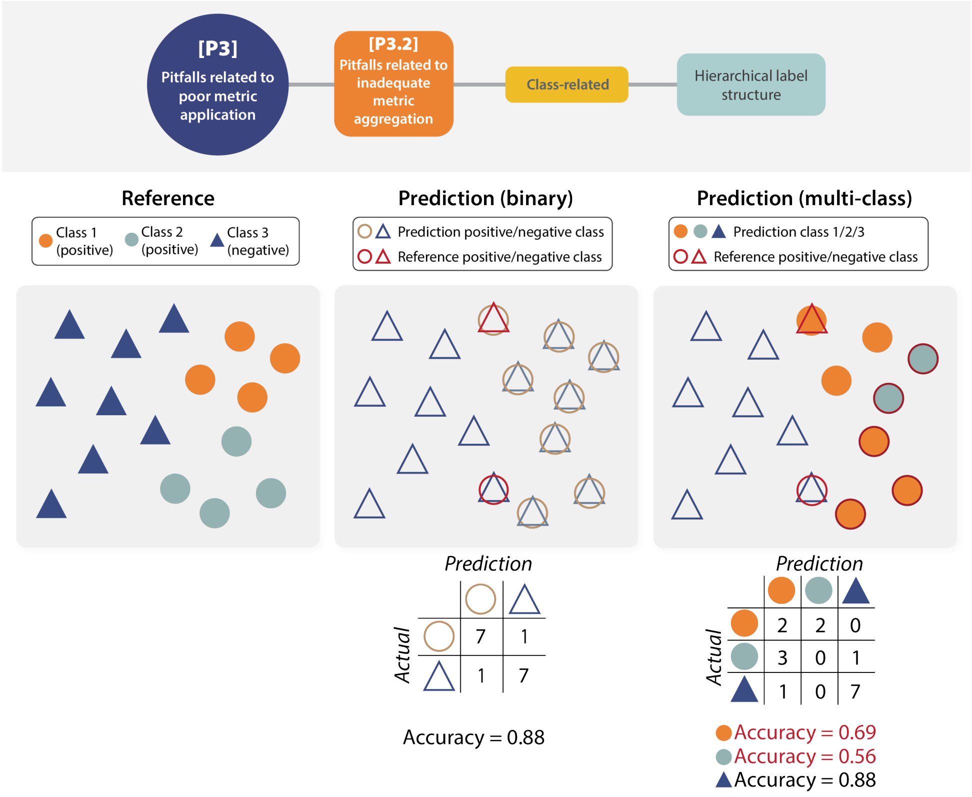

Fig. SN 2.25. Classes in categorical classification may be hierarchically structured, for example in the form of multiple positive classes and one negative class. The phrasing of the problem as binary *vs.* multi-class hugely affects the validation result. Binary classification (middle), differentiating triangles from circles, yields a good Accuracy, while per-class validation yields a poor score because the two circle classes cannot be distinguished well. Incorrect predictions are overlaid by a red shape of the correct reference class.

Lack of per-class validation conceals important information

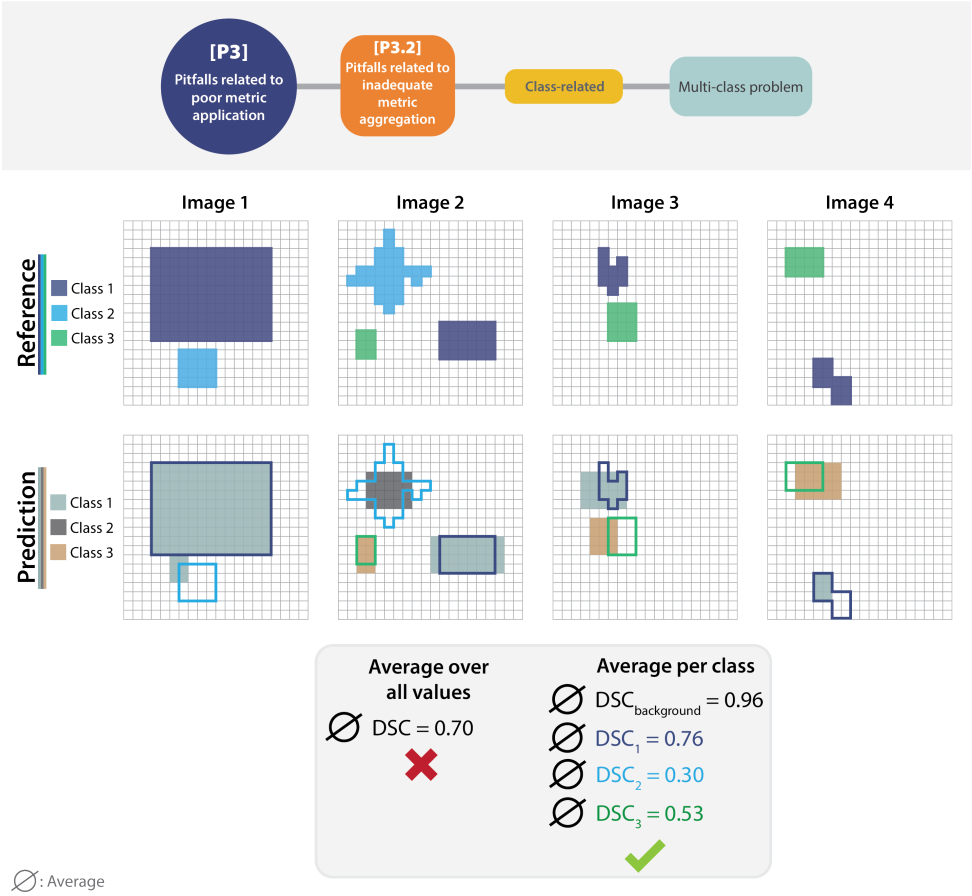

Fig. SN 2.26. Effect of ignoring the presence of multiple classes when aggregating metric values (here: using the mean). The overall average of all Dice Similarity Coefficient (DSC) scores for the four images is 0.7. Averaging per class reveals a very low performance for *Classes 2* and *3*. ∅ refers to the average DSC values.

Inter-class dependencies are concealed in standard aggregation schemes

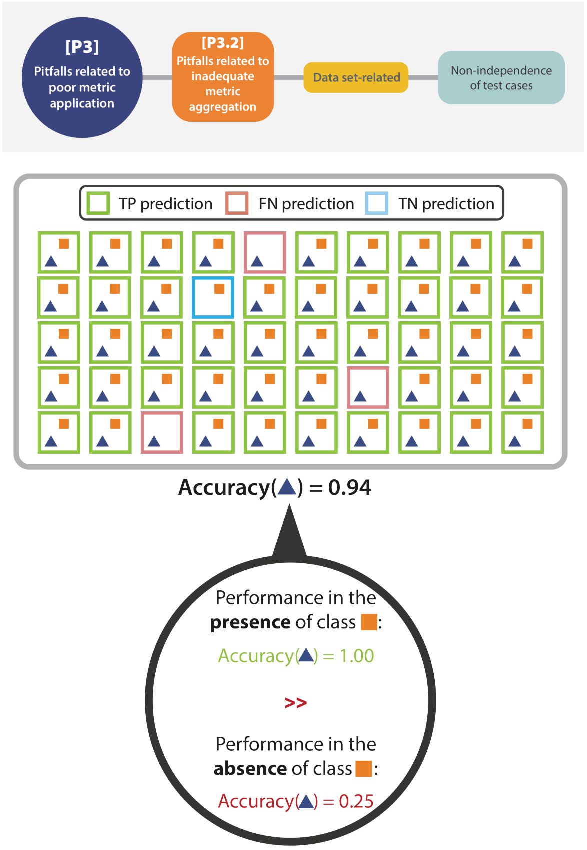

Fig. SN 2.27. Effect of interdependencies between classes. A prediction may show a near-perfect Accuracy score of 0.94 for the dark blue triangle as it frequently appears in conjunction with the orange square. By calculating the Accuracy in the *presence* and *absence* of the orange square class, it can be seen that the algorithm only works well in the presence of the orange square class.

Lack of stratification conceal biases

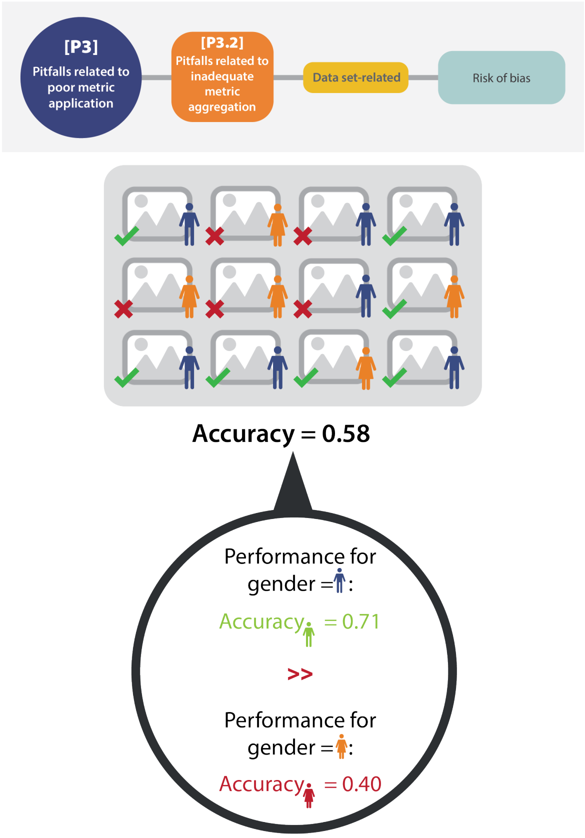

Fig. SN 2.28. Effect of disregarding relevant meta-information (here: gender). When ignoring the available meta-information of the patient's gender per image, any metric (here: *Accuracy*) fails to reveal that the algorithm performs much better for men compared to women. In this example, correct predictions are marked by a green check mark, incorrect predictions by a red cross.

Lack of missing data handling strategy yields misleading results

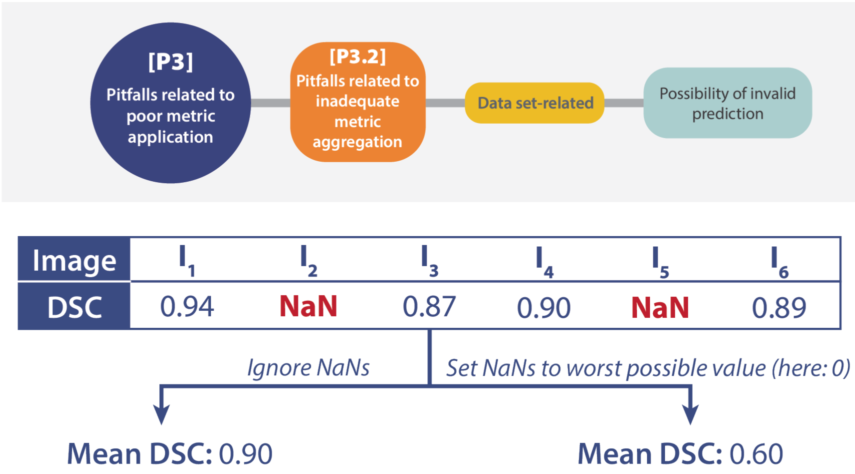

Fig. SN 2.29. Effect of invalid predictions (missing values) when aggregating metric values. In this example, ignoring missing values leads to a substantially higher Dice Similarity Coefficient (DSC)) compared to setting missing values to the worst possible value (here: 0).

2.3.3 Pitfalls related to inadequate ranking scheme. Rankings are often created to compare algorithm performances. In this context, we present pitfalls stemming from the following sources:

- Metric relationships (Fig. SN 2.30)
- Ranking uncertainty (Fig. SN 2.31)

Related metrics may yield identical rankings

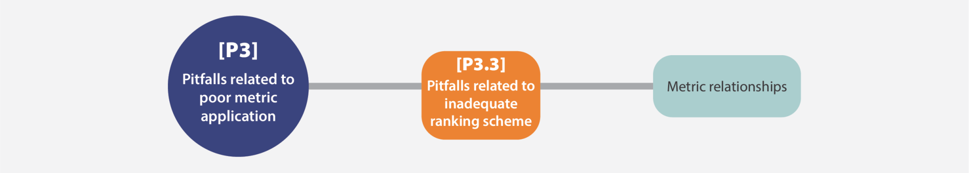

Raw metric values

Image	Algorithm	DSC	IoU	HD
Img_1	A1	0.60	0.43	1.41
	A2	0.58	0.41	2.24
	A3	0.27	0.10	6.08
	...	...	...	...
Img_n	A1	0.76	0.59	4.00
	A2	0.92	0.75	14.14
	A3	0.67	0.50	1.00

Rankings

Rank	Ranking 1: DSC	Ranking 2: IoU	Ranking 3: HD
1	A2	A2	A1
2	A1	A1	A3
3	A3	A3	A2

Fig. SN 2.30. Effect of using mathematically closely related metrics. The Dice Similarity Coefficient (DSC) and Intersection over Union (IoU) typically lead to the same ranking, whereas metrics from different families (here: Hausdorff Distance (HD)) may lead to substantially different rankings [58, 59]. Combining metrics that are related will not provide additional information for a ranking, and having multiple metrics measuring the same properties may overrule rankings of other properties (here: HD).

Ranking tables do not reflect ranking uncertainty

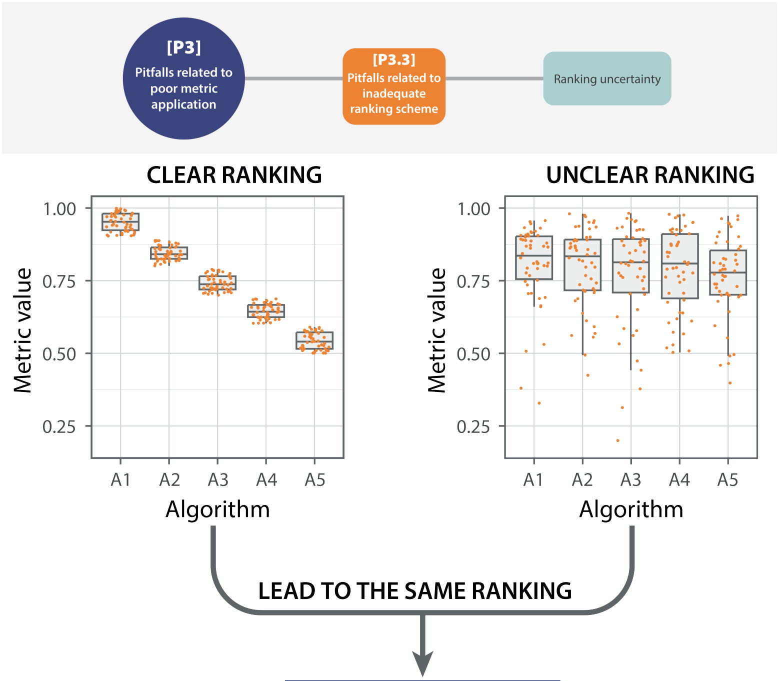

Rank	Algorithm
1	A2
2	A1
3	A3
4	A4
5	A5

Fig. SN 2.31. Effect of ranking uncertainty. The results of two benchmarking experiments with five algorithms *A1*-*A5* differ substantially, as shown by the boxplots of the metric values for every algorithm. While the left situation introduces a clear ranking visible from the boxplots, the right use case is not clear as performance is very similar across algorithms. However, both situations lead to the same ranking [43, 69]. Thus, solely providing ranking tables conceals information on ranking uncertainty.

2.3.4 Pitfalls related to inadequate metric reporting. A thorough reporting of metric values and aggregates is important both in terms of transparency and interpretability. However, several pitfalls are to be avoided in this regard. Sources of metric reporting pitfalls include:

- Non-determinism of algorithms (Fig. SN 2.32)
- Uninformative visualization (Figs. 6c and SN 2.33)

The non-determinism of neural networks effects metric results

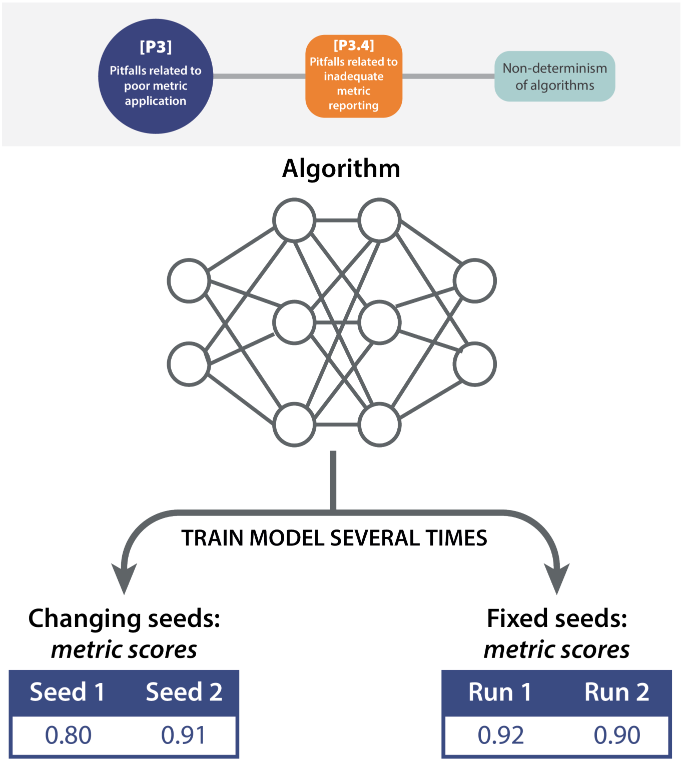

Fig. SN 2.32. Effect of non-determinism of artificial intelligence (AI) algorithms. An algorithm trained under identical conditions may yield different results when changing seeds (left), but also with fixed seeds (right). The latter may, for example, be caused by parallel processes, order of threads, auto-selection of primitive operations, and other factors [51][3]. Fixing seeds does not guarantee reproducibility even for the same hardware/software configuration as many software libraries have a degree of randomness on their operations.

[3]See for example: https://pytorch.org/docs/stable/notes/randomness.html

Common visualization schemes conceal relevant information

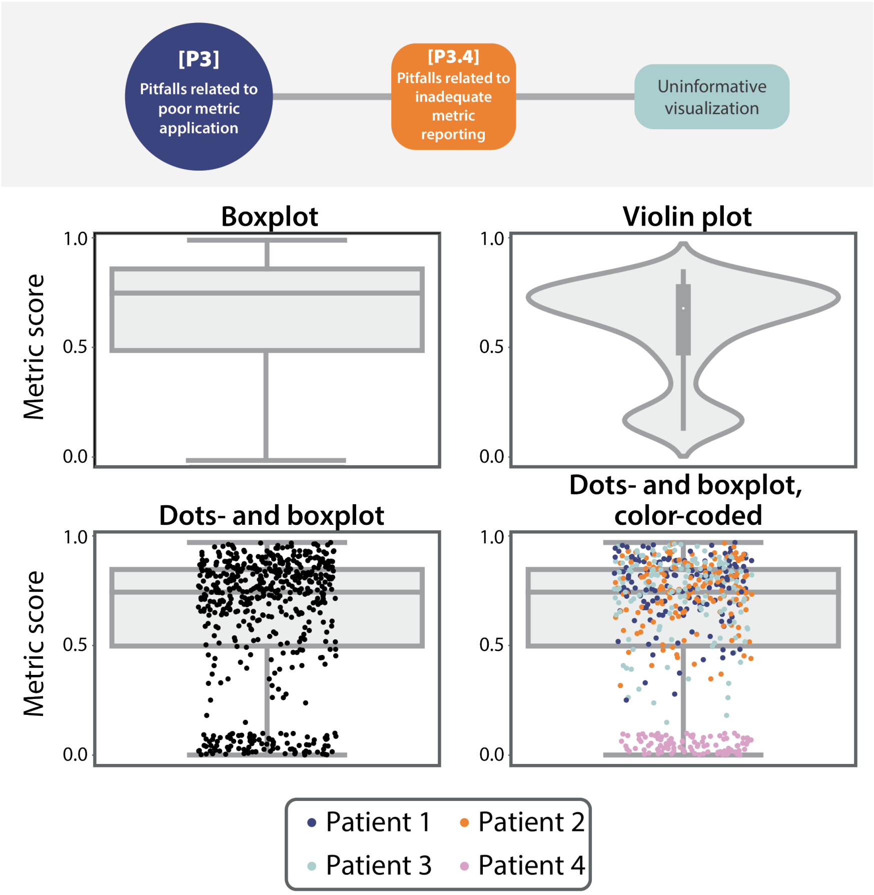

Fig. SN 2.33. Effect of different visualization types. A single boxplot (top left) does not provide sufficient information about the raw metric value distribution (here: Dice Similarity Coefficient (DSC)). Using a violin plot (top right) or adding the raw metric values as jittered dots on top (bottom left) adds important information. In the case of non-independent validation data, color/shape-coding helps reveal data clusters (bottom right).

2.3.5 Pitfalls related to inadequate interpretation of metric values. Interpreting metric scores and aggregates is an important step in algorithm performance analysis. However, several pitfalls can arise from interpretation. In the following, we present pitfalls related to:

- Low resolution (Fig. SN 2.34)
- Lack of upper/lower bounds (Fig. SN 2.35)
- Insufficient domain relevance of metric score differences (Fig. SN 2.36)

Image resolution affects metric scores

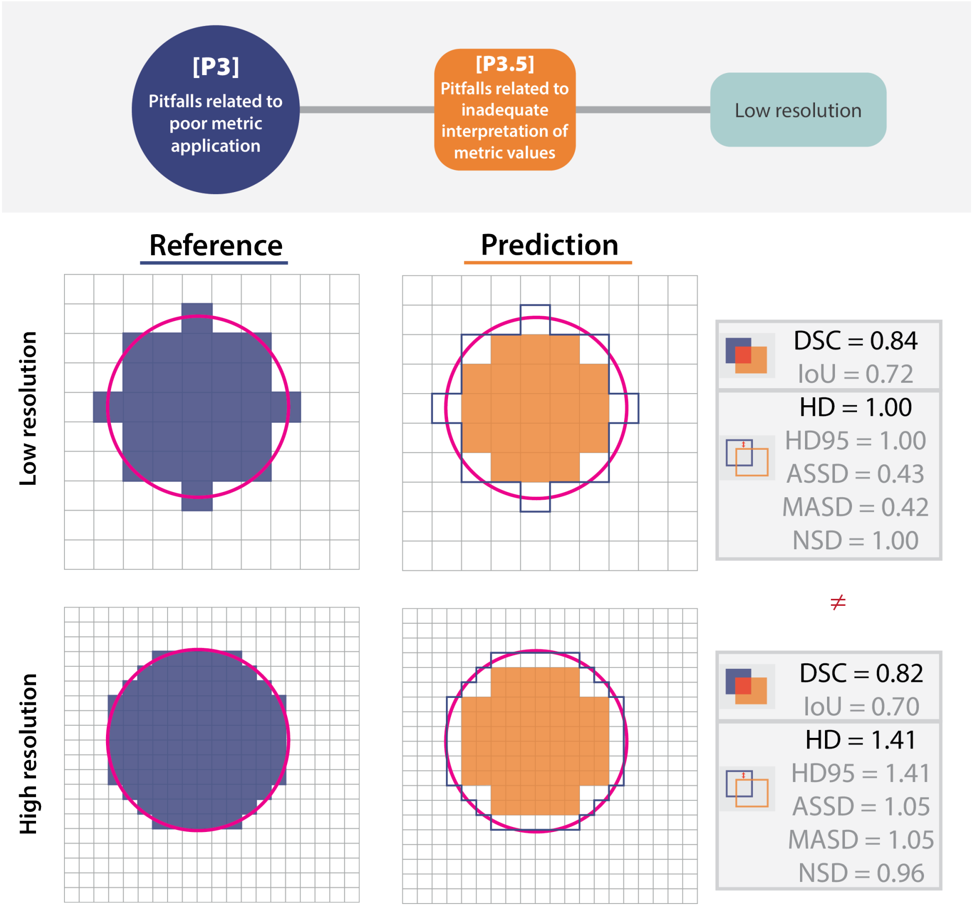

Fig. SN 2.34. Effect of different grid sizes. Differences in the grid size (resolution) of an image highly influence the image and the reference annotation (dark blue shape (reference) *vs.* pink outline (desired circle shape)), with a prediction of the exact same shape leading to different metric scores. Abbreviations: Dice Similarity Coefficient (DSC), Intersection over Union (IoU), Hausdorff Distance (HD), Hausdorff Distance 95th Percentile (HD95), Average Symmetric Surface Distance (ASSD), Mean Average Surface Distance (MASD), Normalized Surface Distance (NSD).

Lower bounds of metrics may not be achievable in practice

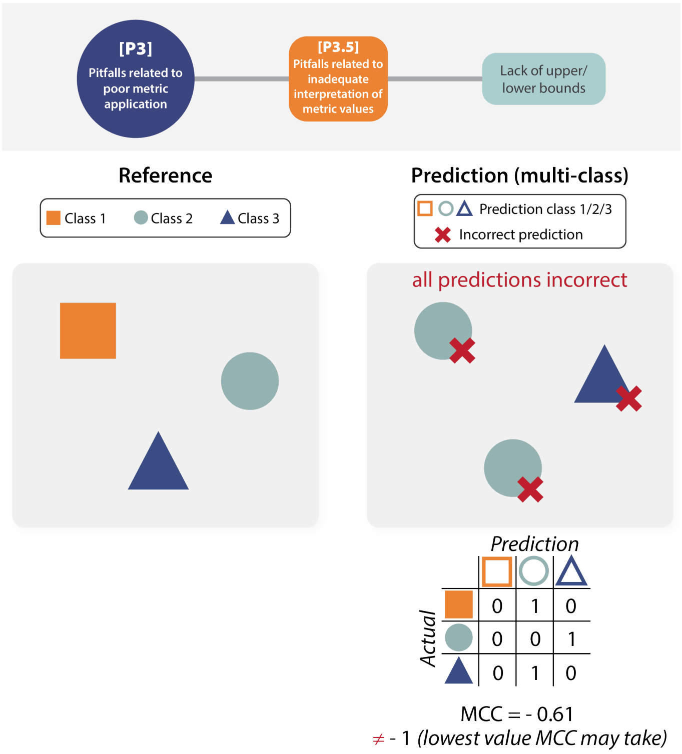

Fig. SN 2.35. Effect of theoretical bounds that may not be achievable in practice. In this multi-class example, all samples were predicted incorrectly. However, the theoretical lowest value for the Matthews Correlation Coefficient (MCC) metric (-1) cannot be achieved in this situation, rendering interpretation difficult.

Metric score differences leading to different rankings may be irrelevant

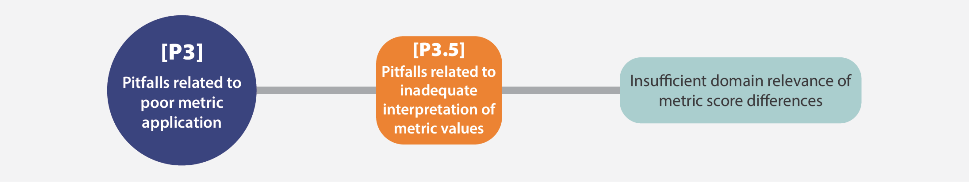

Rank	Algorithm	Aggregated metric score
1	A2	0.9543
2	A1	0.9542
3	A3	0.8703

Difference: 0.0001 ✗

Difference: 0.0839 ✓

Fig. SN 2.36. Effect of irrelevant metric score differences in rankings. The difference of the metric score aggregates of algorithms *A1* and *A2* is extremely low and not of biomedical relevance. However, the numerical difference would assign them different ranks.

SUPPL. NOTE 3 METRIC PROFILES

This section presents profiles for the metrics deemed particularly relevant by the *Metrics Reloaded* consortium [44]. For each metric, the respective description, formula, and value range (upward arrow: higher values better than lower values; downward arrow: lower values are better than higher values) are provided, along with further important characteristics, such as the used cardinalities of a confusion matrix, or potential prevalence dependency. Finally, relevant pitfalls are highlighted. Many of the presented metrics rely on the confusion matrix, which is illustrated in Fig. SN 3.37.

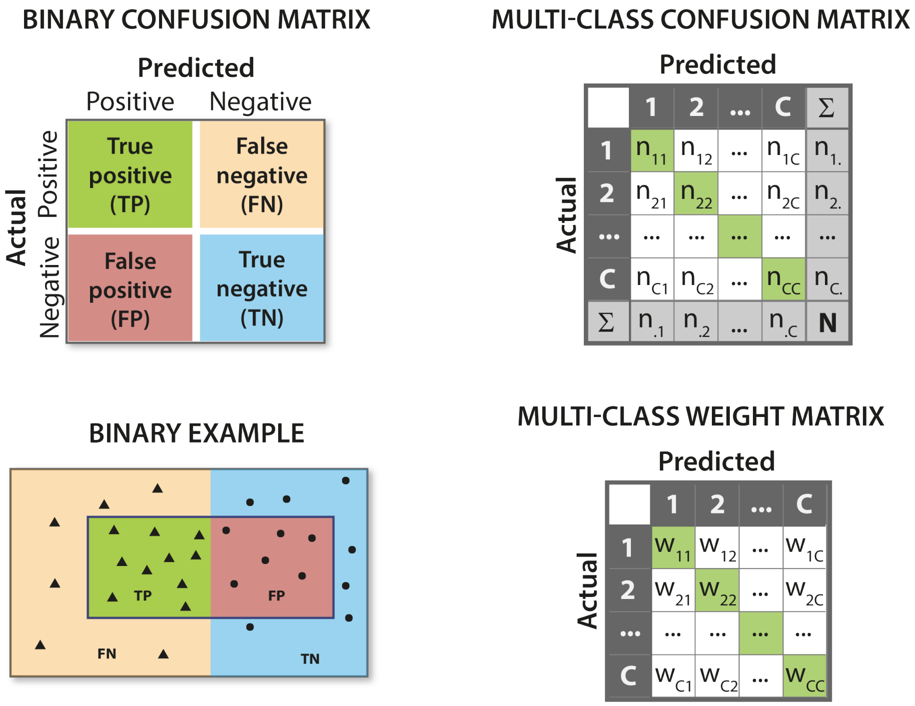

Fig. SN 3.37. Schematic example of the confusion matrix for two and for C classes. For the latter case, we also present a weight or cost matrix with weights $w_{ij} > 0$ without loss of generality. For the binary confusion matrix, we show an example illustrating the cardinalities for a prediction of triangles and circles.

3.1 Discrimination metrics

3.1.1 Counting metrics. +

ACCURACY

$$\text{Accuracy} = \frac{TP + TN}{TP + TN + FP + FN} =$$

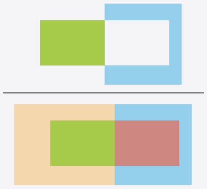

VALUE RANGE: [0, 1] ↑

DESCRIPTION

Accuracy measures the ratio of samples that were correctly classified over all predictions made.

DEFINITION

[Tharwat, 2020]

CARDINALITIES

TP FP FN TN

METRIC FAMILY

Counting metric | Multi-threshold metric | Distance-based metric

MULTI-CLASS DEFINITION

For C classes, Accuracy is defined as:

$$\text{Accuracy} = \frac{1}{N}\sum_{i=1}^{C} n_{ii}$$

n_{ii}: *diagonal entries of the confusion matrix; sum equals number of correctly classified samples*

N: total number of samples

PREVALENCE DEPENDENCY

RELEVANT PITFALLS

- Accuracy is highly sensitive to class imbalance (Figs. 5a, SN 2.15).
- Accuracy is prevalence-dependent, thus not comparable across data sets with different prevalences (Fig. SN 2.7).
- Accuracy does not allow for incorporating unequal severity of confusions across classes. This implies that the metric is not applicable to cost-benefit analyses (Fig. SN 2.9), or when target classes are related on an ordinal scale (Fig. 4b).
- Accuracy depends on the definition of TN (undefined for ObD and InS).

Fig. SN 3.38. Metric profile of Accuracy. The upward arrow in the value range indicates that higher values are better than lower values. Abbreviations: False Negative (FN), False Positive (FP), Instance Segmentation (InS), Object Detection (ObD), True Negative (TN), True Positive (TP). Reference: Tharwat, 2020: [60]. Mentioned figures: Figs. 4b, 5a, SN 2.7, SN 2.9, SN 2.15.

BALANCED ACCURACY (BA)

$$BA = \frac{1}{2}(\text{Sensitivity} + \text{Specificity}) = \frac{1}{2}\Big($$ 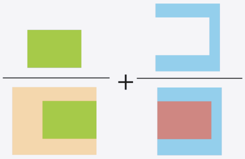 $$\Big)$$

VALUE RANGE: [0, 1] ↑

DESCRIPTION

BA measures the arithmetic mean of Sensitivities for each class, i.e., for each class, it measures the fraction of actual positive samples that were predicted as such.

DEFINITION

[Tharwat, 2020]

CARDINALITIES

TP	FP	FN	TN
●	●	●	●

PREVALENCE DEPENDENCY ○

MULTI-CLASS DEFINITION

For C classes, BA is defined as the arithmetic mean of Sensitivities per class:

$$BA = \frac{1}{C}\sum_{i=1}^{C} \text{Sensitivity}_i = \frac{1}{C}\sum_{i=1}^{C} \frac{n_{ii}}{n_{i\cdot}}$$

n_{ii}: *diagonal entries of the confusion matrix; sum equals number of correctly classified samples*

$n_{i\cdot}$: *sum of entries of row i in the confusion matrix*

METRIC FAMILY

Counting metric	Multi-threshold metric	Distance-based metric
●	○	○

RELEVANT PITFALLS

- BA can be misleading for imbalanced situations (Fig. 5a).
- BA does not allow for incorporating unequal severity of confusions across classes. This implies that the metric is not applicable to cost-benefit analyses (Fig. SN 2.9), or when target classes are related on an ordinal scale (Fig. 4b).
- BA is not well-suited if an unequal treatment of classes is requested (e.g., some classes are treated as more important than others) [Grandini et al., 2020].
- BA is insensitive to changes in predictive values (PPV and NPV) [Maier-Hein et al., 2022].
- In binary tasks, BA may yield the same value for different Sensitivity and Specificity scores [Reinke et al., 2021].
- BA depends on the definition of TN (undefined for ObD and InS).

Fig. SN 3.39. Metric profile of Balanced Accuracy (BA). The upward arrow in the value range indicates that higher values are better than lower values. Abbreviations: False Negative (FN), False Positive (FP), Instance Segmentation (InS), Object Detection (ObD), Positive Predictive Value (PPV), True Negative (TN), True Positive (TP). References: Grandini et al., 2020: [27], Maier-Hein et al., 2022: [44], Reinke et al., 2021: [54], Tharwat, 2020: [60]. Mentioned figures: Figs. 4b, 5a, SN 2.9.

CENTERLINE DICE SIMILARITY COEFFICIENT (clDICE)

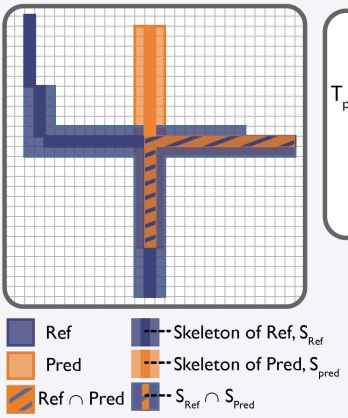

Topology precision

$$T_{prec}(S_{Pred}, Ref) = \frac{|S_{Pred} \cap Ref|}{|S_{Pred}|} =$$

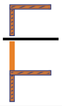

Topology sensitivity

$$T_{sens}(S_{Ref}, Pred) = \frac{|S_{Ref} \cap Pred|}{|S_{Ref}|} =$$

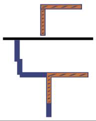

$$clDice(Ref,Pred) = \frac{2 \cdot T_{sens}(S_{Ref}, Pred) \cdot T_{prec}(S_{Pred}, Ref)}{T_{sens}(S_{Ref}, Pred) + T_{prec}(S_{Pred}, Ref)}$$

VALUE RANGE: [0, 1] ↑

DESCRIPTION

clDice measures the overlap between two structures, ideally tubular-shaped. The formula is similar to the DSC, but relies on the *topology precision* and *topology sensitivity* which are defined based on the skeletons of the structures.

DEFINITION

[Shit et al., 2021]

CARDINALITIES

TP	FP	FN	TN
●	●	●	○

METRIC FAMILY

Counting metric	Multi-threshold metric	Distance-based metric
●	○	○

RELEVANT PITFALLS

- clDice penalizes missed pixels more in small objects (Fig. SN 2.10, SN 2.11, Extended Data Fig. 1a).
- clDice treats oversegmentation and undersegmentation differently (Fig. SN 2.8).
- clDice does not compensate for inter-rater variability (Fig. SN 2.17).
- clDice is undefined if both reference and prediction are empty, and ad hoc rules need to be established depending on the use case (Fig. SN 2.18).
- clDice ignores the general overlap and volume of structures.
- clDice is sensitive to the method of skeleton extraction.

Fig. SN 3.40. Metric profile of centerline Dice Similarity Coefficient (clDice). The upward arrow in the value range indicates that higher values are better than lower values. Abbreviations: False Negative (FN), False Positive (FP), True Negative (TN), True Positive (TP). Reference: Shit et al., 2021: [57]. Mentioned figures: Extended Data Fig. 1a, Figs. SN 2.8, SN 2.10, SN 2.11, SN 2.17, SN 2.18.

DICE SIMILARITY COEFFICIENT (DSC)

Synonyms: Dice, Dice Coefficient, Sørensen–Dice Coefficient, F_1 Score, Balanced F Score

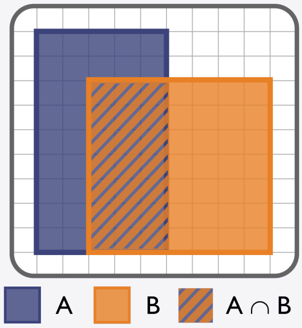

$$DSC(A,B) = \frac{2\,|A \cap B|}{|A| + |B|} = \frac{2\,PPV \cdot Sensitivity}{PPV + Sensitivity}$$

VALUE RANGE: [0, 1] ↑

DESCRIPTION
DSC measures the overlap between two structures.

DEFINITION
[Dice, 1945]

CARDINALITIES

TP	FP	FN	TN
●	●	●	○

METRIC FAMILY

Counting metric	Multi-threshold metric	Distance-based metric
●	○	○

RELEVANT PITFALLS
- DSC is unaware of shapes, distances and centers (Figs. 4a, SN 2.5, SN 2.12, Extended Data Fig. 1b).
- DSC penalizes missed pixels more in small objects (Figs. SN 2.10, SN 2.12, Extended Data Fig. 1a).
- DSC is undefined if both the reference and prediction are empty, and ad hoc rules need to be established depending on the use case (Fig. SN 2.18).
- DSC treats oversegmentation and undersegmentation differently (Fig. SN 2.8).
- DSC does not compensate for inter-rater variability (Fig. SN 2.17).
- DSC behaves differently in 2D and 3D settings. In 3D settings, the additional z-dimension results in a cubical increase in erroneous pixels [Reinke et al., 2021].

Fig. SN 3.41. Metric profile of Dice Similarity Coefficient (DSC). The upward arrow in the value range indicates that higher values are better than lower values. Abbreviations: False Negative (FN), False Positive (FP), Positive Predictive Value (PPV), True Negative (TN), True Positive (TP). References: Dice, 1945: [20], Reinke et al., 2021: [54]. Mentioned figures: Figs. 4a, SN 2.5, SN 2.8, SN 2.10, SN 2.11, SN 2.12, SN 2.17, SN 2.18, Extended Data Fig. 1a-b.

EXPECTED COST (EC)/NORMALIZED EC (ECN)

Synonyms: Expected prediction error, Expected loss

$$EC = w_{miss} \cdot \underbrace{\frac{FN}{TP+FN}}_{P_{miss}} \cdot \underbrace{\frac{TP+FN}{TP+TN+FP+FN}}_{P_{tar}} + w_{FA} \cdot \underbrace{\frac{FP}{TN+FP}}_{P_{FA}} \cdot 1 - \underbrace{\frac{TP+FN}{TP+TN+FP+FN}}_{P_{tar}}$$

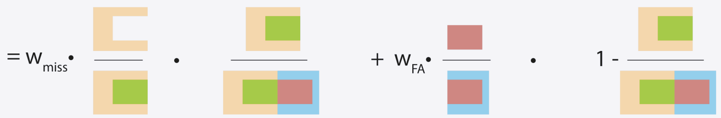

P_{miss}: FN (miss) rate, P_{FA}: FP (false alarm) rate
P_{tar}: prior probability (prevalence)
w_{miss}/w_{FA}: (estimation of) costs of the respective errors; can be adjusted as a weighting of them.

VALUE RANGE: [0, ∞) ↓
EC can be assumed to be positive if costs are non-negative, which can be done without loss of generality.

DESCRIPTION

EC is a generalization of the probability of error (which is, in turn, 1 - Accuracy) for cases in which errors cannot all be considered to have equally severe consequences. It is defined as the expectation of the cost, where the cost incurred on a certain sample depends on the sample's class and the decision made for that sample. In practice, the expectation can be estimated as a simple average of the costs over the evaluation samples.
EC describes the weighted sum of error rates. It can be used to measure discrimination and calibration in one score.

VARIANT

Normalized EC (ECN): normalizes EC by the EC of a naive system.

DEFINITION

[Bishop and Nasrabadi, 2006; Hastie et al., 2009; Ferrer, 2022]

CARDINALITIES

TP	FP	FN	TN
●	●	●	●

PREVALENCE DEPENDENCY? ◐

Both options are possible depending on how the priors are set in the definition of the metric.

METRIC FAMILY

Counting metric	Multi-threshold metric	Distance-based metric
●	○	○

MULTI-CLASS DEFINITION

For C classes, EC is defined as:

$$EC = \sum_{i=1}^{C} \sum_{j=1}^{C} P_i \cdot w_{ij} \cdot \frac{n_{ij}}{n_{i\cdot}}$$

n_{ij}: entry of the confusion matrix for row i and column j, i.e., samples of actual class i that have been predicted as class j
$n_{i\cdot}$: sum of entries of row i of the confusion matrix
w_{ij}: costs for the entry of the confusion matrix for row i and column j, i.e., the cost for predicting a sample of actual class i that was predicted as class j
P_i: prevalence of class i; usually ($n_{i\cdot}$ / N), but in some cases one might want to plug in P_i directly from a target application

RELEVANT PITFALLS

- EC is rather uncommon and can therefore not be used for comparison with other publications.
- EC can be close to optimal even for poor predictive values (PPV and NPV) [Maier-Hein et al., 2022]
- ECN cannot be configured to ensure equal class contribution without losing its ability to ensure high predictive values [Maier-Hein et al., 2022].

Fig. SN 3.42. Metric profile of Expected Cost (EC). The downward arrow in the value range indicates that lower values are better than higher values. Abbreviations: False Negative (FN), False Positive (FP), True Negative (TN), True Positive (TP). References: Bishop and Nasrabadi, 2006: [7], Ferrer 2022: [24], Hastie et al., 2009: [32], Maier-Hein et al., 2022: [44].

F_β SCORE

$$F_\beta\ \text{Score} = (1+\beta^2)\frac{\text{Precision} \cdot \text{Sensitivity}}{\beta^2 \cdot \text{Precision} + \text{Sensitivity}}$$

$$= \frac{(1+\beta^2) \cdot TP}{(1+\beta^2) \cdot TP + \beta^2 \cdot FN + FP} =$$

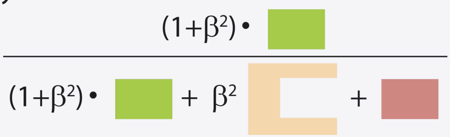

VALUE RANGE: [0, 1] ↑

DESCRIPTION

The F_β Score weights PPV (FP) and Sensitivity (FN) with the parameter β.

The special case of $\beta = 1$ is the harmonic mean of PPV and Sensitivity and is a common metric in segmentation problems (here usually referred to as DSC). In segmentation problems, F_β Score weights the penalization of oversegmentation (FP) and undersegmentation (FN) with the parameter β.

DEFINITION

[Van Rijsbergen, 1979; Chinchor 1992]

CARDINALITIES

TP	FP	FN	TN
●	●	●	○

PREVALENCE DEPENDENCY ●

METRIC FAMILY

Counting metric	Multi-threshold metric	Distance-based metric
●	○	○

RELEVANT PITFALLS

F_β Score for classification/detection assessment:

- F_β Score is prevalence-dependent, thus not comparable across data sets with different prevalences (Figs. SN 2.7, SN 2.15).
- Compared to other per-class counting metrics (e.g., LR+) it lacks the interpretability with respect to a naive classifier
- F_β Score depends on the definition of the positive class [Reinke et al., 2021].

F_β Score for segmentation assessment:

- F_β Score is unaware of the structure shape and center (Figs. 4a, SN 2.5, SN 2.12, Extended Data Fig. 1b).
- F_β Score penalizes missed pixels more in small objects (Fig. SN 2.10, Extended Data Fig. 1a).
- F_β Score does not compensate for inter-rater variability (Fig. 2.17).
- F_β Score behaves differently in 2D and 3D settings. In 3D settings, the additional z-dimension results in a cubical increase in erroneous pixels [Reinke et al., 2021].

F_β Score is undefined if both reference and prediction are empty, and ad hoc rules need to be established depending on the use case (Fig. SN 2.18, Extended Data Fig. 2b).

Fig. SN 3.43. Metric profile of F_β Score.[12, 63]. The upward arrow in the value range indicates that higher values are better than lower values. Abbreviations: Dice Similarity Coefficient (DSC), False Negative (FN), False Positive (FP), Positive Predictive Value (PPV), True Negative (TN), True Positive (TP).References: Chinchor 1992: [12], Reinke et al., 2021: [54], Van Rijsbergen, 1979: [63]. Mentioned figures: Figs. 4a, SN 2.5, SN 2.7, SN 2.10, SN 2.12, SN 2.15, SN 2.17, SN 2.18, Extended Data Figs. 1a-b and 2b.

FALSE POSITIVES PER IMAGE (FPPI)

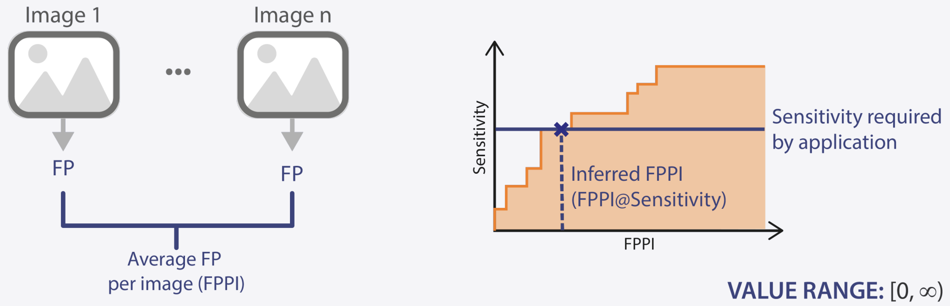

VALUE RANGE: [0, ∞) ↑

DESCRIPTION

FPPI measures the number of FPs per image. It was originally proposed for the calculation of the FROC Score. While not yet standardized, FPPI could also be used as a metric of its own for a given value of Sensitivity, derived from the FROC curve.

DEFINITION

[Van Ginneken et al., 2010; Bandos et al., 2009]

CARDINALITIES

TP	FP	FN	TN
○	●	○	○

PREVALENCE DEPENDENCY ○

METRIC FAMILY

Counting metric	Multi-threshold metric	Distance-based metric
●	○	○

RELEVANT PITFALLS

- FPPI is not bounded between 0 and 1 (Fig. SN 2.21).
- FPPI depends on the number of images, which can hide performance differences if many images are present [Reinke et al., 2021].
- FFPI only measures a single entity (FPs) of the confusion matrix and and should just be used in combination with other metrics (as done in the FROC Score).

Fig. SN 3.44. Metric profile of False Positives per Image (FPPI). The upward arrow in the value range indicates that higher values are better than lower values. Abbreviations: False Negative (FN), False Positive (FP), Free-Response Receiver Operating Characteristic (FROC), True Negative (TN), True Positive (TP). References: Bandos et al., 2009: [5], Reinke et al., 2021: [54], Van Ginneken et al., 2010: [62]. Mentioned figure: Fig. SN 2.21.

INTERSECTION OVER UNION (IoU)

Synonyms: Jaccard Index, Tanimoto Coefficient

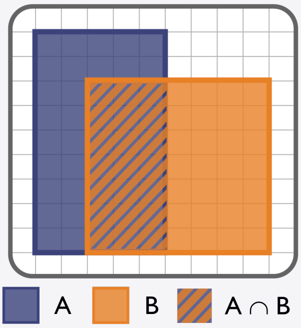

IoU(A,B) = 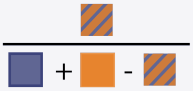

$$= \frac{|A \cap B|}{|A| + |B| - |A \cap B|} = \frac{|A \cap B|}{|A \cup B|}$$

$$= \frac{\text{PPV} \cdot \text{Sensitivity}}{\text{PPV} + \text{Sensitvity} - \text{PPV} \cdot \text{Sensitivity}}$$

VALUE RANGE: [0, 1] ↑

DESCRIPTION

IoU measures the overlap between two structures. It is often referred to as **Box IoU** when comparing bounding boxes, **Mask IoU** when comparing segmentation masks, or **Approx IoU** when comparing approximations of objects beyond bounding boxes.

DEFINITION

[Jaccard, 1912]

CARDINALITIES

TP	FP	FN	TN
●	●	●	○

METRIC FAMILY

Counting metric	Multi-threshold metric	Distance-based metric
●	○	○

RELEVANT PITFALLS

- IoU is unaware of shapes, boundaries, distances and centers (Figs. 4a, SN 2.5, SN 2.12, Extended Data Fig. 1b).
- IoU penalizes missed pixels more in small objects (Figs. SN 2.10, SN 2.11, Extended Data Fig. 1a).
- IoU is undefined if both the reference and prediction are empty, and ad hoc rules need to be established depending on the use case (Fig. SN 2.18).
- IoU treats oversegmentation and undersegmentation differently (Fig. SN 2.8).
- IoU does not compensate for inter-rater variability (Fig. SN 2.17).
- IoU behaves differently in 2D and 3D settings. In 3D settings, the additional z-dimension results in a cubical increase in erroneous pixels [Reinke et al., 2021].

Fig. SN 3.45. Metric profile of Intersection over Union (IoU). The upward arrow in the value range indicates that higher values are better than lower values. Abbreviations: False Negative (FN), False Positive (FP), Positive Predictive Value (PPV), True Negative (TN), True Positive (TP). References: Jaccard, 1912: [35], Reinke et al., 2021: [54]. Mentioned figures: Figs. 4a, SN 2.5, SN 2.8, SN 2.10, SN 2.11, SN 2.12, SN 2.17, SN 2.18, Extended Data Fig. 1a-b.

MATTHEWS CORRELATION COEFFICIENT (MCC)

Synonym: Phi Coefficient

$$MCC = \frac{TP \cdot TN - FP \cdot FN}{\sqrt{(TP + FP)(TP + FN)(TN + FP)(TN + FN)}} =$$

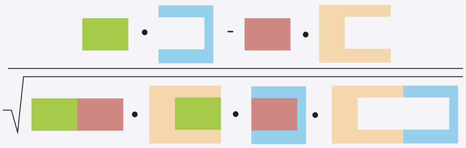

VALUE RANGE: [-1, 1] ↑

A value of 0 refers to a prediction which is not better than random guessing.

DESCRIPTION

MCC measures the correlation between the actual and the predicted class.

DEFINITION

[Matthews, 1975]

CARDINALITIES

TP	FP	FN	TN
●	●	●	●

METRIC FAMILY

Counting metric	Multi-threshold metric	Distance-based metric
●	○	○

PREVALENCE DEPENDENCY ●

MULTI-CLASS DEFINITION

For C classes, MCC can be defined as:

$$MCC = \frac{\sum_{i=1}^{C} \sum_{j=1}^{C} \sum_{k=1}^{C} n_{ii} \cdot n_{jk} - n_{ij} \cdot n_{ki}}{\sqrt{\sum_{i=1}^{C} \left(\sum_{j=1}^{C} n_{ij}\right)\left(\sum_{i'|i' \neq i} \sum_{j'=1}^{C} n_{i'j'}\right)} \sqrt{\sum_{i=1}^{C} \left(\sum_{j=1}^{C} n_{ji}\right)\left(\sum_{i'|i' \neq i} \sum_{j'=1}^{C} n_{j'i'}\right)}}$$

n_{ij}: *entry of the confusion matrix for row i and column j, i.e., samples of actual class i that were predicted as class j*

RELEVANT PITFALLS

- MCC is prevalence-dependent, thus not comparable across data sets with different prevalences (Fig. SN 2.7, [Reinke et al., 2021]).
- MCC does not allow for incorporating unequal severity of confusions across classes. This implies that the metric is not applicable to cost-benefit analyses (Fig. SN 2.9), or when target classes are related on an ordinal scale (Fig. 4b).
- The theoretical lower bound of MCC (-1) may not always be achievable (Fig. SN 2.35).
- MCC is hard to interpret [Zhu 2020].
- Compared to other metrics like EC, MCC lacks a framework to identify and validate the decision rule applied to predicted class scores [Ferrer 2022].
- MCC depends on the definition of TN (undefined for ObD and InS).

Fig. SN 3.46. Metric profile of Matthews Correlation Coefficient (MCC). The upward arrow in the value range indicates that higher values are better than lower values. Abbreviations: Expected Cost (EC), False Negative (FN), False Positive (FP), Instance Segmentation (InS), Object Detection (ObD), True Negative (TN), True Positive (TP). References: Ferrer, 2022: [24], Matthews, 1975: [46], Reinke et al., 2021: [54], Zhu, 2020: [72]. Mentioned figures: Figs. 4b, SN 2.7, SN 2.9, SN 2.35.

NET BENEFIT (NB)

$$NB = \frac{TP}{TP + TN + FP + FN} - \frac{FP}{TP + TN + FP + FN} \cdot \left(\frac{p_t}{1 - p_t}\right)$$

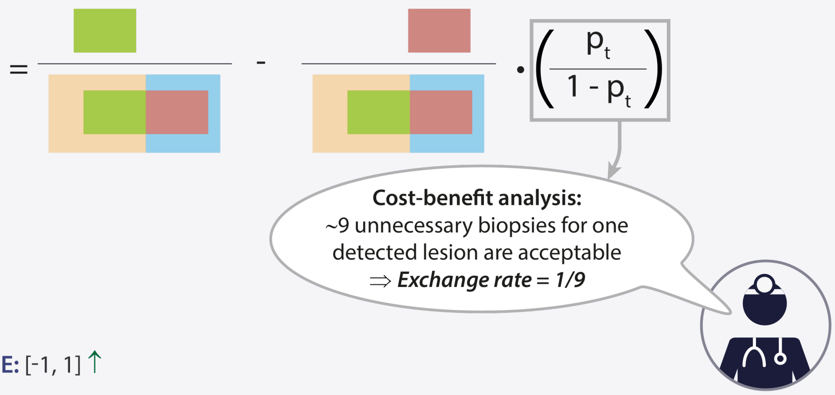

VALUE RANGE: [-1, 1] ↑

DESCRIPTION

NB validates the quality of a model intended to support a specific clinical decision. NB gives the 'net' proportion of TPs that results from a prediction. This is equivalent to the proportion of TPs in the absence of FPs. For its calculation, NB considers a task-related risk threshold (= exchange rate between the benefit of TPs and harm of FPs).

When varying the risk threshold over a 'reasonable range' of possible thresholds, plotting NB by risk threshold yields a decision curve. It is a strictly proper performance measure.

DEFINITION

[Vickers and Elkin, 2006]

CARDINALITIES

TP	FP	FN	TN
●	●	●	●

METRIC FAMILY

Counting metric	Multi-threshold metric	Distance-based metric
●	○	○

PREVALENCE DEPENDENCY? ●

RELEVANT PITFALLS

- NB requires the availability of predicted class scores. These should reflect the true probabilities (calibrated scores) (Fig. SN 2.20).
- Decision curves analysis (i.e., NB plotted over a range of decision thresholds) can only be applied if relevant decision thresholds can be defined [Vickers et al., 2016].
- Compared to other metrics like EC, NB lacks a framework to identify and validate the decision rule applied to predicted class scores [Ferrer 2022].
- NB is popular in clinical studies but rather uncommon in image analysis, thus potentially preventing an easy comparison with other publications.

Fig. SN 3.47. Metric profile of Net Benefit (NB). The upward arrow in the value range indicates that higher values are better than lower values. Abbreviations: Expected Cost (EC), False Negative (FN), False Positive (FP), True Negative (TN), True Positive (TP). References: Ferrer, 2022: [24], Vickers and Elkin, 2006: [64], Vickers et al., 2016: [65]. Mentioned figure: Fig. SN 2.20.

NEGATIVE PREDICTIVE VALUE (NPV)

$$\mathrm{NPV} = \frac{\mathrm{TN}}{\mathrm{TN} + \mathrm{FN}} =$$

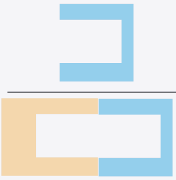

if Prevalence = 0.5

$$\mathrm{NPV}_{\mathrm{corrected}} = \frac{\text{Specificity} \cdot (1 - \text{Prevalence})}{\text{Specificity} \cdot (1 - \text{Prevalence}) + (1 - \text{Sensitivity}) \cdot \text{Prevalence}}$$

VALUE RANGE: [0, 1] ↑

DESCRIPTION

NPV represents the probability of a negative prediction corresponding to an actual negative sample.

DEFINITION

[Tharwat, 2020]

PREVALENCE DEPENDENCY

● (yes)

CARDINALITIES

TP	FP	FN	TN
○	○	●	●

METRIC FAMILY

Counting metric	Multi-threshold metric	Distance-based metric
●	○	○

RELEVANT PITFALLS

- NPV is prevalence-dependent, thus not comparable across data sets with different prevalences if not corrected (Figs. SN 2.7, SN 2.15, [Reinke et al., 2021]).
- The expected value for an uninformed classifier depends on class imbalance. The lower the prevalence, the higher the expected value (Figs. SN 2.7, SN 2.15).
- NPV does not allow for incorporating unequal severity of confusions across classes. This implies that the metric is not applicable to cost-benefit analyses (Fig. SN 2.9), or when target classes are related on an ordinal scale (Fig. 4b).
- NPV depends on the definition of TN (undefined for ObD and InS).
- NPV is undefined if the number of TN and FN is zero, and ad hoc rules need to be established depending on the use case (Fig. SN 2.18, Extended Data Fig. 2b).
- NPV value depends on the definition of the positive class [Reinke et al., 2021].

Fig. SN 3.48. Metric profile of Negative Predictive Value (NPV). The upward arrow in the value range indicates that higher values are better than lower values. Abbreviations: False Negative (FN), False Positive (FP), Instance Segmentation (InS), Object Detection (ObD), True Negative (TN), True Positive (TP). References: Reinke et al., 2021: [54], Tharwat, 2020: [60]. Mentioned figures: Figs. 4b, SN 2.7, SN 2.9, SN 2.15, SN 2.18, Extended Data Fig. 2b.

PANOPTIC QUALITY (PQ)

VALUE RANGE: [0, 1] ↑

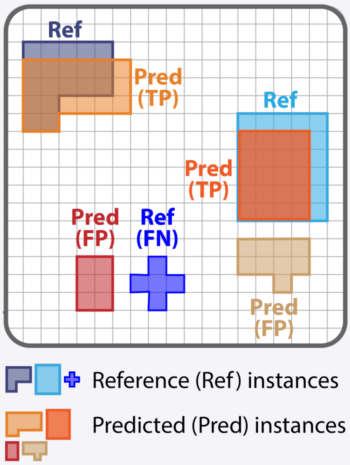

$$PQ = \frac{\sum_{(Ref,\,Pred)\in TP} IoU(Ref, Pred)}{|TP| + 0.5\,|FP| + 0.5\,|FN|}$$

$$= \underbrace{\frac{\sum_{(Ref,\,Pred)\in TP} IoU(Ref, Pred)}{|TP|}}_{\text{Segmentation quality}} \cdot \underbrace{\frac{|TP|}{|TP| + 0.5\,|FP| + 0.5\,|FN|}}_{\text{Detection quality}}$$

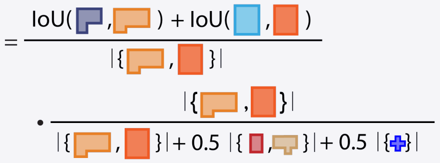

DESCRIPTION

PQ assesses segmentation and detection quality in one metric. The segmentation quality is measured by averaging the IoU scores of all TP instances. The detection quality is measured by the F_1 Score. While in the F_1 Score, each TP counts as "1", PQ replaces this "1" score in the numerator with the IoU score of each TP.

The F_1 Score as a detection metric implies two cutoffs:

1. a prior cutoff on a localization criterion for matching and
2. a prior cutoff on object class scores to generate a confusion matrix.

In this context, PQ can be interpreted as making the localization quality in F_1 Score explicit (1) and thus only relying on the cutoff on class scores (2).

DEFINITION

[Kirillov et al., 2019]

CARDINALITIES

TP	FP	FN	TN
○	○	●	●

METRIC FAMILY

Counting metric	Multi-threshold metric	Distance-based metric
●	○	○

RELEVANT PITFALLS

Only inspecting the PQ value does not show the tradeoff between segmentation and detection quality. One of the terms may overrule the other without it being noticed [Reinke et al., 2021].

Fig. SN 3.49. Metric profile of Panoptic Quality (PQ). The upward arrow in the value range indicates that higher values are better than lower values. Abbreviations: Average Precision (AP), False Negative (FN), False Positive (FP), Free-Response Receiver Operating Characteristic (FROC), Intersection over Union (IoU), True Negative (TN), True Positive (TP). References: Kirillov et al., 2019: [36], Reinke et al., 2021: [54].

POSITIVE LIKELIHOOD RATIO (LR+)

Synonyms: Likelihood ratio positive, Likelihood ratio for positive results

$$LR+ = \frac{\text{Sensitivity}}{1 - \text{Specificity}} =$$

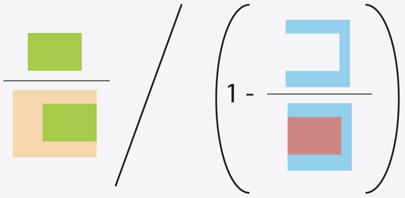

VALUE RANGE: [0, ∞) ↑

DESCRIPTION

LR+ indicates the factor by which a positive prediction occurs more frequently among actual positive samples than among actual negative samples. In a clinical example where the quality of a diagnostic test is to be assessed, this could be interpreted as how much more likely a positive test result is for a diseased person compared to a healthy person (the higher the better).

DEFINITION

[Attia, 2003]

PREVALENCE DEPENDENCY ○

METRIC FAMILY

Counting metric	Multi-threshold metric	Distance-based metric
●	○	○

CARDINALITIES

TP	FP	FN	TN
●	●	●	●

RELEVANT PITFALLS

- LR+ is insensitive to changes in predictive values (PPV and NPV; Fig. 5a).
- LR+ is undefined if the Specificity is 1, and ad hoc rules need to be established depending on the use case (Fig. SN 2.18, Extended Data Fig. 2b).
- LR+ does not allow for incorporating unequal severity of confusions across classes. This implies that the metric is not applicable to cost-benefit analyses (Fig. SN 2.9), or when target classes are related on an ordinal scale (Fig. 4b).
- LR+ depends on the definition of the positive class [Reinke et al., 2021].
- LR+ may yield the same value for different Sensitivity and Specificity scores [Reinke et al., 2021].
- LR+ depends on the definition of TN (undefined for ObD and InS).

Fig. SN 3.50. Metric profile of Positive Likelihood Ratio (LR+). The upward arrow in the value range indicates that higher values are better than lower values. Abbreviations: False Negative (FN), False Positive (FP), Instance Segmentation (InS), Object Detection (ObD), Positive Predictive Value (PPV), True Negative (TN), True Positive (TP). References: Attia, 2003: [2], Reinke et al., 2021: [54]. Mentioned figures: Figs. 4b, 5a, SN 2.9, SN 2.18, Extended Data Fig. 2b.

POSITIVE PREDICTIVE VALUE (PPV)

Synonym: Precision

$$PPV = \frac{TP}{TP + FP} =$$

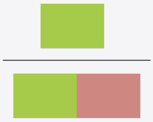

if Prevalence = 0.5

$$PPV_{corrected} = \frac{\text{Sensitivity} \bullet \text{Prevalence}}{\text{Sensitivity} \bullet \text{Prevalence} + (1 - \text{Specificity}) \bullet (1 - \text{Prevalence})}$$

VALUE RANGE: [0, 1] ↑

DESCRIPTION

PPV represents the probability of a positive prediction corresponding to an actual positive sample.

DEFINITION

[Tharwat, 2020]

CARDINALITIES

TP	FP	FN	TN
●	●	○	○

PREVALENCE DEPENDENCY ●

METRIC FAMILY

Counting metric	Multi-threshold metric	Distance-based metric
●	○	○

RELEVANT PITFALLS

- PPV is prevalence-dependent, thus not comparable across data sets with different prevalences if not corrected (Figs. SN 2.7, SN 2.15, [Reinke et al., 2021]).
- The expected value for an uninformed classifier depends on class imbalance (Figs. SN 2.7, SN 2.15). The higher the prevalence, the higher the expected value.
- PPV does not allow for incorporating unequal severity of confusions across classes. This implies that the metric is not applicable to cost-benefit analyses (Fig. SN 2.9), or when target classes are related on an ordinal scale (Fig. 4b).
- PPV is undefined if the prediction is empty, and ad hoc rules need to be established depending on the use case (Fig. SN 2.18, Extended Data Fig. 2b).
- PPV depends on the definition of the positive class [Reinke et al., 2021].
- PPV does not penalize FN predictions [Reinke et al., 2021].

Fig. SN 3.51. Metric profile of the Positive Predictive Value (PPV). The upward arrow in the value range indicates that higher values are better than lower values. Abbreviations used in the figure: False Negative (FN), False Positive (FP), Instance Segmentation (InS), Object Detection (ObD), True Negative (TN), True Positive (TP). References used in the figure: Reinke et al., 2021: [54], Tharwat, 2020: [60]. Mentioned figures: Figs. 4b, SN 2.7, SN 2.9, SN 2.15, SN 2.18, Extended Data Fig. 2b.

SENSITIVITY

Synonyms: Recall, True Positive Rate (TPR), Hit Rate

$$\text{Sensitivity} = \frac{TP}{TP + FN} =$$

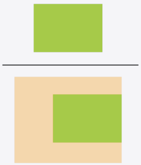

VALUE RANGE: [0, 1] ↑

DESCRIPTION

Sensitivity measures how good a method is in classifying truly positive samples as positive.

DEFINITION

[Tharwat, 2020]

CARDINALITIES

TP	FP	FN	TN
●	○	●	○

PREVALENCE DEPENDENCY ○

METRIC FAMILY

Counting metric	Multi-threshold metric	Distance-based metric
●	○	○

RELEVANT PITFALLS

- Sensitivity is insensitive to changes in predictive values (PPV and NPV).
- Sensitivity does not penalize FP predictions [Reinke et al., 2021]. For example, a trivial classifier always predicting the positive class yields a Sensitivity of 1 but a Specificity of 0.
- Sensitivity does not allow for incorporating unequal severity of confusions across classes. This implies that the metric is not applicable to cost-benefit analyses (Fig. SN 2.9), or when target classes are related on an ordinal scale (Fig. 4b).
- Applying Sensitivity for optimization-based decision rules is challenging [Maier-Hein et al., 2022].
- Sensitivity at image level is undefined in segmentation and ObD tasks if the reference is empty, and ad hoc rules need to be established depending on the use case (Fig. SN 2.18, Extended Data Fig. 2b).
- Sensitivity depends on the definition of the positive class [Reinke et al., 2021].

Fig. SN 3.52. Metric profile of Sensitivity. The upward arrow in the value range indicates that higher values are better than lower values. Abbreviations: False Negative (FN), False Positive (FP), Object Detection (ObD), Positive Predictive Value (PPV), True Negative (TN), True Positive (TP). References: Maier-Hein et al., 2022: [44], Reinke et al., 2021: [54], Tharwat, 2020: [60]. Mentioned figures: Figs. 4b, SN 2.9, SN 2.18, Extended Data Fig. 2b.

SPECIFICITY

Synonyms: Selectivity, True Negative Rate (TNR)

$$\text{Specificity} = \frac{TN}{TN + FP} =$$

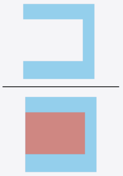

VALUE RANGE: [0, 1] ↑

DESCRIPTION
Specificity measures how good a method is in classifying truly negative samples as negative.

DEFINITION
[Tharwat, 2020]

CARDINALITIES

TP	FP	FN	TN
○	●	○	●

PREVALENCE DEPENDENCY ○

METRIC FAMILY

Counting metric	Multi-threshold metric	Distance-based metric
●	○	○

RELEVANT PITFALLS
- Specificity is insensitive to changes in predictive values (PPV and NPV).
- Specificity does not penalize FN predictions [Reinke et al., 2021]. A trivial classifier always predicting the negative class, for example, yields a Specificity of 1 but a Sensitivity of 0.
- Specificity does not allow for incorporating unequal severity of confusions across classes. This implies that the metric is not applicable to cost-benefit analyses (Fig. SN 2.9), or when target classes are related on an ordinal scale (Fig. 4b).
- Specificity at image level is undefined in segmentation and ObD tasks if both TN and FP are zero, and ad hoc rules need to be established depending on the use case (Fig. SN 2.18, Extended Data Fig. 2b).
- Specificity depends on the definition of TN (undefined for ObD and InS).
- Specificity depends on the definition of the positive class [Reinke et al., 2021].

Fig. SN 3.53. Metric profile of Specificity. The upward arrow in the value range indicates that higher values are better than lower values. Abbreviations: False Negative (FN), False Positive (FP), True Negative (TN), True Positive (TP). References: Reinke et al., 2021: [54], Tharwat, 2020: [60]. Mentioned figures: Figs. 4b SN 2.9, SN 2.18, Extended Data Fig. 2b.

WEIGHTED COHEN'S KAPPA (WCK)

Synonyms: Weighted Cohen's Kappa Coefficient, Weighted Kappa Statistic, Weighted Kappa Score

$$\mathrm{WCK} = \frac{p_0^w - p_e^w}{1 - p_e^w}, \quad p_0^w = \frac{w_{TP}\mathrm{TP} + w_{TN}\mathrm{TN} + w_{FP}\mathrm{FP} + w_{FN}\mathrm{FN}}{\mathrm{TP}+\mathrm{TN}+\mathrm{FP}+\mathrm{FN}} =$$

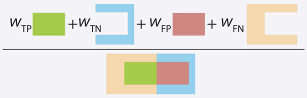

$$p_e^w = w_{TP}\frac{(\mathrm{TP}+\mathrm{FP})(\mathrm{TP}+\mathrm{FN})}{\mathrm{TP}+\mathrm{TN}+\mathrm{FP}+\mathrm{FN}} + w_{TN}\frac{(\mathrm{TN}+\mathrm{FP})(\mathrm{TN}+\mathrm{FN})}{\mathrm{TP}+\mathrm{TN}+\mathrm{FP}+\mathrm{FN}} + w_{FN}\frac{(\mathrm{FN}+\mathrm{FP})(\mathrm{FN}+\mathrm{TN})}{\mathrm{TP}+\mathrm{TN}+\mathrm{FP}+\mathrm{FN}} + w_{FP}\frac{(\mathrm{FP}+\mathrm{TP})(\mathrm{FP}+\mathrm{TN})}{\mathrm{TP}+\mathrm{TN}+\mathrm{FP}+\mathrm{FN}}$$

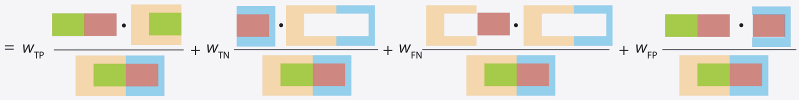

VALUE RANGE: [-1, 1] ↑

A value of 0 refers to a prediction which is not better than random guessing.

$w_{TP}/w_{TN}/w_{FP}/w_{FN}$: (estimation of) costs of the respective cardinalities; can be adjusted as a weighting of them.

DESCRIPTION

WCK calculates the degree of agreement between the reference and prediction while incorporating the agreement resulting from chance. WCK is a generalization of CK with 0-1 weights.

DEFINITION

[Cohen, 1960]

CARDINALITIES

TP	FP	FN	TN
●	●	●	●

PREVALENCE DEPENDENCY ●

METRIC FAMILY

Counting metric	Multi-threshold metric	Distance-based metric
●	○	○

MULTI-CLASS DEFINITION

For C classes, WCK can be defined as: $\mathrm{WCK} = 1 - \left(\sum_{i=1}^{C}\sum_{j=1}^{C} w_{ij} \cdot n_{ij}\right) \Big/ \left(\sum_{i=1}^{C}\sum_{j=1}^{C} w_{ij} \cdot \frac{n_{i.} \cdot n_{.j}}{N^2}\right)$

n_{ij}: entry of the confusion matrix for row i and column j, i.e. samples of actual class i that were predicted as class j

N: total number of samples

$n_{i.}$: sum of entries of row i of the confusion matrix

$n_{.j}$: sum of entries of column j of the confusion matrix

w_{ij}: costs for the entry of the confusion matrix for row i and column j, i.e., the cost for samples of actual class i that were predicted as class j

RELEVANT PITFALLS

- WCK is prevalence-dependent, thus not comparable across data sets and may yield different rankings than the BA (Figs. SN 2.7, SN 2.15, [Reinke et al., 2021]).
- The theoretical lower bound of WCK (-1) may not always be achievable (Fig. SN 2.35).
- In settings where target classes lie on an ordinal scale, WCK may harshly penalize label shifts [Reinke et al., 2021].
- WCK is hard to interpret [Delgado and Tibau, 2019].
- WCK was designed for symmetric situations (guesses of two raters) [Powers, 2012].
- Compared to other multi-class metrics like EC, WCK lacks a framework to identify and validate the decision rule applied to predicted class scores [Ferrer, 2022].
- WCK with quadratic weights may yield "paradoxical results" [Warrens, 2012].
- The selection of weights to different types of mistakes is problem-dependent and requires domain knowledge.
- WCK depends on the definition of TN (undefined for ObD and InS).

Fig. SN 3.54. Metric profile of Weighted Cohen's Kappa (WCK). The upward arrow in the value range indicates that higher values are better than lower values. Abbreviations: Balanced Accuracy (BA), Cohen's Kappa (CK), Expected Cost (EC), False Negative (FN), False Positive (FP), Instance Segmentation (InS), Object Detection (ObD), True Negative (TN), True Positive (TP). References: Cohen, 1960: [13], Delgado and Tibau, 2019: [18], Ferrer, 2022: [24], Powers, 2012: [53], Reinke et al., 2021: [54], Warrens, 2012: [67]. Mentioned figures: Figs. SN 2.7, SN 2.15, SN 2.35.

3.1.2 Multi-threshold metrics.

AREA UNDER THE RECEIVER OPERATING CHARACTERISTIC CURVE (AUROC)

Synonyms: AUC (Area under Curve), AUC - ROC (Area Under The Curve - Receiver Operating Characteristics), C-Index, C-Statistics

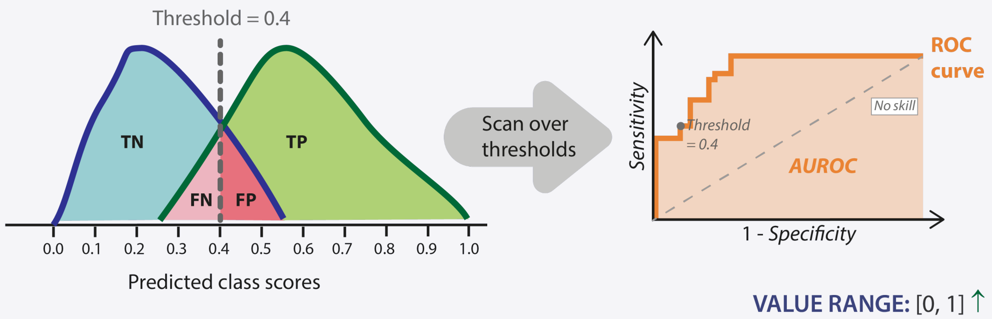

VALUE RANGE: [0, 1] ↑

An AUROC value of 0.5 refers to a prediction which is not better than random guessing.

DESCRIPTION

AUROC measures the area under the ROC curve and indicates how well the probabilities of the positive class are separated from those of the negative class. In other words, AUROC represents the probability of a randomly sampled positive case having a higher predicted class score than a randomly sampled negative case.

DEFINITION

[Hanley and McNeil, 1982]

CARDINALITIES

TP	FP	FN	TN
●	●	●	●

METRIC FAMILY

Counting metric	Multi-threshold metric	Distance-based metric
○	●	○

RELEVANT PITFALLS

- AUROC is not designed for object-level validation, i.e., does not measure localization performance, and is invariant to the number of reference and predicted objects (Fig. SN 2.1).
- AUROC tends to be overly optimistic for imbalanced datasets (Fig. 4a).
- Small sample sizes yield large confidence intervals for AUROC (Fig. SN 2.16).
- In the absence of predicted class scores, AUROC should not be calculated, otherwise, ad hoc rules need to be defined (Fig. SN 2.20).
- Small changes in the predicted class scores may heavily affect the AUROC score [Reinke et al., 2021].
- Within the ranking, predicted class scores are neglected, not affecting the AUROC score.
- AUROC depends on the definition of TN (undefined for ObD and InS).

Fig. SN 3.55. Metric profile of Area under the Receiver Operating Characteristic Curve (AUROC). The upward arrow in the value range indicates that higher values are better than lower values. Abbreviations: False Negative (FN), False Positive (FP), Instance Segmentation (InS), Object Detection (ObD), Receiver Operating Characteristic (ROC), True Negative (TN), True Positive (TP). References: Hanley and McNeil, 1982: [31], Reinke et al., 2021: [54]. Mentioned figures: Figs. 5a, SN 2.1, SN 2.16, SN 2.20.

AVERAGE PRECISION (AP)

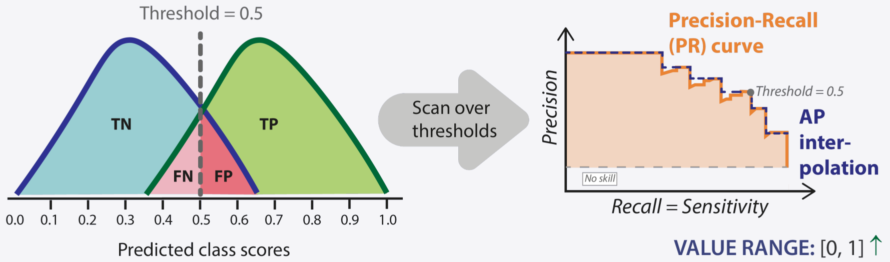

VALUE RANGE: [0, 1] ↑

DESCRIPTION

AP measures the interpolated area under the PR curve. It differs from the well known ROC curve and the associated AUROC metric by measuring Sensitivity in combination with PPV instead of in combination with Specificity. This replacement has the effect that TNs are not explicitly considered by the PR curve and AP. Ignoring TN can be desirable in settings with a dominating negative class and thus large amounts of TN suppressing a focus on the rare positive class. A prominent example are tasks with imbalanced classes such as those with retrieval character, where AUROC is typically not applied, because the large amount of TNs leads to an insensitivity to subtle performance changes for the rare positive class.

CARDINALITIES

TP	FP	FN	TN
●	●	●	○

METRIC FAMILY

Counting metric	Multi-threshold metric	Distance-based metric
○	●	○

DEFINITION

[Lin et al., 2014; Everingham et al. 2015]

RELEVANT PITFALLS

- The implementation of AP is not standardized across common libraries (Fig. 6a).
- Unlike AUROC, AP has no fixed random reference value (0.5), but the random reference depends on the prevalence (Fig. SN 2.7).
- In the absence of predicted class scores, AP should not be calculated, otherwise, ad hoc rules need to be defined (Fig. SN 2.20).
- Within the ranking, predicted class scores are neglected, not affecting the AP score [Reinke et al., 2021].
- AP is computed over the full data set, thus not sensitive to performance on single images [Reinke et al., 2021].
- Compared to AUROC, AP's interpretability is limited [Maier-Hein et al., 2022].
- For ObD and InS problems, FP predictions with low class scores do not affect the AP score [Reinke et al., 2021]. For filtering low confidence predictions from the AP calculation, a cutoff on confidence scores is required [Maier-Hein et al., 2022].

Fig. SN 3.56. Metric profile of Average Precision (AP). The upward arrow in the value range indicates that higher values are better than lower values. Abbreviations: Area under the Receiver Operating Characteristic Curve (AUROC), False Negative (FN), False Positive (FP), Instance Segmentation (InS), Object Detection (ObD), Precision-Recall (PR), True Negative (TN), True Positive (TP). References: Everingham et al., 2015: [22], Lin et al., 2014: [42], Maier-Hein et al., 2022: [44], Reinke et al., 2021: [54]. Mentioned figures: Figs. 6a, SN 2.7, SN 2.20.

FREE-RESPONSE RECEIVER OPERATING CHARACTERISTIC (FROC) SCORE

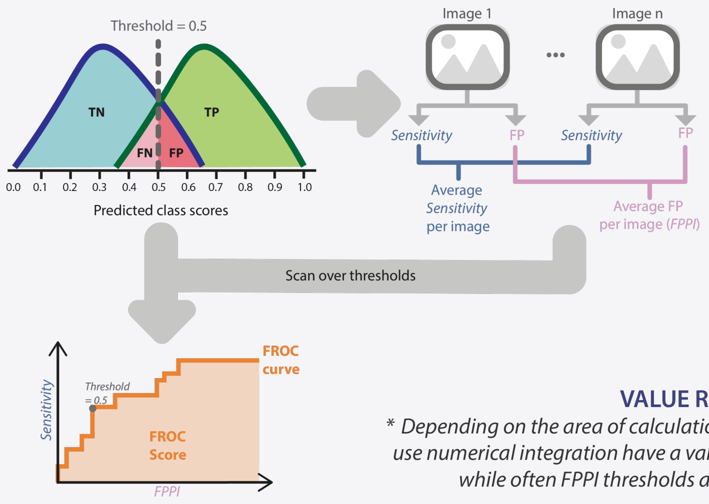

VALUE RANGE: [0, ∞*) ↑

** Depending on the area of calculation. Versions which use numerical integration have a value range of [0, ∞), while often FPPI thresholds are limited to [0, 1].*

DESCRIPTION

FROC Score approximates the area under the FROC curve, which plots the Sensitivity as a function of the average number of FPPI. It thus indicates how well the probabilities of the positive class are separated from those of the negative class while considering object-level information.

DEFINITION

[Van Ginneken et al., 2010]

METRIC FAMILY

Counting metric	Multi-threshold metric	Distance-based metric
○	●	○

CARDINALITIES

TP	FP	FN	TN
●	●	●	○

RELEVANT PITFALLS

- In the absence of predicted class scores, the FROC score should not be calculated, otherwise, ad hoc rules need to be defined (Fig. SN 2.20).
- FROC is not standardized, i.e., the FPPI values used to calculate the score differ across studies and sometimes numerical integration is used instead of discrete FPPI values (Fig. SN 2.21).
- If numerical integration is employed, the FROC Score is not bounded between 0 and 1 (Fig. SN 2.21).

Fig. SN 3.57. Metric profile of Free-Response Receiver Operating Characteristic (FROC). The upward arrow in the value range indicates that higher values are better than lower values. Abbreviations: False Negative (FN), False Positive (FP), False Positives per Image (FPPI), True Negative (TN), True Positive (TP). References: Van Ginneken et al., 2010: [62]. Mentioned figures: Figs. SN 2.20, SN 2.21.

3.1.3 *Distance-based metrics.*

AVERAGE SYMMETRIC SURFACE DISTANCE (ASSD)

Synonym: Weighted bilateral mean contour distance

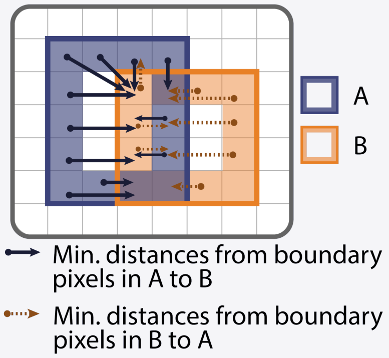

$$d(a,B) = \min_{b \in B} d(a,b)$$

$$ASSD(A,B) = \frac{\sum_{a \in A} d(a,B) + \sum_{b \in B} d(b,A)}{|A| + |B|}$$

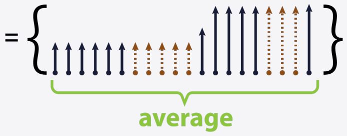

VALUE RANGE: [0, ∞) ↓

DESCRIPTION

ASSD measures the average of all shortest boundary distances between contour A to any point on contour B and vice versa, symmetrically.

DEFINITION

[Yeghiazaryan, Varduhi and Voiculescu, 2015]

METRIC FAMILY

Counting metric ○ | Multi-threshold metric ○ | Distance-based metric ●

RELEVANT PITFALLS

- ASSD does not compensate for inter-rater variability (Figs. 5c, SN 2.17).
- Depending on the implementation, ASSD may ignore holes in the segmentation (Fig. SN 2.4).
- ASSD ignores the overlap and volume of structures (Fig. SN 2.4).
- ASSD is undefined if either the reference or the prediction is empty, and ad hoc rules need to be established depending on the use case (Fig. SN 2.18).
- ASSD weights large object boundaries more than small object boundaries [Yeghiazaryan, Varduhi and Voiculescu, 2015].
- ASSD is unbounded to the top, impeding missing value handling [Reinke et al., 2021]. Ad hoc rules have been defined in the past based on the use case, e.g., set the largest penalty equal to the largest distance within an image.
- ASSD scores may differ for different boundary extraction methods.

Fig. SN 3.58. Metric profile of Average Symmetric Surface Distance (ASSD). The downward arrow in the value range indicates that lower values are better than higher values. Abbreviation: Semantic Segmentation (SemS). References: Reinke et al., 2021: [54], Yeghiazaryan, Varduhi and Voiculescu, 2015: [70]. Mentioned figures: Figs. 5c, SN 2.4, SN 2.17, SN 2.18.

BOUNDARY INTERSECTION OVER UNION (BOUNDARY IOU)

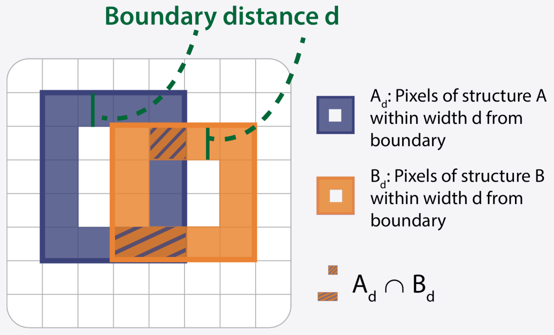

Boundary IoU(A,B) =

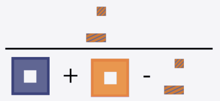

$$= \frac{|A_d \cap B_d|}{|A_d| + |B_d| - |A_d \cap B_d|}$$

$$= \frac{|A_d \cap B_d|}{|A_d \cup B_d|}$$

VALUE RANGE: [0, 1] ↑

DESCRIPTION

Boundary IoU measures the overlap between the predicted and reference boundaries up to a predefined width d.

DEFINITION

[Cheng et al., 2021]

METRIC FAMILY

Counting metric	Multi-threshold metric	Distance-based metric
○	○	●

RELEVANT PITFALLS

- Depending on the choice of the hyperparameter d, Boundary IoU may ignore the overlap and volume of structures (Fig. SN 2.4).
- Boundary IoU is undefined if the reference and prediction are empty, and ad hoc rules need to be established depending on the use case (Fig. SN 2.18).
- Boundary IoU may yield a perfect value for only predicting the boundary pixels, missing the pixels inside the object [Reinke et al., 2021].
- Boundary IoU includes the hyperparameter d, which needs to be chosen wisely (user or use case-defined threshold) [Reinke et al., 2021]. Depending on the choice of the parameter, Boundary IoU does not compensate for inter-rater variability (Figs. 5c, SN 2.17).

Fig. SN 3.59. Metric profile of the Boundary Intersection over Union (IoU). The upward arrow in the value range indicates that higher values are better than lower values. References: Cheng et al., 2021: [10], Reinke et al., 2021: [54]. Mentioned figures: Figs. 5c, SN 2.4, SN 2.17, SN 2.18.

HAUSDORFF DISTANCE (HD)

Synonyms: Maximum Symmetric Surface Distance, Hausdorff Metric, Pompeiu–Hausdorff Distance

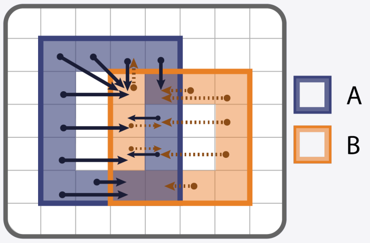

→ Min. distances from boundary pixels in A to B

⇢ Min. distances from boundary pixels in B to A

$$d(a,B) = \min_{b \in B} d(a,b)$$

$$HD(A,B) = \max \left\{ \max_{a \in A} d(a,B), \max_{b \in B} d(b,A) \right\}$$

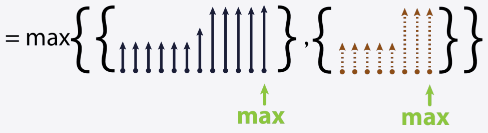

VALUE RANGE: [0, ∞) ↓

DESCRIPTION

HD is the largest of all the distances from a point on one boundary to the closest point on the other boundary.

DEFINITION

[Huttenlocher, 1993]

METRIC FAMILY

Counting metric	Multi-threshold metric	Distance-based metric
○	○	●

RELEVANT PITFALLS

- Single outliers may substantially influence the HD score (Fig. 7c).
- HD ignores holes in the segmentation (Fig. 13).
- HD ignores the overlap and volume of structures (Fig. 13).
- HD does not compensate for inter-rater variability (Fig. 26).
- HD is undefined if either the reference or the prediction is empty, and ad hoc rules need to be defined depending on the use case (Fig. 27).
- HD is unbounded to the top, impeding missing value handling [Reinke et al., 2021]. Ad hoc rules have been defined in the past based on the use case, e.g., set the largest penalty equal to the largest distance within an image.
- HD scores may differ for different boundary extraction methods.

Fig. SN 3.60. Metric profile of Hausdorff Distance (HD). The downward arrow in the value range indicates that lower values are better than higher values. Abbreviation: Semantic Segmentation (SemS). References : Huttenlocher, 1993: [34], Reinke et al., 2021: [54]. Mentioned figures: Figs. 5c, SN 2.4, SN 2.17, SN 2.18.

MEAN AVERAGE SURFACE DISTANCE (MASD)

Synonym: Mean Surface Distance

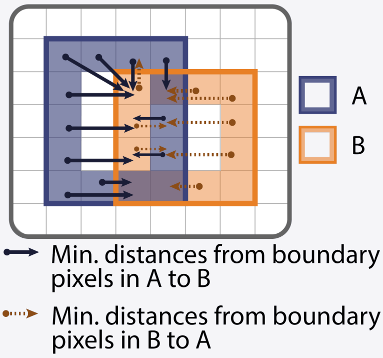

$$d(\mathrm{a},\mathrm{B}) = \min_{\mathrm{b}\in\mathrm{B}} d(\mathrm{a},\mathrm{b})$$

$$\mathrm{MASD}(\mathrm{A},\mathrm{B}) = \frac{1}{2}\left(\frac{\sum_{\mathrm{a}\in\mathrm{A}} d(\mathrm{a},\mathrm{B})}{|\mathrm{A}|} + \frac{\sum_{\mathrm{b}\in\mathrm{B}} d(\mathrm{b},\mathrm{A})}{|\mathrm{B}|}\right)$$

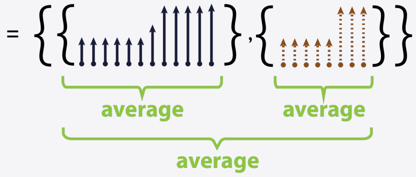

VALUE RANGE: [0, ∞) ↓

DESCRIPTION

MASD measures the mean of the averages over all shortest distances from all sampled points on one boundary to any other point on another boundary.

DEFINITION

[Beneš and Zitová, 2015]

METRIC FAMILY

Counting metric ○ | Multi-threshold metric ○ | Distance-based metric ●

RELEVANT PITFALLS

- MASD does not compensate for inter-rater variability (Figs. 5c, SN 2.17).
- Depending on the implementation, MASD may ignore holes in the segmentation (Fig. SN 2.4).
- MASD ignores the overlap and volume of structures (Fig. SN 2.4).
- MASD only depends on one boundary if the prediction is very small and close to the reference boundary [Reinke et al., 2021].
- MASD is undefined if either the reference or the prediction is empty, and ad hoc rules need to be established depending on the use case (Fig. SN 2.18).
- MASD is unbounded to the top, impeding missing value handling [Reinke et al., 2021]. Ad hoc rules have been defined in the past based on the use case, e.g., set the largest penalty equal to the largest distance within an image.
- MASD scores may differ for different boundary extraction methods.

Fig. SN 3.61. Metric profile of Mean Average Surface Distance (MASD). The downward arrow in the value range indicates that lower values are better than higher values. Abbreviation: Semantic Segmentation (SemS). References: Beneš and Zitová, 2015: [6], Reinke et al., 2021: [54]. Mentioned figures: Figs. 5c, SN 2.4, SN 2.17, SN 2.18.

NORMALIZED SURFACE DISTANCE (NSD)

Synonyms: Normalized Surface Dice, Surface Distance, Surface Dice, Surface DSC

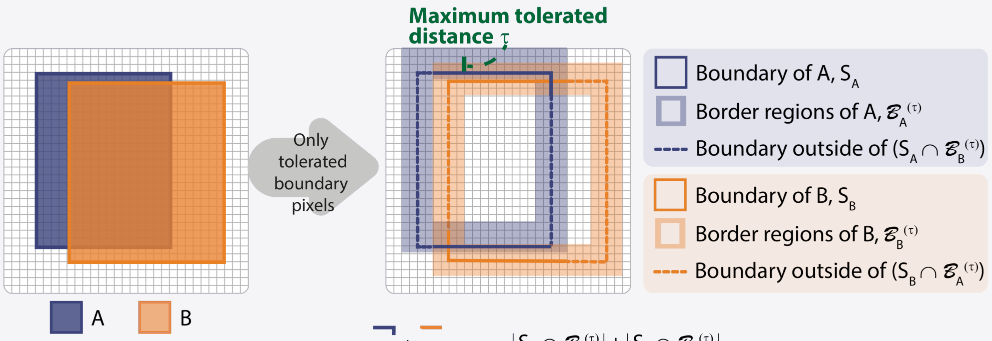

$$NSD(A,B)^{(\tau)} = \frac{|S_A \cap \mathcal{B}_B^{(\tau)}| + |S_B \cap \mathcal{B}_A^{(\tau)}|}{|S_A| + |S_B|}$$

VALUE RANGE: [0, 1] ↑

DESCRIPTION

NSD measures the DSC on boundary pixels with an uncertainty margin. The degree of strictness for what constitutes a correct boundary is represented by the tolerance parameter τ. Only boundary parts within the border regions defined by τ are counted as TP. NSD therefore captures known uncertainties in the reference and allows acceptable deviations from the reference for the predicted boundary.

DEFINITION

[Nikolov et al., 2021]

METRIC FAMILY

- Counting metric (●)
- Multi-threshold metric (○)
- Distance-based metric (○)

RELEVANT PITFALLS

- NSD ignores the general overlap and volume of structures (Fig. SN 2.4).
- NSD is undefined if the reference and prediction are empty, and ad hoc rules need to be established depending on the use case (Fig. SN 2.18).
- NSD may yield a perfect value for only predicting the boundary pixels, missing the pixels inside the object [Reinke et al., 2021].
- NSD includes the hyperparameter τ, which needs to be chosen wisely (user or use case-defined threshold) [Reinke et al., 2021].

Fig. SN 3.62. Metric profile of Normalized Surface Distance (NSD). The upward arrow in the value range indicates that higher values are better than lower values. Abbreviation: Dice Similarity Coefficient (DSC). References: Nikolov et al., 2021: [50], Reinke et al., 2021: [54]. Mentioned figures: Figs. SN 2.4, SN 2.18.

X^{TH} PERCENTILE OF HAUSDORFF DISTANCE

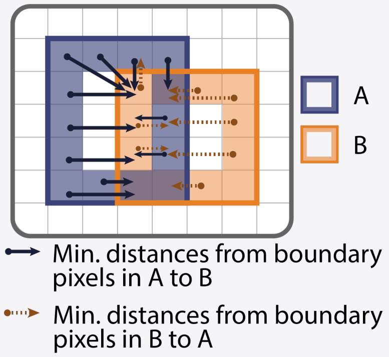

EXAMPLE: 95th percentile

$$d_{95}(A,B) = X_{95\, a\in A} \left\{ \min_{b\in B} d(a,b) \right\}$$

$$HD95(A,B) = \max \left\{ d_{95}(A,B), d_{95}(B,A) \right\}$$

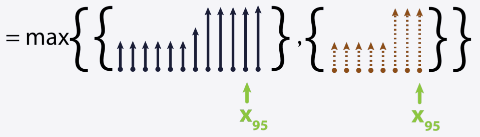

VALUE RANGE: $[0, \infty)$ ↓

DESCRIPTION

The xth percentile of the Hausdorff Distance (HD) measures the xth percentile of all the distances from a point on one boundary to the closest point on the other boundary. A common value is x = 95 (HD95).

DEFINITION

[Huttenlocher, 1993]

METRIC FAMILY

Counting metric	Multi-threshold metric	Distance-based metric
○	○	●

RELEVANT PITFALLS

- Depending on the implementation, the xth Percentile of HD ignores holes in the segmentation (Fig. SN 2.4).
- The xth percentile of HD ignores the overlap and volume of structures (Fig. SN 2.4).
- The xth Percentile of HD does not compensate for inter-rater variability (Fig. SN 2.17).
- The xth Percentile of HD is undefined if either the reference or the prediction is empty, and ad hoc rules need to be defined depending on the use case (Fig. SN 2.18).
- The xth Percentile of HD is unbounded to the top [Reinke et al., 2021], impeding missing value handling. Ad hoc rules have been defined in the past based on the use case, e.g., set the largest penalty equal to the largest distance within an image.
- The scores of the xth percentile of the HD may differ for different boundary extraction methods.

Fig. SN 3.63. Metric profile of Xth Percentile of Hausdorff Distance (HD). The downward arrow in the value range indicates that lower values are better than higher values. Abbreviations: Hausdorff Distance (HD), Semantic Segmentation (SemS). References: Huttenlocher, 1993: [34], Reinke et al., 2021: [54]. Mentioned figures: Figs. SN 2.4, SN 2.17, SN 2.18.

3.2 Calibration metrics

BRIER SCORE (BS)/BRIER SKILL SCORE (BSS)

$$BS = \frac{1}{N} \sum_{i=1}^{N} \sum_{k=1}^{C} (p_{ik} - y_{ik})^2$$

VALUE RANGE: [0, 2] ↓

N: number of samples
C: number of classes
p_{ik}: predicted probability for sample x_i and class k
y_{ik}: outcome; y_{ik} = 1 if y_i is equal to k and 0 otherwise

DESCRIPTION
BS ist the mean squared error of a predicted class score and the actual outcome, thus assessing discrimination and calibration in one joint score. It is a proper scoring rule.

VARIANT
Brier Skill Score (BSS): normalizes BS by the BS of a naive system.

DEFINITION
[Gneiting and Raftery, 2007]

METRIC FAMILY

Counting metric	Multi-threshold metric	Distance-based metric	Calibration metric
○	○	○	●

TYPE OF CALIBRATION

Top-label	Marginal	Canonical
○	○	●

RELEVANT PITFALLS
- BS/BSS simultaneously assess the discrimination and calibration performance in one score and can thus only be used for relative assessment of calibration.
- BS is highly prevalence-dependent, implying that scores may drastically change when the prevalence changes (Fig. SN 2.7), i.e., predicted class scores linked to sporadic events have little effect on the score, leading to preference of naive systems in imbalanced settings.
- BS/BSS do not allow for incorporating unequal severity of confusions across classes in discrimination. This implies that these metrics are not applicable when target classes are related on an ordinal scale (Fig. 4b, [Reinke et al., 2021]).

Fig. SN 3.64. Metric profile of Brier Score (BS). The downward arrow in the value range indicates that lower values are better than higher values. Abbreviation: Brier Skill Score (BSS). References: Gneiting and Raftery, 2007: [26], Reinke et al., 2021: [54]. Mentioned figure: Fig. SN 2.7.

CLASS-WISE CALIBRATION ERROR (CWCE)

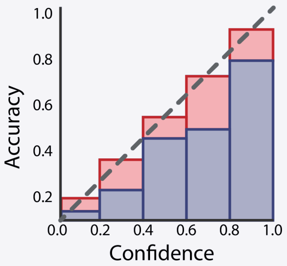

$$CWCE = \frac{1}{C}\sum_{c=1}^{C}\sum_{m=1}^{M}\frac{|B_{c,m}|}{N}\,||Accuracy_c(B_{c,m}) - Confidence_c(B_{c,m})||_p^p$$

N: number of samples; C: number of classes
$B_{c,m}$: bin m for class c
p: determines which L_p calibration error is desired; typically p = 1

VALUE RANGE: [0, 1] ↓

DESCRIPTION

CWCE is an estimator of the marginal calibration error applying binning to estimate the observed probabilities corresponding to a confidence range. It can be reported per class or in an aggregated fashion with class-specific weights reflecting prevalence or importance of classes, for example.

DEFINITION

[Kull et al., 2019; Kumar et al., 2019]

METRIC FAMILY

Counting metric	Multi-threshold metric	Distance-based metric	Calibration metric
○	○	○	●

TYPE OF CALIBRATION

Top-label	Marginal	Canonical
○	●	○

RELEVANT PITFALLS

- The number and size of the bins in the CWCE is not standardized (Fig. SN 2.22).
- CWCE under any binning scheme is biased and underestimates the true CE (Fig. SN 2.6).
- CWCE only measures the calibration quality, not the discrimination.
- Marginal errors do not assess full calibration (Fig. SN 2.6)
- CWCE is dependent on the number of samples (Fig. 5b) and may yield unstable (or arbitrary high) calibration errors. Even classifiers with an CWCE of zero can be miscalibrated (Fig. SN 2.6).
- For CWCE, there are no systematic studies on behavior for imbalanced data.

Fig. SN 3.65. Metric profile of Class-Wise Calibration Error (CWCE). The downward arrow in the value range indicates that lower values are better than higher values. References: Kumar et al., 2019: [41], Kull et al., 2019: [40]. Mentioned figures: Figs. 5b, SN 2.6, SN 2.22.

EXPECTED CALIBRATION ERROR (ECE)

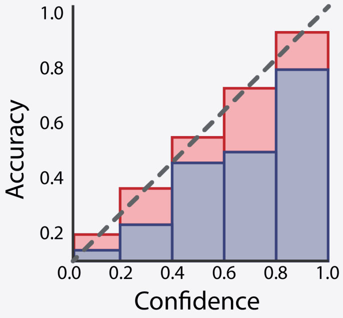

$$\mathrm{ECE} = \sum_{m=1}^{M} \frac{|B_m|}{N} \left\|\mathrm{Accuracy}(B_m) - \mathrm{Confidence}(B_m)\right\|_p^p$$

= Weighted Average

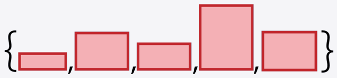

N: number of samples, B_m: bin m
p: determines which L_p calibration error is desired; typically p = 1

VALUE RANGE: [0, 1] ↓

DESCRIPTION

ECE is an estimator for the L_p top-label calibration error. For a binned estimation, it is the weighted average of the absolute difference between the average predicted class score (Confidence) of the top label per bin B_m and the corresponding fraction of correct predictions (Accuracy).

VARIANT

The marginal variant of ECE is CWCE.

DEFINITION

[Naeini et al., 2015]

METRIC FAMILY

Counting metric	Multi-threshold metric	Distance-based metric	Calibration metric
○	○	○	●

TYPE OF CALIBRATION

Top-label	Marginal	Canonical
●	○	○

RELEVANT PITFALLS

- The number and size of the bins in the ECE is not standardized (Fig. SN 2.22).
- ECE under any binning scheme is biased and underestimates the true CE (Fig. SN 2.6).
- ECE only measures the calibration quality, not the discrimination.
- ECE is dependent on the number of samples (Fig. 5b) and may yield unstable (or arbitrary high) calibration errors.
- Top-label errors do not assess full calibration, i.e. even classifiers with an ECE of zero can be miscalibrated (Fig. SN 2.6). Top-label ECE implies an argmax-based decision rule applied to the predicted class scores, which is often not optimal [Maier-Hein et al., 2022].

Fig. SN 3.66. Metric profile of Expected Calibration Error (ECE). The downward arrow in the value range indicates that lower values are better than higher values. References: Maier-Hein et al., 2022: [44], Naeini et al., 2015: [47], Reinke et al., 2021: [54]. Mentioned figures: Figs. 5b, SN 2.6, SN 2.22.

EXPECTED CALIBRATION ERROR KERNEL DENSITY ESTIMATE (ECE^{KDE})

$$ECE^{KDE} = \frac{1}{N}\sum_{j=1}^{N}\left\| \frac{\sum_{i \neq j} k(f(x_j), f(x_i)) e_{y_i}}{\sum_{i \neq j} k(f(x_j), f(x_i))} - f(x_j) \right\|_p^p$$

N: number of samples
k: kernel, e.g. Dirichlet kernel [Popordanoska et al., 2022]
f(x): predicted probability vector, y_i: outcome (one-hot encoded)
e_y: C-dimensional vector with y_i-th entry being 1, else 0
p: determines which L_p calibration error is desired; typically $p \in \{1, 2\}$

VALUE RANGE: [0, 2] ↓

DESCRIPTION
ECE^{KDE} is an estimator for the canonical calibration error. It uses a kernel density estimate in contrast to the binning strategy applied by the standard ECE.

METRIC FAMILY
Counting metric ○ | Multi-threshold metric ○ | Distance-based metric ○ | Calibration metric ●

DEFINITION
[Popordanoska et al., 2022]

TYPE OF CALIBRATION
Top-label ○ | Marginal ○ | Canonical ●

RELEVANT PITFALLS
- ECE^{KDE} does not scale to a large number of classes (problematic for more than 10 classes).
- ECE^{KDE} is a biased estimator and is particularly unreliable for small sample sizes.

Fig. SN 3.67. Metric profile of Expected Calibration Error Kernel Density Estimate (ECE^{KDE}). The downward arrow in the value range indicates that lower values are better than higher values. Abbreviation: Expected Calibration Error (ECE). Reference used in the figure: Popordanoska et al., 2022: [52].

KERNEL CALIBRATION ERROR (KCE)

$$KCE = \left(\mathbb{E}\left((e_y - f(x))^T k(f(x), f(x'))(e_{y'} - f(x'))\right)\right)^{1/2}$$

Example estimator: $$\widehat{KCE} = \left(\binom{N}{2}^{-1} \sum_{i=1}^{N} \sum_{j=i+1}^{N} (e_{y_i} - f(x_i))^T k(f(x_i), f(x_j))(e_{y_j} - f(x_j))\right)^{1/2}$$

N: number of samples; k: matrix-valued kernel; f(x): predicted probability vector; y_i: outcome; e_{y_i}: C-dimensional vector with y_i-th entry being 1, else 0

VALUE RANGE: Kernel dependent; in expectation > 0 but estimator can be arbitrarily negative

DESCRIPTION

KCE measures a canonical calibration error based on an alternative distance function, the "maximum mean discrepancy" (MMD). It is based on a matrix-valued kernel k.

KCE is an unbiased estimator of the calibration error measured by MMD.

DEFINITION

[Widmann et al., 2019; Gruber and Buettner, 2022]

METRIC FAMILY

Counting metric | Multi-threshold metric | Distance-based metric | Calibration metric (selected)

TYPE OF CALIBRATION

Top-label | Marginal | Canonical (selected)

RELEVANT PITFALLS

- KCE may be hard to interpret, also due to negative output values.
- KCE cannot be used as an interpretable estimate of the calibration error and should only be used for comparative calibration assessment.
- KCE depends on nontrivial configuration choices of kernels and associated hyperparameters.
- KCE is computationally expensive.

Fig. SN 3.68. Metric profile of Kernel Calibration Error (KCE). References: Gruber and Buettner, 2022: [28], Widmann et al., 2019: [68].

NEGATIVE LOG LIKELIHOOD (NLL)

Synonym: Cross Entropy Loss

$$NLL = -\frac{1}{N}\sum_{i=1}^{N}\sum_{k=1}^{C} y_{ik} \cdot \log(p_{ik})$$

VALUE RANGE: $[0, \infty)$ ↓

N: number of samples
C: number of classes

p_{ik}: predicted probability for sample x_i and class k
y_{ik}: outcome; $y_{ik} = 1$ if y_i is equal to k and 0 otherwise

DESCRIPTION

NLL is the negative logarithm of a predicted class score and the actual outcome. It is a proper scoring rule that can be used to measure the discrimination and calibration quality in one joint score.

DEFINITION

[Cybenko et al., 1998]

METRIC FAMILY

Counting metric	Multi-threshold metric	Distance-based metric	Calibration metric
○	○	○	●

TYPE OF CALIBRATION

Top-label	Marginal	Canonical
○	○	●

RELEVANT PITFALLS

- NLL simultaneously assesses the discrimination and calibration performance in one score and can thus only be used for relative assessment of calibration.
- NLL introduces a strong penalization of tail probabilities, i.e., overconfident predictions lead to higher losses and conservative models are favored [Popordanoska et al., 2022].
- NLL does not allow for incorporating unequal severity of confusions across classes in discrimination. This implies that the metric is not applicable when target classes are related on an ordinal scale (Fig. 4b, [Reinke et al., 2021]).
- NLL is hard to interpret given no fixed upper bound.

Fig. SN 3.69. Metric profile of Negative Log Likelihood (NLL). The downward arrow in the value range indicates that lower values are better than higher values. References: Cybenko et al., 1998: [14], Popordanoska et al., 2022: [52], Reinke et al., 2021: [54]. Mentioned figure: Fig. 5b.

ROOT BRIER SCORE (RBS)

$$\mathrm{RBS} = \sqrt{\frac{1}{N}\sum_{i=1}^{N}\sum_{k=1}^{C}(p_{ik} - y_{ik})^2}$$

VALUE RANGE: $[0, \sqrt{2}\,]$ ↓

N: number of samples
C: number of classes

p_{ik}: predicted probability for sample x_i and class k
y_{ik}: outcome; y_{ik} = 1 if y_i is equal to k and 0 otherwise

DESCRIPTION

RBS is the square root of the mean squared error of a predicted class score and the actual outcome.

It represents a robust upper bound of the canonical calibration error.

METRIC FAMILY

Counting metric	Multi-threshold metric	Distance-based metric	Calibration metric
○	○	○	●

TYPE OF CALIBRATION

Top-label	Marginal	Canonical
○	○	●

DEFINITION

[Gruber and Buettner, 2022]

RELEVANT PITFALLS

It is not clear how tight the upper bound is, especially for models with low accuracy, given that it is unclear to what extent RBS overestimates the canonical calibration error.

Fig. SN 3.70. Metric profile of Root Brier Score (RBS). The downward arrow in the value range indicates that lower values are better than higher values. Reference: Gruber and Buettner, 2022: [28].

3.3 Localization criteria

BOUNDARY INTERSECTION OVER UNION (BOUNDARY IOU)

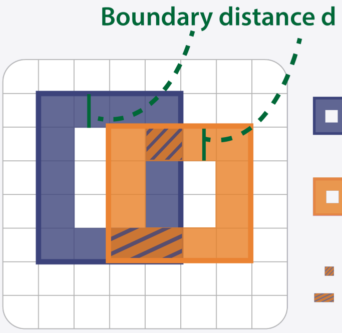

A_d: Pixels of structure A within width d from boundary

B_d: Pixels of structure B within width d from boundary

$A_d \cap B_d$

$$\text{Boundary IoU(A,B)} = \frac{|A_d \cap B_d|}{|A_d| + |B_d| - |A_d \cap B_d|} = \frac{|A_d \cap B_d|}{|A_d \cup B_d|}$$

VALUE RANGE: [0, 1] ↑

LOCALIZATION CRITERION

$\geq \tau$: TP

$< \tau$: FP

DESCRIPTION

Boundary IoU measures the overlap between the predicted and reference boundaries up to a predefined width d. Combined with a localization threshold τ it can be used as a localization criterion.

DEFINITION

[Cheng et al., 2021]

METRIC FAMILY

Counting metric	Multi-threshold metric	Distance-based metric	Localization criterion
○	○	●	●

RELEVANT PITFALLS

- Depending on the choice of the hyperparameter d, Boundary IoU may ignore the overlap and volume of structures (Fig. SN 2.4).
- Boundary IoU is undefined if the reference and prediction are empty, and ad hoc rules need to be established depending on the use case (Fig. SN 2.18).
- Boundary IoU may yield a perfect value for only predicting the boundary pixels, missing the pixels inside the object [Reinke et al., 2021].
- Boundary IoU includes the hyperparameter d, which needs to be chosen wisely (user or use case-defined threshold) [Reinke et al., 2021].
- The localization criterion highly depends on the chosen localization threshold τ.

Fig. SN 3.71. Metric profile of the Boundary Intersection over Union (IoU) localization criterion. The upward arrow in the value range indicates that higher values of Boundary IoU are better than lower values. References: Cheng et al., 2021: [10], Reinke et al., 2021: [54]. Mentioned figures: Figs. SN 2.4, SN 2.18.

CENTER DISTANCE

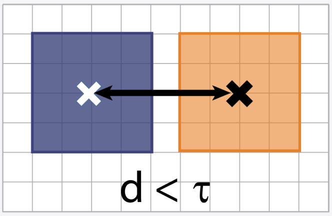

Reference — Prediction

Reference center — Prediction center

VALUE RANGE: $[0, \infty)$ ↓

DESCRIPTION

The Center Distance measures the (typically Euclidean) distance between the reference and predicted center point of an object. The prediction is considered as a hit if the distance is smaller than a predefined threshold τ. Depending on what kind of information the center point is derived from, different definitions are possible, for instance:

- Geometric center of the box/approximation shape,
- Geometric center of a binary mask, i.e., average of positions of all pixels/voxels,
- Center of mass of a binary mask overlaid with the original image, i.e., weighted average of positions of all pixels/voxels with weight equal to (or derived from) the intensity of a particular pixel/voxel.

DEFINITION

[Gurcan et al., 2010]

METRIC FAMILY

Counting metric	Multi-threshold metric	Distance-based metric	Localization criterion
○	○	●	●

RELEVANT PITFALLS

- The Center Distance ignores the overlap between objects [Reinke et al., 2021].
- The Center Distance is often only a rough approximation of the real object center distance because the object center is often computed imprecisely (e.g., as the bounding box center, intensity maximum position, etc.) [Reinke et al., 2021].
- The Center Distance is an error-prone approximation for complex shapes, e.g., tubular-shaped structures [Reinke et al., 2021].
- The localization criterion highly depends on the chosen localization threshold τ.

Fig. SN 3.72. Metric profile of the Center Distance localization criterion. The downward arrow in the value range indicates that lower values of the Center Distance are better than higher values. References: Gurcan et al., 2010: [30], Reinke et al., 2021: [54].

INTERSECTION OVER REFERENCE (IoR)

Synonyms: Pixel-level Sensitivity

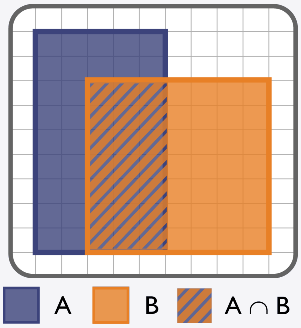

$$\mathrm{IoR}(A,B) = \frac{|A \cap B|}{|A|}$$

VALUE RANGE: [0, 1] ↑

LOCALIZATION CRITERION

$\geq \tau$: TP

$< \tau$: FP

DESCRIPTION

IoR measures the overlap between two structures. It is defined as the pixel-level Sensitivity and only considers the FN pixels (not the FPs). The metric is rather uncommon for segmentation assessment, but combined with a localization τ threshold it can be used as a localization criterion.

DEFINITION

[Maska et al., 2014]

CARDINALITIES

TP	FP	FN	TN
●	○	●	○

METRIC FAMILY

Counting metric	Multi-threshold metric	Distance-based metric	Localization criterion
●	○	○	●

RELEVANT PITFALLS

- IoR is unaware of shapes, boundaries, distances and centers (Fig. 4a, SN 2.5, SN 2.12, Extended Data Fig. 1b).
- IoR penalizes missed pixels more in small objects (Fig. SN 2.10, Extended Data Fig. 1a).
- IoR is undefined if the reference and prediction are empty, and ad hoc rules need to be established depending on the use case (Fig. SN 2.18).
- IoR treats oversegmentation and undersegmentation differently (Fig. SN 2.8).
- A loose IoR-based localization criterion can be deceived by large predictions (Fig. SN 2.23).
- IoR is rather uncommon and can therefore not be used for comparison with other publications.
- The localization criterion highly depends on the chosen localization threshold τ.

Fig. SN 3.73. Metric profile of the Intersection over Reference (IoR) localization criterion. The upward arrow in the value range indicates that higher values of IoR are better than lower values. Abbreviations: False Negative (FN), False Positive (FP), True Negative (TN), True Positive (TP). References: Maška et al., 2014: [45], Reinke et al., 2021: [54]. Mentioned figures: Figs. 4a, SN 2.5, SN 2.8, SN 2.10, SN 2.11, SN 2.12, SN 2.18, SN 2.23, Extended Data Fig. 1b.

MASK/BOX/APPROX INTERSECTION OVER UNION (MASK/BOX/APPROX IoU)

Synonyms: Jaccard Index, Tanimoto Coefficient

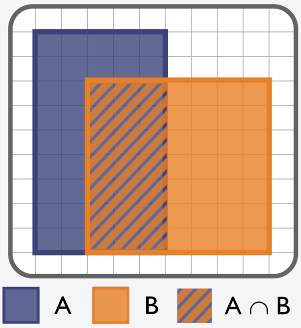

IoU(A,B) = 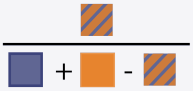

$$= \frac{|A \cap B|}{|A| + |B| - |A \cap B|} = \frac{|A \cap B|}{|A \cup B|}$$

$$= \frac{\text{PPV} \cdot \text{Sensitivity}}{\text{PPV} + \text{Sensitvity} - \text{PPV} \cdot \text{Sensitivity}}$$

LOCALIZATION CRITERION

$\geq \tau$: TP

$< \tau$: FP

DESCRIPTION

IoU measures the overlap between two structures (see above). Combined with a localization threshold, it is a common localization criterion. It is often referred to as **Box IoU** when comparing bounding boxes, **Mask IoU** when comparing segmentation masks, or **Approx IoU** when comparing approximations of objects beyond bounding boxes.

DEFINITION

[Jaccard, 1912]

CARDINALITIES

TP	FP	FN	TN
●	●	●	○

METRIC FAMILY

Counting metric	Multi-threshold metric	Distance-based metric	Localization criterion
●	○	○	●

RELEVANT PITFALLS

- IoU is unaware of shapes, boundaries, distances and centers (Figs. 4a, SN 2.5, SN 2.12, Extended Data Fig. 1b).
- IoU penalizes missed pixels more in small objects (Figs. SN 2.10, SN 2.11, Extended Data Fig. 1a).
- Box IoU is not a good representation of complex or disconnected structures (Fig. SN 2.14).
- IoU is undefined if both the reference and prediction are empty, and ad hoc rules need to be established depending on the use case (Fig. SN 2.18).
- IoU treats oversegmentation and undersegmentation differently (Fig. SN 2.8).
- IoU does not compensate for inter-rater variability (Fig. SN 2.17).
- A loose IoU-based localization criterion can be deceived by large predictions (Fig. SN 2.23).
- IoU behaves differently in 2D and 3D settings. In 3D settings, the additional z-dimension results in a cubical increase in erroneous pixels [Reinke et al., 2021].
- An IoU-based localization criterion may highly penalize multiple predictions for the same reference object [Reinke et al., 2021].
- The localization criterion highly depends on the chosen localization threshold τ.

Fig. SN 3.74. Metric profile of the Mask/Box/Approx Intersection over Union (IoU) localization criterion. Abbreviations: False Negative (FN), False Positive (FP), True Negative (TN), True Positive (TP). References: Jaccard, 1912: [35], Reinke et al., 2021: [54]. Mentioned figures: Figs. 4a, SN 2.5, SN 2.8, SN 2.10, SN 2.11,SN 2.12, SN 2.14, SN 2.17, SN 2.18, SN 2.23, Extended Data Fig. 1a-b.

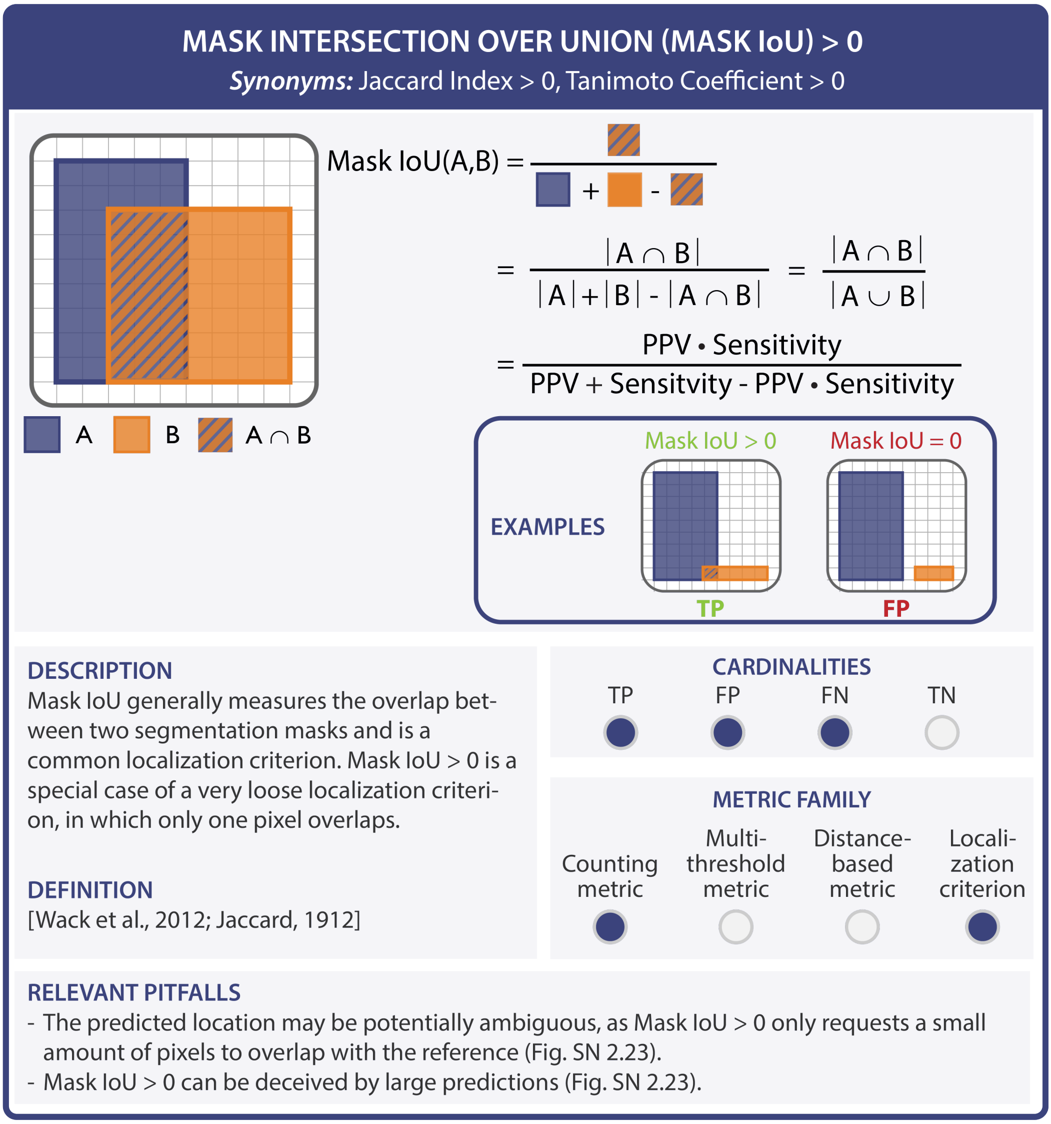

Fig. SN 3.75. Metric profile of the Mask Intersection over Union (IoU) > 0 localization criterion. Abbreviations: False Negative (FN), False Positive (FP), True Negative (TN), True Positive (TP). References: Jaccard, 1912: [35], Wack et al., 2012: [66]. Mentioned figure: Fig. SN 2.23.

POINT INSIDE MASK/BOX/APPROXIMATION

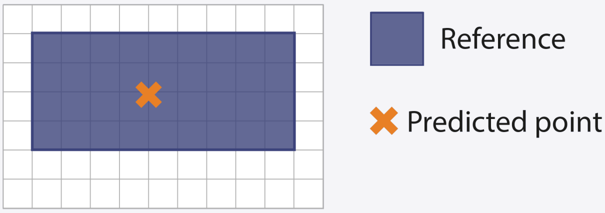

VALUE RANGE: {True, False}

DESCRIPTION

The Point inside Mask/Box/Approximation is a localization criterion that defines a point-based prediction as a hit as long as it is inside the reference object, which may be a mask, bounding box, or other approximation of a structure.

DEFINITION

https://cada.grand-challenge.org/Assessment/

METRIC FAMILY

- Counting metric ○
- Multi-threshold metric ○
- Distance-based metric ○
- Localization criterion ●

RELEVANT PITFALLS

- The Point inside Mask/Box/Approximation criterion does not allow to adjust the localization strictness [Reinke et al., 2021].
- The Point inside Mask/Box/Approximation criterion is only a rough approximation of the object location [Reinke et al., 2021].

Fig. SN 3.76. Metric profile of Point inside Mask/Box/Approximation. References: https://cada.grand-challenge.org/Assessment/, Reinke et al., 2021: [54].

3.4 Assignment strategies

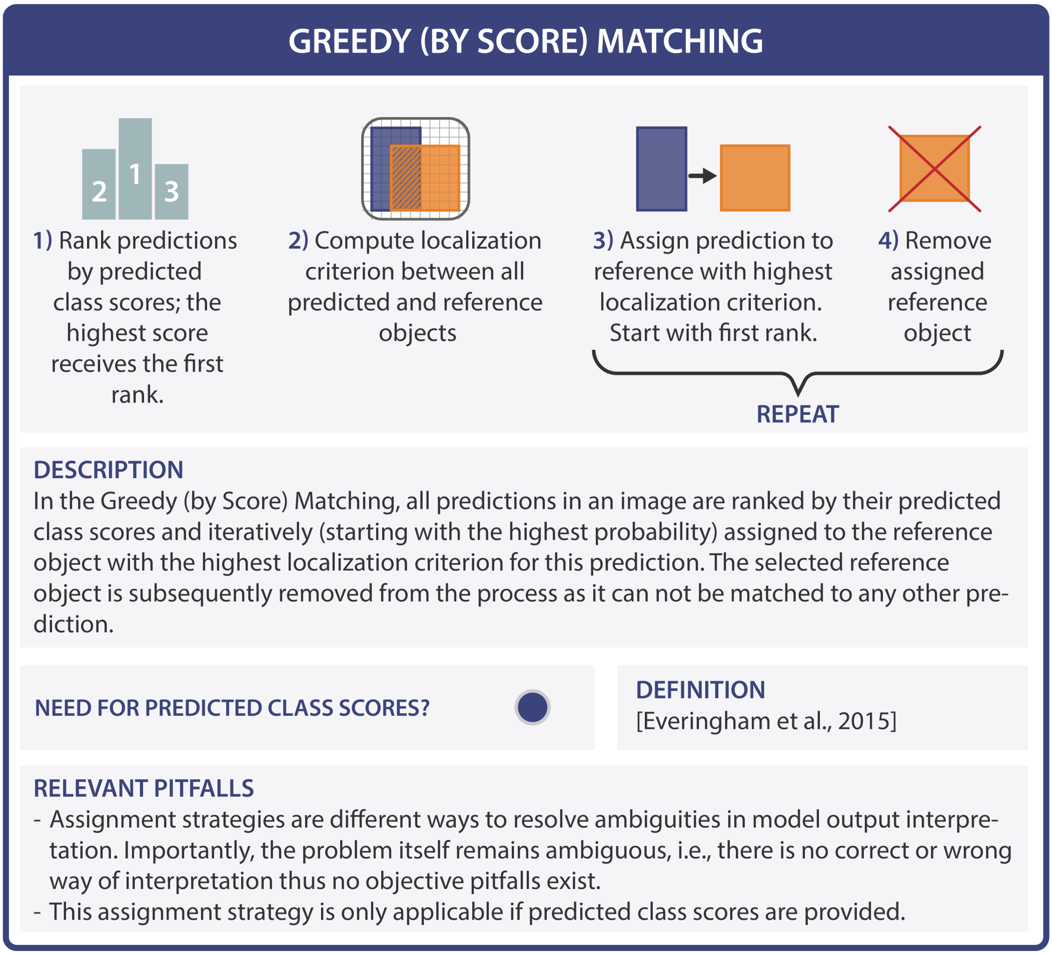

Fig. SN 3.77. Cheat Sheet for the Greedy (by Score) Matching. Reference used in the figure: Everingham et al., 2015: [23].

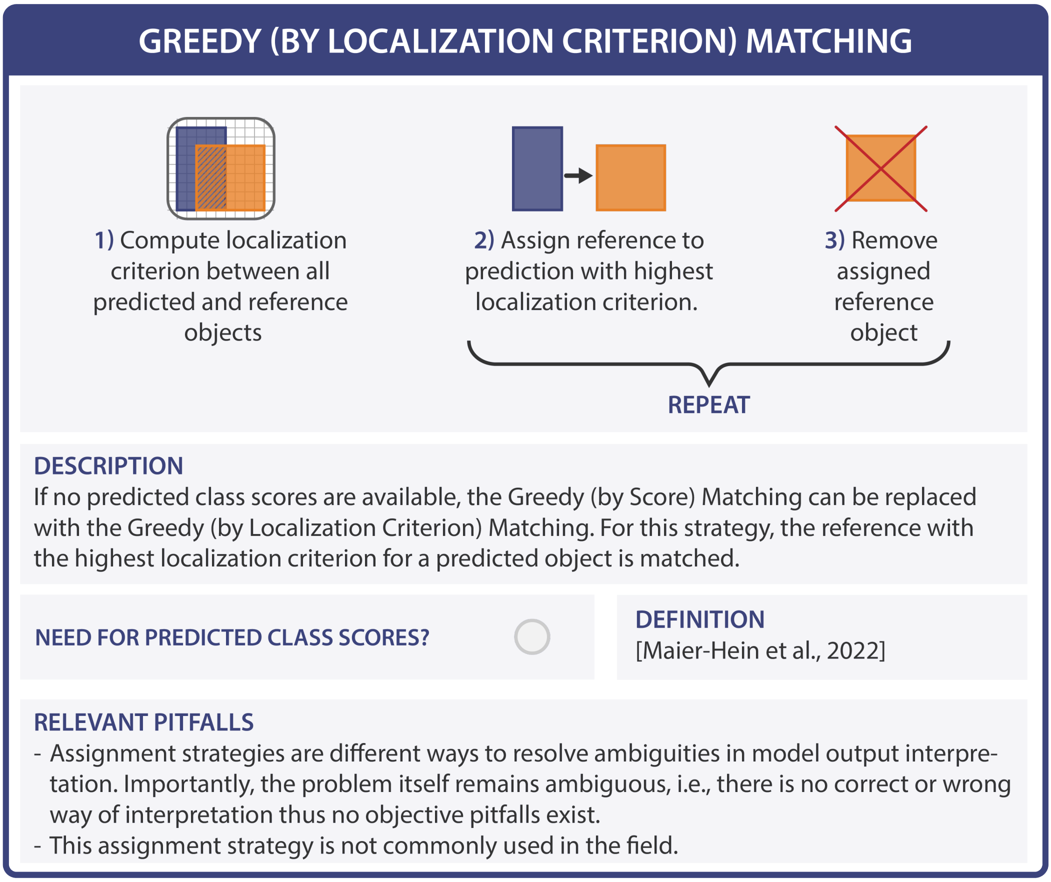

Fig. SN 3.78. Cheat Sheet for the Greedy (by Localization Criterion) Matching. Reference used in the figure: Maier-Hein et al., 2022: [44].

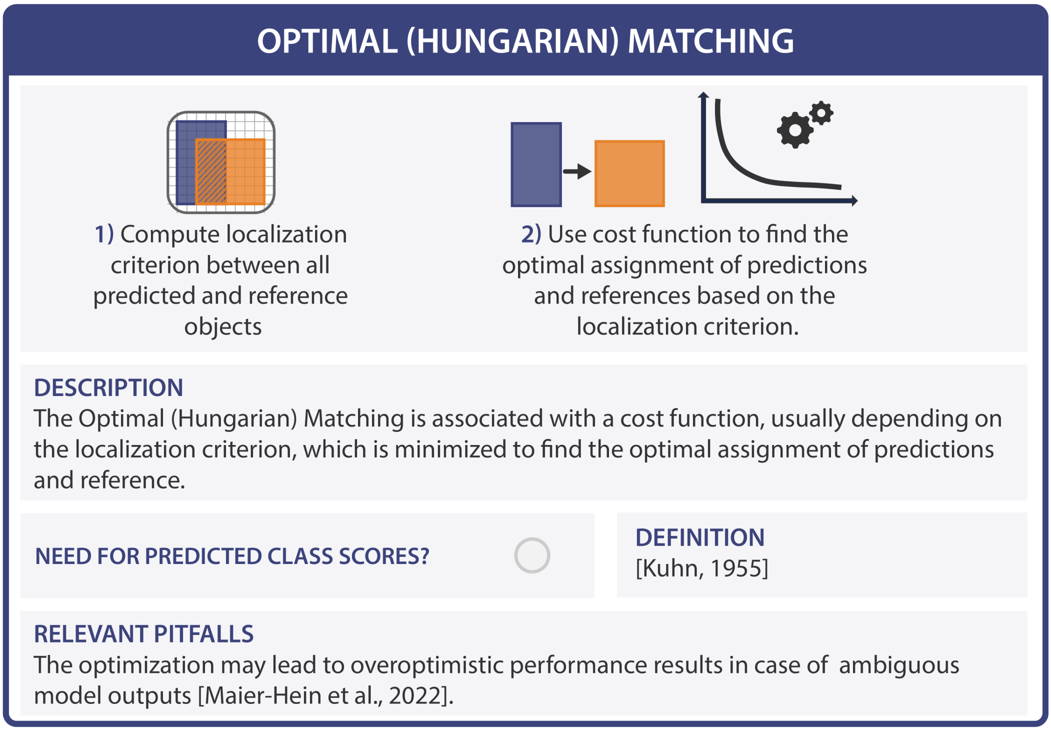

Fig. SN 3.79. Cheat Sheet for the Optimal (Hungarian) Matching. References used in the figure: Kuhn et al., 1955: [38], Maier-Hein et al., 2022: [44].

MATCHING VIA OVERLAP > 0.5

PREREQUISITE: Overlapping predictions are not possible.

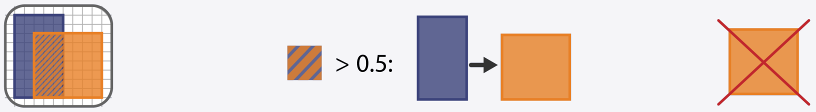

1) Compute overlap-based localization criterion between all predicted and reference objects

2) If the overlap is greater than 0.5, assign prediction to the reference.

3) Remove assigned reference object

REPEAT

DESCRIPTION

If there are no overlapping predictions, complex assignment strategies can be avoided by simply setting the localization criterion to IoU > 0.5.This strategy inherently avoids matching conflicts, because any secondary prediction would by definition have an overlap < 0.5 of the same reference object.

NEED FOR PREDICTED CLASS SCORES?

DEFINITION

[Everingham et al., 2006]

RELEVANT PITFALLS

- Matching via Overlap > 0.5 is unfeasible if overlapping predictions are possible [Maier-Hein et al., 2022].
- Matching via Overlap > 0.5 cannot be applied if a non-overlap based criterion is employed (e.g., Point inside Mask).

Fig. SN 3.80. Cheat Sheet for the Matching via Overlap > 0.5. References used in the figure: Everingham et al., 2006: [21], Maier-Hein et al., 2022: [44].

SUPPL. NOTE 4 ACRONYMS

AI artificial intelligence
AP Average Precision
ASSD Average Symmetric Surface Distance
AUC Area under the Curve
AUROC Area under the Receiver Operating Characteristic Curve
BA Balanced Accuracy
BIAS Biomedical Image Analysis ChallengeS
Boundary IoU Boundary Intersection over Union
BS Brier Score
BSS Brier Skill Score
CI Confidence Interval
clDice centerline Dice Similarity Coefficient
COCO Common Objects in Context
CK Cohen's Kappa
CWCE Class-Wise Calibration Error
DSC Dice Similarity Coefficient
EC Expected Cost
ECE Expected Calibration Error
ECE^{KDE} Expected Calibration Error Kernel Density Estimate
FN False Negative
FP False Positive
FPPI False Positives per Image
FROC Free-Response Receiver Operating Characteristic
HD Hausdorff Distance
HD95 Hausdorff Distance 95th Percentile
InS Instance Segmentation
IoU Intersection over Union
IoR Intersection over Reference
LR+ Positive Likelihood Ratio
KCE Kernel Calibration Error
mAP mean Average Precision
MASD Mean Average Surface Distance
MCC Matthews Correlation Coefficient
MCE Maximum Calibration Error
MICCAI Medical Image Computing and Computer Assisted Interventions
MONAI Medical Open Network for Artificial Intelligence
NaN Not a Number
NB Net Benefit
NPV Negative Predictive Value
NLL Negative Log Likelihood
NSD Normalized Surface Distance
PPV Positive Predictive Value
ObD Object Detection
PQ Panoptic Quality
PR Precision-Recall
RBS Root Brier Score

ROC Receiver Operating Characteristic
SemS Semantic Segmentation
TN True Negative
TNR True Negative Rate
TP True Positive
TPR True Positive Rate
WCK Weighted Cohen's Kappa

REFERENCES

[1] Saeid Asgari Taghanaki, Kumar Abhishek, Joseph Paul Cohen, Julien Cohen-Adad, and Ghassan Hamarneh. Deep semantic segmentation of natural and medical images: a review. *Artificial Intelligence Review*, 54(1):137–178, 2021.

[2] John Attia. Moving beyond sensitivity and specificity: using likelihood ratios to help interpret diagnostic tests. *Australian prescriber*, 26(5):111–113, 2003.

[3] Min Bai and Raquel Urtasun. Deep watershed transform for instance segmentation. In *Proceedings of the IEEE conference on computer vision and pattern recognition*, pages 5221–5229, 2017.

[4] D Bamira and MH Picard. Imaging: Echocardiology—assessment of cardiac structure and function. *Elsevier*, 2018.

[5] Andriy I Bandos, Howard E Rockette, Tao Song, and David Gur. Area under the free-response roc curve (froc) and a related summary index. *Biometrics*, 65(1):247–256, 2009.

[6] Miroslav Beneš and Barbara Zitová. Performance evaluation of image segmentation algorithms on microscopic image data. *Journal of microscopy*, 257(1):65–85, 2015.

[7] Christopher M Bishop and Nasser M Nasrabadi. *Pattern recognition and machine learning*, volume 4. Springer, 2006.

[8] Bernice B Brown. Delphi process: a methodology used for the elicitation of opinions of experts. Technical report, Rand Corp Santa Monica CA, 1968.

[9] Chang Cao, Davide Chicco, and Michael M Hoffman. The mcc-f1 curve: a performance evaluation technique for binary classification. *arXiv preprint arXiv:2006.11278*, 2020.

[10] Bowen Cheng, Ross Girshick, Piotr Dollár, Alexander C Berg, and Alexander Kirillov. Boundary iou: Improving object-centric image segmentation evaluation. In *Proceedings of the IEEE/CVF Conference on Computer Vision and Pattern Recognition*, pages 15334–15342, 2021.

[11] Davide Chicco and Giuseppe Jurman. The advantages of the matthews correlation coefficient (mcc) over f1 score and accuracy in binary classification evaluation. *BMC genomics*, 21(1):1–13, 2020.

[12] Nancy Chinchor. Muc-4 evaluation metrics. In *Proceedings of the 4th Conference on Message Understanding*, MUC4 '92, page 22–29, USA, 1992. Association for Computational Linguistics. ISBN 1558602739. doi: 10.3115/1072064.1072067. URL https://doi.org/10.3115/1072064.1072067.

[13] Jacob Cohen. A coefficient of agreement for nominal scales. *Educational and psychological measurement*, 20(1):37–46, 1960.

[14] George Cybenko, Dianne P O'Leary, and Jorma Rissanen. *The Mathematics of Information Coding, Extraction and Distribution*, volume 107. Springer Science & Business Media, 1998.

[15] Jesse Davis and Mark Goadrich. The relationship between precision-recall and roc curves. In *Proceedings of the 23rd international conference on Machine learning*, pages 233–240, 2006.

[16] Bert De Brabandere, Davy Neven, and Luc Van Gool. Semantic instance segmentation with a discriminative loss function. *arXiv preprint arXiv:1708.02551*, 2017.

[17] Jeffrey De Fauw, Joseph R Ledsam, Bernardino Romera-Paredes, Stanislav Nikolov, Nenad Tomasev, Sam Blackwell, Harry Askham, Xavier Glorot, Brendan O'Donoghue, Daniel Visentin, et al. Clinically applicable deep learning for diagnosis and referral in retinal disease. *Nature medicine*, 24(9):1342–1350, 2018.

[18] Rosario Delgado and Xavier-Andoni Tibau. Why cohen's kappa should be avoided as performance measure in classification. *PloS one*, 14(9):e0222916, 2019.

[19] Elizabeth R DeLong, David M DeLong, and Daniel L Clarke-Pearson. Comparing the areas under two or more correlated receiver operating characteristic curves: a nonparametric approach. *Biometrics*, pages 837–845, 1988.

[20] Lee R Dice. Measures of the amount of ecologic association between species. *Ecology*, 26(3):297–302, 1945.

[21] Mark Everingham, Andrew Zisserman, Christopher KI Williams, Luc Van Gool, Moray Allan, Christopher M Bishop, Olivier Chapelle, Navneet Dalal, Thomas Deselaers, Gyuri Dorkó, et al. The 2005 pascal visual object classes challenge. In *Machine Learning Challenges. Evaluating Predictive Uncertainty, Visual Object Classification, and Recognising Tectual Entailment: First PASCAL Machine Learning Challenges Workshop, MLCW 2005, Southampton, UK, April 11-13, 2005, Revised Selected Papers*, pages 117–176. Springer, 2006.

[22] Mark Everingham, Luc Van Gool, Christopher KI Williams, John Winn, and Andrew Zisserman. The pascal visual object classes (voc) challenge. *International journal of computer vision*, 88(2):303–338, 2010.

[23] Mark Everingham, SM Ali Eslami, Luc Van Gool, Christopher KI Williams, John Winn, and Andrew Zisserman. The pascal visual object classes challenge: A retrospective. *International journal of computer vision*, 111(1):98–136, 2015.

[24] Luciana Ferrer. Analysis and comparison of classification metrics. *arXiv preprint arXiv:2209.05355*, 2022.

[25] Naiyu Gao, Yanhu Shan, Yupei Wang, Xin Zhao, Yinan Yu, Ming Yang, and Kaiqi Huang. Ssap: Single-shot instance segmentation with affinity pyramid. In *Proceedings of the IEEE/CVF International Conference on Computer Vision*, pages 642–651, 2019.

[26] Tilmann Gneiting and Adrian E Raftery. Strictly proper scoring rules, prediction, and estimation. *Journal of the American statistical Association*, 102(477):359–378, 2007.

[27] Margherita Grandini, Enrico Bagli, and Giorgio Visani. Metrics for multi-class classification: an overview. *arXiv preprint arXiv:2008.05756*, 2020.

[28] Sebastian Gruber and Florian Buettner. Trustworthy deep learning via proper calibration errors: A unifying approach for quantifying the reliability of predictive uncertainty. *arXiv preprint arXiv:2203.07835*, 2022.

[29] Chuan Guo, Geoff Pleiss, Yu Sun, and Kilian Q Weinberger. On Calibration of Modern Neural Networks. *ICML*, page 10, 2017.

[30] Metin N Gurcan, Anant Madabhushi, and Nasir Rajpoot. Pattern recognition in histopathological images: An icpr 2010 contest. In *International Conference on Pattern Recognition*, pages 226–234. Springer, 2010.

[31] James A Hanley and Barbara J McNeil. The meaning and use of the area under a receiver operating characteristic (roc) curve. *Radiology*, 143(1):29–36, 1982.

[32] Trevor Hastie, Robert Tibshirani, Jerome H Friedman, and Jerome H Friedman. *The elements of statistical learning: data mining, inference, and prediction*, volume 2. Springer, 2009.

[33] Peter Hirsch, Lisa Mais, and Dagmar Kainmueller. Patchperpix for instance segmentation. *arXiv preprint arXiv:2001.07626*, 2020.

[34] Daniel P Huttenlocher, Gregory A. Klanderman, and William J Rucklidge. Comparing images using the hausdorff distance. *IEEE Transactions on pattern analysis and machine intelligence*, 15(9):850–863, 1993.

[35] Paul Jaccard. The distribution of the flora in the alpine zone. 1. *New phytologist*, 11(2):37–50, 1912.

[36] Alexander Kirillov, Kaiming He, Ross Girshick, Carsten Rother, and Piotr Dollár. Panoptic segmentation. In *Proceedings of the IEEE/CVF Conference on Computer Vision and Pattern Recognition*, pages 9404–9413, 2019.

[37] Jonathan Krause, Varun Gulshan, Ehsan Rahimy, Peter Karth, Kasumi Widner, Greg S Corrado, Lily Peng, and Dale R Webster. Grader variability and the importance of reference standards for evaluating machine learning models for diabetic retinopathy. *Ophthalmology*, 125(8):1264–1272, 2018.

[38] Harold W Kuhn. The hungarian method for the assignment problem. *Naval research logistics quarterly*, 2(1-2):83–97, 1955.

[39] Victor Kulikov and Victor Lempitsky. Instance segmentation of biological images using harmonic embeddings. In *Proceedings of the IEEE/CVF Conference on Computer Vision and Pattern Recognition*, pages 3843–3851, 2020.

[40] Meelis Kull, Miquel Perello Nieto, Markus Kängsepp, Telmo Silva Filho, Hao Song, and Peter Flach. Beyond temperature scaling: Obtaining well-calibrated multi-class probabilities with dirichlet calibration. *Advances in neural information processing systems*, 32, 2019.

[41] Ananya Kumar, Percy S Liang, and Tengyu Ma. Verified uncertainty calibration. *Advances in Neural Information Processing Systems*, 32, 2019.

[42] Tsung-Yi Lin, Michael Maire, Serge Belongie, James Hays, Pietro Perona, Deva Ramanan, Piotr Dollár, and C Lawrence Zitnick. Microsoft coco: Common objects in context. In *European conference on computer vision*, pages 740–755. Springer, 2014.

[43] Lena Maier-Hein, Matthias Eisenmann, Annika Reinke, Sinan Onogur, Marko Stankovic, Patrick Scholz, Tal Arbel, Hrvoje Bogunovic, Andrew P Bradley, Aaron Carass, et al. Why rankings of biomedical image analysis competitions should be interpreted with care. *Nature communications*, 9(1):1–13, 2018. With this comprehensive analysis of biomedical image analysis competitions (challenges), the authors initiated a shift in how such challenges are designed, performed, and reported in the biomedical domain. Its concepts and guidelines have been adopted by reputed organizations such as MICCAI.

[44] Lena Maier-Hein, Annika Reinke, Evangelia Christodoulou, Ben Glocker, Patrick Godau, Fabian Isensee, Jens Kleesiek, Michal Kozubek, Mauricio Reyes, Michael A Riegler, et al. Metrics reloaded: Pitfalls and recommendations for image analysis validation. *arXiv preprint arXiv:2206.01653*, 2022.

[45] Martin Maška, Vladimír Ulman, David Svoboda, Pavel Matula, Petr Matula, Cristina Ederra, Ainhoa Urbiola, Tomás España, Subramanian Venkatesan, Deepak MW Balak, et al. A benchmark for comparison of cell tracking algorithms. *Bioinformatics*, 30(11):1609–1617, 2014.

[46] Brian W Matthews. Comparison of the predicted and observed secondary structure of t4 phage lysozyme. *Biochimica et Biophysica Acta (BBA)-Protein Structure*, 405(2):442–451, 1975.

[47] Mahdi Pakdaman Naeini, Gregory Cooper, and Milos Hauskrecht. Obtaining well calibrated probabilities using bayesian binning. In *Twenty-Ninth AAAI Conference on Artificial Intelligence*, 2015.

[48] Ying-Hwey Nai, Bernice W Teo, Nadya L Tan, Sophie O'Doherty, Mary C Stephenson, Yee Liang Thian, Edmund Chiong, and Anthonin Reilhac. Comparison of metrics for the evaluation of medical segmentations using prostate mri dataset. *Computers in Biology and Medicine*, 134:104497, 2021.

[49] Prashant Nasa, Ravi Jain, and Deven Juneja. Delphi methodology in healthcare research: how to decide its appropriateness. *World Journal of Methodology*, 11(4):116, 2021.

[50] Stanislav Nikolov, Sam Blackwell, Alexei Zverovitch, Ruheena Mendes, Michelle Livne, Jeffrey De Fauw, Yojan Patel, Clemens Meyer, Harry Askham, Bernadino Romera-Paredes, et al. Clinically applicable segmentation of head and neck anatomy for radiotherapy: deep learning algorithm development and validation study. *Journal of Medical Internet Research*, 23(7):e26151, 2021.

[51] Hung Viet Pham, Shangshu Qian, Jiannan Wang, Thibaud Lutellier, Jonathan Rosenthal, Lin Tan, Yaoliang Yu, and Nachiappan Nagappan. Problems and opportunities in training deep learning software systems: An analysis of variance. In *Proceedings of the 35th IEEE/ACM international conference on automated software engineering*, pages 771–783, 2020.

[52] Teodora Popordanoska, Raphael Sayer, and Matthew B Blaschko. A consistent and differentiable lp canonical calibration error estimator. In *Advances in Neural Information Processing Systems*, 2022.

[53] David Martin Ward Powers. The problem with kappa. In *Proceedings of the 13th Conference of the European Chapter of the Association for Computational Linguistics*, pages 345–355, 2012.

[54] Annika Reinke, Matthias Eisenmann, Minu D Tizabi, Carole H Sudre, Tim Rädsch, Michela Antonelli, Tal Arbel, Spyridon Bakas, M Jorge Cardoso, Veronika Cheplygina, Keyvan Farahani, Ben Glocker, Doreen Heckmann-Nötzel, Fabian Isensee, Pierre Jannin, Charles Kahn, Jens Kleesiek, Tahsin Kurc, Michal Kozubek, Bennett A Landman, Geert Litjens, Klaus Maier-Hein, Anne L Martel, Henning Müller, Jens Petersen, Mauricio Reyes, Nicola Rieke, Bram Stieltjes, Ronald M Summers, Sotirios A Tsaftaris, Bram van Ginneken, Annette Kopp-Schneider, Paul Jäger, and Lena Maier-Hein. Common limitations of image processing metrics: A picture story. *arXiv preprint arXiv:2104.05642*, 2021.

[55] Azriel Rosenfeld and John L Pfaltz. Sequential operations in digital picture processing. *Journal of the ACM (JACM)*, 13 (4):471–494, 1966.

[56] Anindo Saha, Joeran Bosma, Jasper Linmans, Matin Hosseinzadeh, and Henkjan Huisman. Anatomical and diagnostic bayesian segmentation in prostate mri – should different clinical objectives mandate different loss functions? *arXiv preprint arXiv:2110.12889*, 2021.

[57] Suprosanna Shit, Johannes C Paetzold, Anjany Sekuboyina, Ivan Ezhov, Alexander Unger, Andrey Zhylka, Josien PW Pluim, Ulrich Bauer, and Bjoern H Menze. cldice-a novel topology-preserving loss function for tubular structure segmentation. In *Proceedings of the IEEE/CVF Conference on Computer Vision and Pattern Recognition*, pages 16560–16569, 2021.

[58] Abdel Aziz Taha and Allan Hanbury. Metrics for evaluating 3d medical image segmentation: analysis, selection, and tool. *BMC medical imaging*, 15(1):1–28, 2015. The paper discusses the importance of effective metrics for evaluating the accuracy of 3D medical image segmentation algorithms. The authors analyze existing metrics, propose a selection methodology, and develop a tool to aid researchers in choosing appropriate evaluation metrics based on the specific characteristics of the segmentation task.

[59] Abdel Aziz Taha, Allan Hanbury, and Oscar A Jimenez del Toro. A formal method for selecting evaluation metrics for image segmentation. In *2014 IEEE international conference on image processing (ICIP)*, pages 932–936. IEEE, 2014.

[60] Alaa Tharwat. Classification assessment methods. *Applied Computing and Informatics*, 2020.

[61] Juozas Vaicenavicius, David Widmann, Carl Andersson, Fredrik Lindsten, Jacob Roll, and Thomas Schön. Evaluating model calibration in classification. In *The 22nd International Conference on Artificial Intelligence and Statistics*, pages 3459–3467. PMLR, 2019.

[62] Bram Van Ginneken, Samuel G Armato III, Bartjan de Hoop, Saskia van Amelsvoort-van de Vorst, Thomas Duindam, Meindert Niemeijer, Keelin Murphy, Arnold Schilham, Alessandra Retico, Maria Evelina Fantacci, et al. Comparing and combining algorithms for computer-aided detection of pulmonary nodules in computed tomography scans: the anode09 study. *Medical image analysis*, 14(6):707–722, 2010.

[63] C Van Rijsbergen. Information retrieval: theory and practice. In *Proceedings of the Joint IBM/University of Newcastle upon Tyne Seminar on Data Base Systems*, volume 79, 1979.

[64] Andrew J Vickers and Elena B Elkin. Decision curve analysis: a novel method for evaluating prediction models. *Medical Decision Making*, 26(6):565–574, 2006.

[65] Andrew J Vickers, Ben Van Calster, and Ewout W Steyerberg. Net benefit approaches to the evaluation of prediction models, molecular markers, and diagnostic tests. *bmj*, 352, 2016.

[66] David S Wack, Michael G Dwyer, Niels Bergsland, Carol Di Perri, Laura Ranza, Sara Hussein, Deepa Ramasamy, Guy Poloni, and Robert Zivadinov. Improved assessment of multiple sclerosis lesion segmentation agreement via detection and outline error estimates. *BMC medical imaging*, 12(1):1–10, 2012.

[67] Matthijs J Warrens. Some paradoxical results for the quadratically weighted kappa. *Psychometrika*, 77(2):315–323, 2012.

[68] David Widmann, Fredrik Lindsten, and Dave Zachariah. Calibration tests in multi-class classification: A unifying framework. *Advances in Neural Information Processing Systems*, 32, 2019.

[69] Manuel Wiesenfarth, Annika Reinke, Bennett A Landman, Matthias Eisenmann, Laura Aguilera Saiz, M Jorge Cardoso, Lena Maier-Hein, and Annette Kopp-Schneider. Methods and open-source toolkit for analyzing and visualizing challenge results. *Scientific Reports*, 11(1):1–15, 2021.

[70] Varduhi Yeghiazaryan and Irina Voiculescu. An overview of current evaluation methods used in medical image segmentation. *Department of Computer Science, University of Oxford*, 2015.

[71] Varduhi Yeghiazaryan and Irina D Voiculescu. Family of boundary overlap metrics for the evaluation of medical image segmentation. *Journal of Medical Imaging*, 5(1):015006, 2018.

[72] Qiuming Zhu. On the performance of matthews correlation coefficient (mcc) for imbalanced dataset. *Pattern Recognition Letters*, 136:71–80, 2020.
